# 2023 CSACI annual scientific meeting book of abstracts

**DOI:** 10.1186/s13223-023-00866-4

**Published:** 2024-03-05

## Allergic rhinitis/asthma

## 1 Occupational asthma in Ontario, Canada (2000–2022): a retrospective, clinic-based study evaluating sex differences

### Felix Chan^1^, Victor Nguyen^1^, Susan Tarlo^1,2^

#### ^1^Department of Medicine, University of Toronto, Toronto, ON; ^2^Toronto Western Hospital, University Health Network, Toronto, ON

##### Correspondence: Felix Chan

*Allergy, Asthma & Clinical Immunology* 2024, **20(Suppl 1):**1

**Background:** Occupational asthma accounts for nearly 18% of cases of adult-onset asthma, but prevalence rates vary between men and women. This may be due to sex distributions in industries and occupations associated with developing occupational asthma. The purpose of our study was to assess sex differences in occupational and clinical characteristics of patients with occupational asthma.

**Methods:** We conducted a retrospective analysis of occupational and clinical data (including respiratory and allergy investigations) from patients with occupational asthma assessed at two tertiary care hospitals in Toronto, Canada, between 2000 to 2022. Our research questions were to elucidate sex differences in time to onset and diagnosis of occupational asthma and severity of occupational asthma, measured by spirometry and methacholine challenge testing.

**Results:** Our study population included 255 patients with occupational asthma (159 males [62%] and 96 females [38%]). Males were more likely to work in the automotive industry (22% vs 4%, P < 0.05) and have had their employment terminated due to occupational symptoms (24% vs 3%, P < 0.05). Females predominated in healthcare (15% vs 1%, P < 0.001) and were more likely to have never smoked cigarettes (65% vs 47%, P < 0.05). Males tended to report a longer latency period between occupational exposure and development of asthmatic symptoms (36 months vs 18 months, P = 0.11) and a longer time to diagnosis (24 months vs 14 months, P = 0.08). In never-smokers, the mean FEV1/FVC ratio was significantly greater in females compared to males (75.90 vs 68.75, P < 0.001). There were no significant differences in PEF work variability, severity of airway hyperresponsiveness by methacholine challenge testing, and positivity to allergy skin tests.

**Conclusions:** Though some limitations exist, our findings substantiate historical sex difference trends in occupational asthma distribution and exposures and suggest a longer time to diagnosis and increased disease severity in men compared to women.

## 2 Early experience with tezepelumab in patients with severe asthma: a case series

### Mariam Eldaba, Hoang Pham

#### University of Ottawa, Ottawa, ON

##### **Correspondence:** Mariam Eldaba

*Allergy, Asthma & Clinical Immunology* 2024, **20(Suppl 1):**2

**Background:** Asthma is a heterogeneous disease with a plethora of phenotypes [1]. An estimated 30% of Type 2 high (T2-High) asthma is uncontrolled despite demonstrated effectiveness of multiple downstream biologic medications [2]. However, novel tezepelumab offered a new approach with more upstream blockade of TSLP [3].

**Methods:** We describe 24 adult patients who started tezepelumab for severe asthma between October 2022 and July 2023. The primary objective was a minimal clinical important difference (MCID) of 0.5 reduction in the Asthma Control Questionnaire 6 (ACQ6) from baseline and up to 6 months from initiation of tezepelumab. Secondary objectives included an increase in forced expiratory volume in 1 s (FEV1) of 5% or 100 mL, a 20% decrease in fractional exhaled nitric oxide (FeNO), a reduction in exacerbations, and an 8.9-point reduction in Sinonasal Outcome Test-22 (SNOT22).

**Results:** Mean age 60 [± 15] years, 58% female, 95% T2 high, eosinophils > 300 cells/microL in 45% and high IgE > 100 kU/L in 42%. 16% had nasal polyps and 58% had perennial allergies. 37% were on previous biologics. They were followed for a mean of 3 [± 1.7] months. 58% met the MCID for ACQ6 with a mean reduction of 0.8 [± 1.1]. 25% improved FEV1 by 5% and 5 patients had an over 130 ml improvement. Half the patients had a 20% FeNO reduction with mean decrease of 11 [± 28] points. As for SNOT22, 50% reached the target reduction of 8.9-points with a mean improvement of 9 points (± 14.5). The mean reduction in the number of exacerbations was 1 [± 0.9]. Tezepelumab was well-tolerated with only one patient reporting worsened migraines.

**Conclusions:** Tezepelumab for the treatment of severe asthma was well-tolerated with significant and rapid improvement of symptoms and eosinophilic inflammation in 50% of patients within the first few months of treatment. Longer follow-up will allow for further assessment of intermediate and long-term outcomes.

**References**

1. Moore WC, Meyers DA, Wenzel SE, et al. Identification of asthma phenotypes using cluster analysis in the Severe Asthma Research Program. Am J Respir Crit Care Med 2010; 181:315.
2. Fahy JV. Type 2 inflammation in asthma–present in most, absent in many. Nat Rev Immunol. 2015 Jan;15(1):57–65.
3. Chan R, Stewart K, Misirovs R, Lipworth BJ. Targeting Downstream Type 2 Cytokines or Upstream Epithelial Alarmins for Severe Asthma. J Allergy Clin Immunol Pract. 2022;10(6):1497–1505.

## 3 Impact of age of asthma onset on the long-term efficacy of dupilumab in patients with moderate-to-severe type 2 asthma: LIBERTY ASTHMA TRAVERSE study

### William W. Busse^1^, Monica Kraft^2^, Christian Domingo^3^, Ines de Mir^4^, Diego Maselli^5^, Changming Xia^6^, Nami Pandit-Abid^7^, Xavier Soler^6^, Juby A. Jacob-Nara^7^, Harry Sacks^6^, Paul J. Rowe^7^, Sven Sorensen^8^, Yamo Deniz^6^

#### ^1^UW Allergy, Pulmonary and Critical Care Medicine, University of Wisconsin School of Medicine and Public Health, Madison, WI, USA; ^2^Icahn School of Medicine at Mount Sinai, New York, NY, USA; ^3^Pulmonary Service, Corporació Sanitària Parc Taulí, Sabadell, Universitat Autònoma de Barcelona (UAB), Barcelona, Spain; ^4^Hospital Vall d’Hebron, Barcelona, Spain; ^5^UT Health at San Antonio, San Antonio, TX, USA; ^6^Regeneron Pharmaceuticals Inc., Tarrytown, NY, USA; ^7^Sanofi, Bridgewater, NJ, USA; 8. Sanofi, Mississauga, ON

##### **Correspondence:** Sven Sorensen

*Allergy, Asthma & Clinical Immunology* 2024, **20(Suppl 1):**3

**Background:** The impact of age of asthma onset on efficacy of dupilumab, a fully monoclonal antibody that blocks interleukin-4/13 signalling, is unknown. In phase 3 LIBERTY ASTHMA QUEST (NCT02414854), dupilumab reduced asthma exacerbations and improved lung function in patients with moderate-to-severe asthma. The LIBERTY ASTHMA TRAVERSE open-label extension study (NCT02134028) showed the long-term safety and efficacy of dupilumab. This post hoc analysis assessed the impact of age of asthma onset on dupilumab’s efficacy in patients from QUEST who enrolled in TRAVERSE.

**Methods:** In QUEST, patients received dupilumab 200/300 mg q2w or placebo for 52 weeks. During TRAVERSE, all patients received dupilumab 300 mg q2w for up to 96 weeks. Here we examined unadjusted annualized severe asthma exacerbation rates (AER) and change from parent study baseline (PSBL) in pre-bronchodilator forced expiratory volume in 1 s (pre-BD FEV_1_) throughout QUEST and TRAVERSE in patients with moderate-to-severe type 2 asthma (defined as blood eosinophil count ≥ 150 cells/mL or FeNO ≥ 20 ppb at PSBL), stratified by age of asthma onset (< 18 years [n = 465], 18 to 40 years [n = 450], > 40 years [n = 364]).

**Results:** During QUEST, dupilumab reduced severe exacerbations to 0.502 (< 18 years), 0.485 (18 to 40 years), and 0.377 (> 40 years). These reductions were sustained throughout TRAVERSE (Week 48: 0.392, 0.301, 0.230 and Weeks 48–96: 0.333, 0.191, 0.174, respectively). Dupilumab also improved pre-BD FEV_1_ from PSBL across all subgroups by mean (SD) 0.36 (0.51) L (< 18 years), 0.39 (0.45) L (18 to 40 years), and by 0.34 (0.45) L (> 40 years) at QUEST Week 52. These improvements were sustained throughout TRAVERSE (Week 8: 0.38 [0.51], 0.36 [0.45], 0.37 [0.47] L; Week 48: 0.43 [0.59], 0.40 [0.54], 0.36 [0.47] L; Week 96: 0.37 [0.56], 0.32 [0.43], 0.34 [0.45], respectively).

**Conclusions:** Dupilumab reduced severe exacerbations and improved lung function regardless of age of asthma onset.

## 4 Transforming asthma management and accessibility: the role of virtual care—a scoping review

### Danielle Ben-Shoshan^1^, Mariah Rodriguez-Imbarlina^2^, Jennifer Penner Protudjer^3^, Alexander Singer^4^, Elissa Abrams^4^

#### ^1^Brock University, St. Catharine’s, ON; ^2^Faculty of Health Sciences, Michael G. DeGroote School of Medicine, Hamilton, ON; ^3^Dept Foods and Human Nutritional Sciences, University of Manitoba, Winnipeg, MB; ^4^Dept Pediatrics and Child Health, University of Manitoba, Winnipeg, MB

##### **Correspondence:** Danielle Ben-Shoshan

*Allergy, Asthma & Clinical Immunology* 2024, **20(Suppl 1):**4

**Background:** Asthma is a multifaceted chronic illness that affects 262 million people and has resulted in > 500,000 deaths world-wide. Fortunately, the morbidity, mortality and costs associated with asthma are mostly preventable through disease treatment and management. The COVID-19 pandemic largely shifted healthcare from in-person to virtual delivery, providing the opportunity to evaluate whether virtual care (VC) is associated with effective asthma management and accessibility to treatment.

To this end, we performed a scoping review of the peer-reviewed literature on virtual asthma care and asthma management and accessibility to treatment within a health equity lens.

**Methods:** Our scoping review followed the Arksey and O’Mallley framework. Through consultation with a health sciences librarian, search terms included asthma, telehealth, telemedicine, and virtual care; and, which were searched in four distinct databases, namely OVID Medline, Web of Science, EMBASE, and CINAHL, in publications from 2010 onward. Additional inclusion criteria were English language studies and original publications (including published conference abstracts) only. Exclusion criteria were studies of conditions other than asthma.

The search, scanning, and extraction of data was completed by two reviewers, using Covidence, between May 2023–July 2023, and who were blinded at each stage (title and abstract screening; full text screening). Screening conflicts were handled with input from the project supervisors.

**Results:** To date, 1239 total studies have been screened for titles and abstracts, of which 117 (16.5%) have been advanced to full text screening. Full text screening is underway, and is expected to be completed by the end of July 2023.

**Conclusions:** The title and abstract screening has highlighted that while VC may have a positive or no effect on asthma care, inaccessibility continues to persist as new barriers are introduced in VC. Providers should continue to foster digital equity by seeking solutions to close gaps in this form of care.

## 5 Non-allergist physician algorithm for prescribing allergen immunotherapy

### Alan Kaplan^1^, Susan Waserman^2^, Douglas Mack^3^, Robert Hauptman^1^, Anne Ellis^4^, Lauren Mack^5^, Spiro Photopolous^1^, Juan C. Ruiz^6^, Phillipe Begin^7^, Taft Micks^8^, Harold Kim^9^

#### ^1^Family Physician Airways Group of Canada, Stouffville, ON; ^2^Division Director, Clinical Immunology and Allergy McMaster University, Hamilton, ON; ^3^Allergy consultant McMaster University, Hamilton, ON; ^4^Queens University, Kingston, ON; ^5^University of Cork, Cork, Ireland; ^6^University of British Columbia, Vancouver, BC; ^7^University of Montreal Health Centre, Montreal, QC; ^8^University of Manitoba, Brandon, MB; ^9^University of Western Ontario, Kitchener, ON

##### **Correspondence:** Alan Kaplan

*Allergy, Asthma & Clinical Immunology* 2024, **20(Suppl 1):**5

**Background:** Allergic rhinitis (AR) affects the health and wellbeing of 20–25% of Canadians, with additional detrimental effects on sleep, cognition, mood, mental health and learning. However, with approximately 1 allergist per 133,000 people, some treatments are not easily accessible to atopic Canadians. Management approaches include allergen avoidance, pharmacotherapy including nasal corticosteroids and nasal or oral antihistamines, and allergen immunotherapy (AIT). Sublingual immunotherapy tablets (SLIT-T) has been demonstrated to significantly improve symptoms of AR and asthma,, and modify long-term tolerance to allergen exposure. Despite clinical efficacy, safety and convenience, SLIT-T is underutilized, partly related to the limited number of allergists in Canada. Given the home administration and safety of SLIT-T, non-allergists can play a vital role in the delivery of SLIT to their allergic patients.

**Methods:** The initial draft of a treatment algorithm for non-allergists was developed by primary care physician, Alan Kaplan. It was reviewed at an in-person meeting of an expert panel of four family physicians with a special interest in allergic disease and seven allergists. Consensus agreement resolved discrepancies.

**Results:** A focussed algorithm with treatment recommendations were developed for non-allergists to diagnose and implement SLIT-T in their allergic patients. This protocol requires accurate diagnosis based on history of timing of clinical symptoms, and focussed investigations, including skin testing where available, and serum-specific IgE. Patient selection was outlined with relevant cautions highlighted. With an appropriate history and investigations consistent with allergy to dust mite, tree, grass, or ragweed pollen, a rational approach to the initiation of SLIT-T by non-allergists is described. An annual review of clinical effectiveness was highlighted. Complex patients or non-responders should be referred for allergist assessment. Future validation is essential.

**Conclusions:** Safe and effective SLIT treatment for AR can be implemented by non-allergists with a focussed algorithm.

## 6 Nasal cytokine response following ragweed nasal allergen challenge using nasal sponges

### Sophia Linton^1,2^, Lubnaa Hossenbaccus^1,2^, Mya Gillberry^2^, Jenny Thiele^2,3^, Lisa M. Steacy^2^, Anne K. Ellis^1,2,3^

#### ^1^Department of Medicine, Queen’s University, Kingston, ON; ^2^Kingston General Health Research Institute-Allergy Research Unit, Kingston Health Science Centre-KGH Site, Kingston, ON; ^3^Department of Biomedical and Molecular Sciences, Queen’s University, Kingston, ON

##### **Correspondence:** Sophia Linton

*Allergy, Asthma & Clinical Immunology* 2024, **20(Suppl 1):**6

**Background:** The Allergic Rhinitis Clinical Investigator Collaborative’s nasal allergen challenge (NAC) protocol has been shown to detect cytokines reliably and reproducibly from nasal fluid. We sought to investigate using nasal sponges to collect and quantify nasal fluid cytokines in allergic and non-allergic participants.

**Methods:** Consenting participants were enrolled in the study outside the ragweed pollen season. During a screening visit, incremental concentrations of ragweed allergen were administered until each participant achieved a qualifying symptom score. For the subsequent NAC visit (21d–28d later), participants were challenged with a single allergen dose cumulative to the amount administered at the screening visit.

Nasal fluid from nasal sponges was collected at the screening visit (baseline) and the NAC visit (baseline, 6 h, 24 h, and 48 h post-NAC) and cryopreserved. The following analytes were measured using Luminex® xMAP® Technology: IL-1b, IL-4, IL-5, IL-6, IL-10, IL-13, IFN-g, MIP-1b, MCP-1, RANTES, TNF-a.

**Results:** 18 ragweed allergic and 12 non-allergic control participants completed both screening and NAC visits. All analytes were consistently detectable in the sponges, except for IFN-g and IL-1b. At the baseline screening visit, allergic and control participants had comparable concentrations of each analyte (Mann–Whitney test, P > 0.05). No significant differences were found for allergic and control participants when comparing the analyte concentrations between the baseline screening visit and the baseline NAC, respectively (Wilcoxon test, P > 0.05). Allergic participants had upregulated IL-4, IL-6, IL-10, MIP-1b, and TNF-a at 6 h post-NAC compared to controls. IL-5 was also upregulated at 24 h post-NAC compared to controls (2-way ANOVA, Dunn’s multiple comparison test, P > 0.05).

**Conclusions:** Ragweed allergic participants have an altered cytokine profile compared to non-allergic participants. Repeated sponges are a viable method that can be used to collect nasal fluid for cytokine analysis in an NAC.

## 7 Effect of dupilumab on lung function in various subgroups of children with uncontrolled asthma: long-term analysis

### Leonard B. Bacharier^1^, Theresa W. Guilbert^2^, Monika Gappa^3^, Sharon D. Dell^4,5^, Nikolaos G. Papadopoulos^6,7^, Antoine Deschildre^8^, Adnan Custovic^9^, Arman Altincatal^10^, Olivier Ledanois^11^, Rebecca Gall^12^, Harry Sacks^12^, Juby A. Jacob-Nara^13^, Yamo Deniz^12^, Paul J. Rowe^13^

#### ^1^Monroe Carell Jr Children’s Hospital at Vanderbilt University Medical Center, Nashville, TN, USA; ^2^Cincinnati Children’s Hospital and University of Cincinnati, Cincinnati, OH, USA; ^3^Children’s Hospital, Evangelisches Krankenhaus, Düsseldorf, Germany; ^4^BC Children’s Hospital, Vancouver, BC; ^5^University of British Columbia, Vancouver, BC; ^6^Royal Manchester Children’s Hospital, Manchester, United Kingdom; ^7^2nd Pediatric Clinic, University of Athens, Athens, Greece; ^8^CHU Lille, Lille, France; ^9^St Mary’s Campus Medical School, Imperial College, London, United Kingdom; ^10^Sanofi, Cambridge, MA, USA; ^11^Sanofi, Paris, France; ^12^Regeneron Pharmaceuticals Inc., Tarrytown, NY, USA; ^13^Sanofi, Bridgewater, NJ, USA

##### **Correspondence:** Sharon D. Dell

*Allergy, Asthma & Clinical Immunology* 2024, **20(Suppl 1):**7

**Background:** Long-term dupilumab use in EXCURSION (NCT03560466) sustained exacerbation reductions in 6–11-year-old patients with uncontrolled, moderate-to-severe type 2 asthma (eosinophils ≥ 150 cells/µL or FENO ≥ 20 ppb) who had completed VOYAGE (NCT02948959). The purpose of this post hoc analysis was to compare lung function between treatment arms across multiple subgroups.

**Methods:** Patients received add-on dupilumab 100/200 mg or placebo every 2 weeks (q2w) for 52 weeks in VOYAGE and dupilumab 100/200 mg q2w or 300 mg q4w for 52 weeks in EXCURSION. Endpoint was change from parent study baseline (PSBL) in percent predicted forced expiratory volume in 1 s (ppFEV_1_) by baseline ACQ-7-IA, asthma duration (dichotomized at median value 5.50), age at asthma onset, and age at dupilumab start for dupilumab/dupilumab groups and age at week 0 for placebo/dupilumab.

**Results:** At week 52, change from PSBL in ppFEV_1_ in dupilumab/dupilumab (n = 209) vs placebo/dupilumab (n = 106) was numerically greater with an ACQ-7-IA score ≥ 2 (LS mean difference [LSMD]: 1.55 [95% CI − 2.88–5.99] for ACQ-7-IA ≤ 2, 1.92 [− 3.87–7.72] for ACQ-7-IA ≥ 2; interaction *P* value: *P* = 0.76); shorter asthma duration (LSMD: 4.38 [− 0.88–9.64] for shorter, − 0.17 [− 4.85–4.51] for longer duration; *P* = 0.33); older age at onset (LSMD: 0.12 [− 5.10–5.34] for 0–2 years, 1.59 [− 5.56–8.74] for 3–5 years, 3.75 [− 2.86–10.35] for 6–11 years; *P* = 0.73); and younger age at dupilumab start (LSMD: 2.61 [− 3.14–8.36] for ≥ 6 to < 9 years, 1.52 [− 3.00–6.04] for ≥ 9 to < 12 years; *P* = 0.84). None of these differences between subgroups reached statistical significance.

**Conclusions:** Improved lung function response to dupilumab, while numerically different, did not differ statistically by baseline ACQ-7-IA score, asthma duration, age at onset, or dupilumab start.

## 8 Coexisting allergic rhinitis in patients with moderate-to-severe asthma: the RAPID registry study

### Anju T. Peters^1^, Andréanne Côté^2^, Xavier Muñoz^3^, Changming Xia^4^, Scott Nash^4^, Megan Hardin^5^, Lucía de Prado Gómez^6^, Harry Sacks^4^, Juby A. Jacob-Nara^7^, Yamo Deniz^4^, Paul J. Rowe^7^, Sven Sorensen^8^, Xavier Soler^4^

#### ^1^Northwestern University Feinberg School of Medicine, Chicago, IL, USA; ^2^Quebec Heart and Lung Institute – Laval University, Quebec, QC; ^3^Servei Pneumologia Hospital Vall d’Hebron, Barcelona, Spain; ^4^Regeneron Pharmaceuticals Inc., Tarrytown, NY, USA; ^5^Sanofi, Cambridge, MA, USA; ^6^Sanofi, Madrid, Spain; ^7^Sanofi, Bridgewater, NJ, USA; ^8^Sanofi, Mississauga, ON

##### **Correspondence:** Sven Sorensen

*Allergy, Asthma & Clinical Immunology* 2024, **20(Suppl 1):**8

**Background:** Coexisting type 2 inflammatory diseases such as allergic rhinitis (AR) are highly prevalent in patients with asthma. RAPID (NCT04287621), a global prospective registry, characterizes patients with asthma initiating dupilumab in real-world clinical practice. We evaluated the prevalence of AR in patients participating in RAPID and baseline characteristics in patients with and without coexisting AR.

**Methods:** RAPID enrolls patients aged ≥ 12 years initiating dupilumab for asthma (primary indication) according to country-specific prescribing information. Enrolled patients are assessed at baseline and Months 1, 3, and every 3 months thereafter for 3 years.

**Results:** At the time of this analysis, 205 patients were enrolled. 166 (81%) reported a history of AR, 165 (80%) reported ongoing AR, and 39 (19%) patients did not have AR. 115 (69%) patients with AR were female and mean age (SD) was 48.1 (17.5) years. In patients without AR, 19 (49%) were female, and mean (SD) age was 58.5 (14.2) years. Mean (SD) time since first asthma diagnosis was 21.9 (18.6) years in patients with AR and 16.8 (13.9) for those without. Mean (SD) asthma control (6-item Asthma Control Questionnaire [ACQ-6]) scores were 2.4 (1.20) in patients with AR and 2.2 (1.09) in those without. The mean (SD) Allergic Rhinitis Visual Analog Scale (AR-VAS) score was 48.0 (29.2) for patients with AR. With regards to medications used for AR, 63 (38%) patients used nasal steroids, 9 (5%) were on allergen immunotherapy, and 8 (5%) used ipratropium.

**Conclusions:** This analysis shows that coexisting AR was present in 80% of patients with asthma initiating dupilumab in real-world clinical practice, with more than a third (38%) using nasal steroids to control AR symptoms. Patients with coexisting AR were more likely to be female with an earlier onset of disease and had similar asthma control scores at baseline, regardless of coexisting AR.

## 9 The acute and chronic inflammatory effects of vaping in the airways and systemic circulation

### Reewa Kafle, Nami P. Shrestha, Yingqi Wu, Marc Duchesne, Harissios Vliagoftis

#### University of Alberta, Edmonton, AB

##### **Correspondence:** Reewa Kafle

*Allergy, Asthma & Clinical Immunology* 2024, **20(Suppl 1):**9

**Background:** Vaping rates are increasing, especially among young adults. However, little is known about the health effects of chemicals present in vaping liquid, independent of nicotine. We hypothesized that inhalation of chemicals released from vaping liquid induces acute and chronic inflammatory effects in the airways. Our long-term goal, after studying these effects in healthy individuals, is to investigate the pro-inflammatory effects of vaping in asthmatic subjects.

**Methods:** We recruited healthy habitual vapers (vaping at least twice a week for 3 or more months) and non-vapers. Subjects underwent one 30-min vaping challenge with nicotine-free vaping liquid (containing propylene glycol and vegetable glycerin) and a sham challenge with the vaping device turned off, one week apart. Blood and induced sputum were collected at baseline, then 2 and 24 h after each challenge. Inflammatory cells and activation markers in blood and sputum were analyzed by flow cytometry. We quantitated 10 chemokines in sputum and serum using a multiplex enzyme-linked immunosorbent assay (ELISA).

**Results:** We recruited 6 vapers, 3 of whom were former smokers, and 6 non-vapers. 50% of subjects in each group were women. The mean age of vapers and non-vapers was 24.33 and 30.83 years, respectively. Baseline pulmonary function and FeNO test results did not differ between habitual vapers and non-vapers. Blood monocytes and neutrophils were higher in vapers than non-vapers. Reactive oxygen species production and phagocytosis by blood monocytes and neutrophils decreased in vapers compared to non-vapers. After a vaping challenge, there was also a trend towards increased sputum monocyte and neutrophil counts compared to the sham challenge, but no change in chemokine levels.

**Conclusions:** Chronic vaping may increase monocytes and neutrophils in peripheral blood, but impair their function. Acute exposure to vaping may increase inflammatory cells in the airways. We are currently recruiting asthmatics to determine if their responses to vaping differ.

***Reewa Kafle—CSACI Summer Studentship Award winner***

## 10 Airway autoimmune responses driven by eosinophil extracellular traps (EETs) impact airway mucus

### Emily Volfson^1^, Aswathi Nair^1^, Alex Huynh^1^, Kiho Son^1^, Kayla Zhang^1^, Carmen Venegas-Garrido^1^, Katherine Radford^2^, Sarah Svenningsen^1^, Parameswaran Nair^1^, Manali Mukherjee^1^

#### ^1^McMaster University, Hamilton, ON; ^2^Firestone Institute for Respiratory Health, Hamilton, ON

##### **Correspondence:** Emily Volfson

*Allergy, Asthma & Clinical Immunology* 2024, **20(Suppl 1):**10

**Background:** Mucus plugging contributes to asthma severity and is often overlooked in clinical practice [1]. We recently reported IgG autoantibodies in sputum, and they contribute to asthma severity [2]. We hypothesize that rheological properties of mucus can be affected by autoantibody-triggered eosinophil extracellular traps (EET) that reduce its clearance potential leading to persistent plugging evident on chest Computed Tomography (CT).

**Methods:** Sputum samples from asthma patients with available chest CT radiology reports (n = 102, conducted clinically within 3 months) were examined for antinuclear antibodies (ANA) (HUMAN Diagnostics, [2]). Evidence of mucus was subjectively quantified based on the radiology report as significant, moderate, mild or none. Rheological properties of expectorated sputum (n = 15) were assessed using Rheomuco® rheometer (Rheonova, Gières, France). Effects of EETs on mucus rheology were assessed *ex-vivo* by adding increasing numbers (2.5–5 × 10^6^) of EETosing eosinophils (induced by autoantibodies) to expectorated sputum samples. Finally, 21-day old Calu-3 airway liquid interface (ALI) epithelial layers exposed to EETs for 48 h were examined for secreted mucin proteins (MUAC5 and MUC5B) (Novus Biologicals ELISA Kit).

**Results:** Patients with evidence of mucus plugging on CT had significantly higher sputum ANAs (p = 0.01). Sputum samples from patients with “significant” mucus plugging on CT had increased rheology parameters of critical stress, elastic modulus, and viscous modulus compared to those who had none/less (p < 0.05). Eosinophils (2.5 × 10^6^) derived from healthy donors (n = 3) were stimulated with autoantibodies to trigger EETosis *in-vitro*. EEETs increased the critical stress and viscosity of sputum. Finally, presence of EETs increased the release of MUCAC5 from ALI epithelial layers causing 1.65-fold increase in MUC5AC:MUC5B ratio compared to untreated wells (n = 2).

**Conclusions:** The study provides proof-of-concept evidence that sputum rheology can be affected by the presence of autoantibodies in an eosinophilic airway that leads to extracellular trap release and subsequent mucus production and plugging.

**References**

1. Svenningsen S, Nair P. Persistent Airway Plugs: A Call for Clinical Recognition and Novel Therapies. Am J Respir Crit Care Med. 2022 May 1;205(9):977–8.
2. Mukherjee M, Bulir DC, Radford K, Kjarsgaard M, Huang CM, Jacobsen EA, et al. Sputum autoantibodies in patients with severe eosinophilic asthma. J Allergy Clin Immunol. 2018 Apr;141(4):1269–79.

***Emily Volfson—CSACI Summer Studentship Award winner***

## Allied health

## 11 A cost comparison of allergen-friendly and non-allergen-friendly food products in Winnipeg, Manitoba

### Michael A. Golding^1,2^, Jennifer L. Protudjer^1,2,3^

#### ^1^Department of Pediatrics and Child Health, University of Manitoba, Winnipeg, MB; ^2^Children’s Hospital Research Institute of Manitoba, Winnipeg, MB; ^3^George & Fay Yee Centre for Healthcare Innovation, University of Manitoba, Winnipeg, MB

##### **Correspondence:** Michael A. Golding

*Allergy, Asthma & Clinical Immunology* 2024, **20(Suppl 1):**11

**Background:** Families managing food allergy spend more on food compared to families who do not manage food allergy. While it has been assumed that these cost differences reflect the higher price of allergen-friendly foods, the prices of allergen-friendly and non-allergen-friendly products have not been examined in the scientific literature to date. To fill this gap, we examined differences in food prices among allergen-friendly and non-allergen-friendly food products.

**Methods:** The prices of various food products were recorded from four supermarkets in Winnipeg, Manitoba between May 24th and May 29th, 2023. Two of the four stores were considered discount grocers, but all were part of large national chains. Products selected included those that would likely require an allergen-friendly substitute and were common in children’s diets. When possible, both name-brand and store-brand products were included in the cost comparisons. After compiling the list of items, we compared their prices using a series of independent samples t-tests.

**Results:** The final sample included 292 products, 122 of which were considered allergen-friendly. Of the 292 products, 103 were snacks and cereals, 77 were dairy-based items or dairy substitutes, 59 were convenience foods, and 53 were baking-related items. Results revealed the average price of the allergen-friendly products per 100 g/100 ml (*m* = 1.84, SD = 0.95) was significantly higher than the non-allergen-friendly products (*m* = 1.04, SD = 0.66, *p* < 0.0001). Significant price differences were also found across all food categories, except dairy and eggs (snacks & cereals: *m* = $2.14 vs. $1.02, p < 0.0001; convenience foods: *m* = $1.90 vs. $1.07, *p* < 0.0001; baking [i.e., flour, baking mixes, & chocolate chips): *m* = $1.59 vs. $0.83, *p* < 0.0001]; dairy & eggs: *m* = $1.54 vs. $1.18, *p* = 0.11).

**Conclusions:** The current study identified significant cost differences between allergen-friendly and non-allergen-friendly food products. These differences likely play a significant role in the higher food costs reported by families managing food allergy.

## 12 CFAE: the Canadian food allergy educator program

### Jennifer L. Protudjer^1,2,3^, Douglas P. Mack^4^, Lori Connors^5^, Jasmin Lidington^6^, Harold Kim^7^

#### ^1^University of Manitoba, Winnipeg, MB; ^2^Children’s Hospital Research Institute of Manitoba, Winnipeg, MB; ^3^George and Fay Yee Centre for Healthcare Innovation, Winnipeg, MB; ^4^McMaster University, Hamilton, ON; ^5^Dalhousie University, Halifax, NS; ^6^Canadian Society of Allergy and Clinical Immunology, Ottawa, ON; ^7^University of Western Ontario, Kitchener, ON

##### **Correspondence:** Jennifer L. Protudjer

*Allergy, Asthma & Clinical Immunology* 2024, **20(Suppl 1):**12

**Background:** Allied health training programs in Canada have minimal curricula dedicated to food allergy. As a result, allied health professionals report limited knowledge, and a desire for more training in food allergy. The aim of this integrated knowledge translation project is to create and evaluate a food allergy educator program for Canadian allied health professionals.

**Methods:** To achieve this aim, our specific objectives are to develop, implement and evaluate two virtual courses, the content of which will be delivered by world leaders in food allergy. First, an 8-week foundational course on food allergy, offered twice yearly, will be open to all allied health professionals in Canada. This foundational course will cover topics ranging from food allergy epidemiology, diagnosis and management, to the psychosocial burden of food allergy. The first intake for this course is planned for September, 2023, and is a prerequisite for the second course. Second, a 12-week advanced practice course on food allergy will be offered once yearly, for a limited number of competitively-selected allied health professionals in Canada, and is intended for those working in food allergy. Formal evaluations, of students and of the program itself, will be included in both the foundational course and the advanced practice course. As an educational course, the University of Manitoba Research Ethics Board deemed research ethics board approval unnecessary subsequent to review of the course proposal and overview.

**Results:** As of early July 2023, website development is nearly completed, speakers and moderators for the Fall 2023 Foundational Course have been confirmed, and pre- and post-examination questions are being solicited from all speakers.

**Conclusions:** Our long-term vision is to close this knowledge gap, by developing, implementing, and evaluating a needs-informed and evidence-based food allergy educator program for dietitians in Canada.

## 13 Food allergy and food insecurity estimates of prevalence by operationalisation: a scoping review

### Elizabeth Magaji^1,3^, Rebecca Kim^2,3^, Michael A. Golding^1,3^, Jennifer L. Protudjer^1,3,4^

#### ^1^University of Manitoba, Winnipeg, MB; ^2^Western University, London, ON; ^3^Children’s Hospital Research Institute of Manitoba, Winnipeg, MB; ^4^Institute of Environmental Medicine, Stockholm, Sweden

##### **Correspondence:** Elizabeth Magaji

*Allergy, Asthma & Clinical Immunology* 2024, **20(Suppl 1):**13

**Background:** Compared to families not managing food allergy, families managing food allergy face higher food costs and thus may be at higher risk for food insecurity. Our scoping review aims to examine the prevalence of food insecurity within families managing food allergy with consideration to demographic factors and how food insecurity is operationalized.

**Methods:** The current scoping review follows Arksey and O’Malley’s framework, the Preferred Reporting Items for Systematic Reviews and Meta-Analyses Extension for Scoping Reviews review guidelines and uses search terms developed by a health sciences librarian. Databases searched included MedLine, EmBase, Scopus, and Food Sciences and Technology Abstracts as well as the grey literature. Original articles, regardless of date of publication, focused on food allergy and food insecurity, published in English or French, were eligible for review. All articles will be screened by two reviewers blinded to each other’s initial decisions. Disagreements between reviewers will be resolved through discussion or by a third reviewer if consensus cannot be reached.

**Results:** Peer-reviewed literature was searched on June 20, 2023, yielding 3748 unique articles. We are currently in the process of searching the grey literature. Afterwards, all articles will be screened. Once the final sample of articles is established, we will extract data from each article pertaining to the definition of food insecurity used, rates of food insecurity, and the influence of demographic factors. This information will ultimately be synthesized using a narrative synthesis.

**Conclusions:** Families affected by food allergy may face an elevated risk of experiencing food insecurity. Estimating the rates of food insecurity among families navigating food allergy will help identify potential discrepancies in food insecurity between families with and without food allergy while contributing to further policy guidance.

Thank you to Carol Cooke of the University of Manitoba Neil John Maclean Health Sciences Library for guidance in developing search terms.

## 14 Concern among Canadian health professionals with difficulty maintaining regular ingestion of allergenic foods once introduced to infants

### Brock A. Williams^1,2^, Stephanie C. Erdle^1,2^, Andrea Grantham^3^, Jennifer L. Protudjer^4,5,6^, Elissa Abrams^4^, Rachel G. Khadaroo^7^, Lianne Soller^1,2^, Edmond S. Chan^1,2^

#### ^1^University of British Columbia, Vancouver, BC; ^2^BC Children’s Hospital, Vancouver, BC; ^3^Canadian Nutrition Society, Ottawa, ON; ^4^University of Manitoba, Winnipeg, MB; ^5^Children’s Hospital Research Institute of Manitoba, Winnipeg, MB; ^6^Institute of Environmental Medicine, Stockholm, Sweden; ^7^University of Alberta, Edmonton, AB

##### **Correspondence:** Stephanie C. Erdle

*Allergy, Asthma & Clinical Immunology* 2024, **20(Suppl 1):**14

**Background:** In 2019 and 2021, the Canadian Paediatric Society/Canadian Society of Allergy and Clinical Immunology updated guidance to recommend that all commonly allergenic foods are introduced into the diet of high-risk infants ~ 6, but not before 4, months of age. To date, there are no published data on knowledge gaps among Canadian health professionals regarding this recent guidance.

**Objective:** To determine health professional knowledge gaps and concerns for infant feeding guidance, before and after targeted knowledge translation activities.

**Methods:** We presented two webinars via the Canadian Nutrition Society on the topic of advances in food allergy prevention and management (February & March 2023). Webinar participants were invited to complete an anonymized survey (UBC ethics-exempt) after the second webinar assessing familiarity and knowledge of current guidance before and after the webinars. McNemar’s and Wilcoxon matched-pairs signed-rank tests were applied to determine differences in intake frequency responses and Likert scale responses (one = unfamiliar, five = very familiar) pre- and post-webinars, respectively.

**Results:** In total, 76 individuals (71% dietitians, 8% nurses, 7% scientists, 3% physicians, 11% other) participated. Participants rated familiarity with the concept of “interrupted”/infrequent ingestion of allergenic foods potentially increasing risk of food allergy a median (IQR) score of 2 (1, 4) pre-webinars; this increased to 4 (4, 5) post-webinars (n = 64; p < 0.05). Pre-webinars, 22% (n = 17/75) of individuals believed that if an infant is given cow’s milk formula as a supplement to human milk in the first month of life, it should subsequently be given ≥ 2–3 times/week to maintain tolerance; this increased to 87% (n = 55/63) post-webinars (p < 0.05). The most reported concern among attendees was difficulty maintaining regular ingestion of commonly allergenic foods once introduced (49%); other concerns comprised ≤ 15% each.

**Conclusions:** Given the most reported concern by Canadian health professionals is difficulty maintaining regular ingestion of allergenic foods once introduced, novel strategies for increasing regularity of ingestion are needed.

## 15 Asthma-related Emergency Department visits and hospitalizations at the Children’s Hospital of Winnipeg before and during the COVID-19 pandemic

### Jo-Anne St-VIncent^1^, Shauna Filuk^1^, Lenore Johnstone^1^, Lenore Johnstone^1^, Nancy Ross^1^, Janice Skoleski^1^, Loewen Keeley^2^, Shannon Deane^2^, Elinor Simons^3^

#### ^1^The Children’s Allergy and Asthma Education Centre, Winnipeg, MB; ^2^Section of Allergy and Immunology, Department of Pediatrics and Child Health, University of Manitoba, Winnipeg, MB; ^3^Children’s Hospital Research Institute of Manitoba, Winnipeg, MB

##### **Correspondence:** Jo-Anne St-Vincent

*Allergy, Asthma & Clinical Immunology* 2024, **20(Suppl 1):**15

**Background:** Nurse Educators at the Children’s Allergy & Asthma Education Centre (CAAEC) have monitored asthma-related health care use at the Children’s Hospital of Winnipeg before and during the COVID-19 Pandemic, particularly the yearly September epidemic of asthma exacerbations.

**Methods:** As part of ongoing monitoring of health care use, Shared Health provided statistics regarding Children’s Hospital Emergency Department (ED) visits and hospitalizations for asthma from 2019–2023. Comparisons between 2019 and 2020–2023 were made for ED visits for asthma, hospitalizations for asthma, and asthma admissions to the Pediatric Intensive Care Unit (PICU) directly from the ED or as transfers from the ward. We also noted dates of changes in public health measures from 2019–2023.

**Results:** Compared with ED visits for asthma in September 2019 (n = 180), we observed a 79% decrease in September 2020 (n = 38), when public health measures were most restrictive, a 39% decrease in September 2021 (n = 110), when mask mandates remained in effect in schools, and a 67% increase in September 2022 (n = 300) when most public health restrictions were removed.

Compared with hospitalizations for asthma in September 2019 (n = 3), we observed no change in 2020 (n = 3), a 300% increase in 2021 (n = 12), and a 1100% increase in 2022 (n = 36). In 2022 versus 2019, PICU asthma admissions directly from the ED increased by 175% and from the ward increased by 263%. The increases in ED visits and hospitalizations for asthma began in April 2022 and persisted until December 2022.

**Conclusions:** The Children’s Hospital of Winnipeg saw a dramatic increase in ED visits and hospitalizations for asthma in 2022, peaking in September. Yearly changes in the September epidemic during the Pandemic coincided with changes in public health restrictions. The dramatic increase in hospitalizations and PICU admissions in 2022 demonstrated increased severity and frequency of asthma exacerbations after the lifting of public health measures.

## Food allergy/anaphylaxis

## 16 Real world experience: a retrospective pediatric chart review to determine why patients discontinue oral immunotherapy

### Amy Plessis^4^, Scott B. Cameron^1,2,3^, Victoria E. Cook^1,2,3^

#### ^1^Division of Allergy and Immunology, Department of Pediatrics, University of British Columbia, Vancouver, BC; ^2^British Columbia Children’s Hospital Research Institute and the University of British Columbia, Vancouver, BC; ^3^Community Allergy Clinic, Victoria, BC; ^4^Department of Pediatrics, Faculty of Medicine, University of British Columbia Island Medical Program, Victoria, BC

##### **Correspondence:** Amy Plessis

*Allergy, Asthma & Clinical Immunology* 2024, **20(Suppl 1):**16

**Background:** Oral immunotherapy (OIT) is an increasingly prevalent management strategy for IgE-mediated food allergy. Despite promising results with OIT there is still a lack of information surrounding reasons for discontinuation of OIT. The primary reason stated in the literature for discontinuation is adverse gastrointestinal effects. Social factors contributing to OIT discontinuation have not been well reported. We hypothesize that for many families, the social considerations are significant contributors to discontinuation.

**Methods:** We report a retrospective chart review of 50 patients in community pediatric allergy practices who discontinued OIT between October 1 2017–October 27 2022. The reasons for discontinuation were identified and classified into five main categories: unsafe choices, anxiety, adverse effects of OIT, uncontrolled comorbidity and social factors. Categories were not exclusive.

**Results:** Data were available on 50 patients, aged 10 months to 18 years and 2 months. Overall rate of discontinuation was 9.8% of which 40 patients (82%) discontinued during buildup phase and 9 patients (18%) discontinued during maintenance. 30 patients (60%) had multiple reasons for discontinuing OIT. The most common reason for discontinuation was social factors, which were identified in 29 patients (58%). 22 patients (44%) discontinued OIT due to adverse effects, gastrointestinal symptoms being the most prevalent. 13 patients (26%) were identified as having anaphylaxis contribute to discontinuation and 17 patients (34%) had anxiety leading to discontinuation.

**Conclusions:** Our data highlights the importance that social factors and anxiety play in the success of OIT completion which to the best of our knowledge is underrepresented in the current literature. Our data supports that when selecting patients who are good candidates for OIT we need to consider not only the patient’s medical history, but also their social history and support networks to optimize the successful completion of OIT.

## 17 The need for in-hospital management following use of an epinephrine auto-injector in pre-hospital settings

### Lauren Perlman^1^, Sofianne Gabrielli^1^, Ann E. Clarke^2^, Luca Delli Colli^1^, Marina Delli Colli^1^, Judy Morris^3^, Jocelyn Gravel^4^, Rodrick Lim^5^, Edmond S. Chan^6^, Ran D. Goldman^7^, Andrew O’Keefe^8^, Jennifer Gerdts^9^, Derek K. Chu^10^, Julia Upton^11^, Elana Hochstadter^12^, Jocelyn Moisan^13^, Adam Bretholz^14^, Christine McCusker^1^, Xun Zhang^15^, Jennifer L. Protudjer^16,17^, Elissa M. Abrams^18^, Elinor Simons^18^, Moshe Ben-Shoshan^1^

#### ^1^Division of Pediatric Allergy, Clinical Immunology and Dermatology, Montreal Children’s Hospital, McGill University Health Centre, Montreal, QC; ^2^Division of Rheumatology, Department of Medicine, Cumming School of Medicine, University of Calgary, Calgary, AB; ^3^Department of Emergency Medicine, Sacré-Coeur Hôpital, Montreal, QC; ^4^Department of Pediatric Emergency Medicine, Centre Hospitalier Universitaire Sainte-Justine, Université de Montréal, Montreal, QC; ^5^Division of Pediatric Emergency Medicine, Department of Pediatrics, Schulich School of Medicine & Dentistry, University of Western Ontario, London, ON; ^6^Division of Allergy, Department of Pediatrics, BC Children’s Hospital, University of British Columbia, Vancouver, BC; ^7^Division of Clinical Pharmacology and Emergency Medicine, Department of Pediatrics, BC Children’s Hospital, University of British Columbia, and the BC Children’s Research Institute, Vancouver, BC; ^8^Department of Pediatrics, Faculty of Medicine, Memorial University, St John’s, NL; ^9^Executive Director, Food Allergy Canada, Toronto, ON; ^10^Division of Clinical Immunology & Allergy, Department of Medicine, and Department of Health Research Methods, Evidence & Impact, McMaster University, Hamilton, ON; ^11^Division of Immunology and Allergy, Department of Pediatrics, The Hospital for Sick Children, Department of Paediatrics, University of Toronto, Toronto, ON; ^12^Department of Pediatric Emergency Medicine, The Hospital for Sick Children, University of Toronto, Toronto, ON; ^13^Regional Medical Director of Emergency Medical Services of Outaouais, Outaouais, QC; ^14^Division of Pediatric Emergency Medicine, Department of Pediatrics, Montreal Children’s Hospital, Montreal, QC; ^15^Centre for Outcomes Research and Evaluation, Research Institute of McGill University Health Centre, Montreal, QC; ^16^Department of Pediatrics and Child Health, Rady Faculty of Health Sciences, University of Manitoba; and, Children’s Hospital Research Institute of Manitoba, Winnipeg, MB; ^17^Institute of Environmental Medicine, Karolinska Institutet, Stockholm, Sweden; ^18^Section of Allergy and Clinical Immunology, Department of Pediatrics and Child Health, Rady Faculty of Health Sciences, University of Manitoba and Children’s Hospital Research Institute of Manitoba, Winnipeg, MB

##### **Correspondence:** Lauren Perlman

*Allergy, Asthma & Clinical Immunology* 2024, **20(Suppl 1):**17

**Background:** Prompt epinephrine administration is the first-line treatment for anaphylaxis, with emergency department (ED) observation following use. However, data supporting transfer to the ED in all cases using epinephrine are sparse, and some guidance no longer recommends ED visits [1, 2]. We aimed to assess the need for additional ED treatment in children with anaphylaxis using an epinephrine auto-injector in the pre-hospital setting.

**Methods:** Children < 18 years with food-induced anaphylaxis who received at least one dose of pre-hospital epinephrine were enrolled in the Cross-Canada Anaphylaxis REgistry (C-CARE) from seven hospitals. A standardized questionnaire collected information on symptoms, severity, triggers, comorbidities, and management. Multivariable logistic regression was performed to identify factors associated with additional ED management.

**Results:** From 2011 to 2023, 887 children with anaphylaxis with known food allergies used epinephrine in the pre-hospital setting. The mean age at reaction was 7.9 years (SD 5.3), 550 (62.%) were male and 59 (6.7%) of reactions were severe (cyanosis, respiratory arrest, hypotension, circulatory collapse, dysrhythmia, bowel incontinence, cardiac arrest, serious bradycardia, confusion, and unconsciousness [3]). The main triggers were peanuts (n = 229; 25.8%) and tree nuts (n = 146; 16.5%), and 18.0% had known asthma. In the ED, 172 patients (19.4%) received additional doses of epinephrine. Nearly all (158/172; 91.9%) received one dose, whereas few received 2 doses, or 3 or more (n = 9 and n = 5, respectively). Severe reactions (adjusted odds ratio [aOR] 1.19; 95% confidence interval [CI] 1.08–1.31) and reactions to tree nuts (aOR 1.12; 95% CI 1.04–1.20) were associated with increased odds of an additional dose of epinephrine in hospital, while severe reactions were associated with 2+ additional doses (OR 1.08; 95% CI 1.04–1.11). 2.5% of patients required IV fluids and 1.6% required admission.

**Conclusions:** Arrival at the ED for additional treatment following epinephrine auto-injector use should be considered mainly for children with severe reactions and reactions to tree nuts.

**References**

1. Casale TB, Wang J, Nowak-Wegrzyn A. Acute At Home Management of Anaphylaxis During the Covid-19 Pandemic. J Allergy Clin Immunol Pr. 2020;8:1795–7.
2. Greenhawt M, Lieberman JA, Dribin TE, Shaker MS, Spergel J. Retire the advice to send patients to the emergency department after epinephrine use for observation. Ann Allergy Asthma Immunol. 2023;130:697–8.
3. Gabrielli S, Clarke A, Morris J, Eisman H, Gravel J, Enarson P, et al. Evaluation of Prehospital Management in a Canadian Emergency Department Anaphylaxis Cohort. J Allergy Clin Immunol Pract. 2019;7(7):2232–8 e3.

## 18 The panopticon of mothers of teenagers with life-threatening food allergies: vigilance, watchfulness, and surveillance—offering hope for the future

### Karen A. Dobbin-Williams

#### Memorial University of Newfoundland, St. John’s, NL

##### **Correspondence:** Karen A. Dobbin-Williams

*Allergy, Asthma & Clinical Immunology* 2024, **20(Suppl 1):**18

**Background:** Food allergy affects close to 500,000 individuals in Canada [1]. Globally, the number of individuals with food allergy has continued to rise with no cure for those whose allergies are life-threatening [2].

There is much published knowledge about how teenaged children feel about their life-threatening food allergies with reports of bullying, feeling excluded or like a burden to others (3–22).

Far less is known about mothers’ experiences with caring for a teenaged child with anaphylactic food allergies. It is known that mothers report worries about life transitions, carrying of epinephrine auto-injectors, peer pressure, and avoidance of the offending allergen, to name a few (23–27).

**Methods:** In this qualitative philosophical hermeneutic inquiry study, mothers were interviewed about this experience as their children were now teenagers who lived with life-threatening food allergies. This exploration uncovered new understandings about this experience that have not previously been published. Influenced by the philosophical hermeneutics of Hans-Georg Gadamer; transcription of interviews, in-depth engagement with the transcriptions, and analysis of interviews in hermeneutic research tradition occurred (28).

**Results:** Findings revealed that this experience encompasses vigilance, watchfulness, and surveillance, advocacy, looking toward the future while looking backwards over life already lived, and allergies taking a backseat to other worries during the teenaged years.

**Conclusions:** Vigilance, watchfulness, and surveillance are a strong component of the experience of mothers of teenagers with life-threatening food allergies, but allergies do take a backseat to other worries as the teenager grows. Recommendations are offered on how nurses and other healthcare professionals, and mothers with experience themselves, can support and provide hope for families with individuals with newly diagnosed anaphylactic food allergies.

**References**

1. Clarke et al. (2020). Temporal trends in food allergy prevalence in Canada. The Journal of Allergy and Clinical Immunology: in Practice, 8(4), 1428–1430.E5. 10.1016/j.jaip.2019.10.021
2. Loh, W. and Tang, M. (2018). The epidemiology of food allergy in the global context. International Journal of Environmental Research and Public Health, 15, 2043–2050. 10.3390/ijerph15092043
3. Akeson, N., Worth, A., & Sheikh, A. (2007). The psychosocial impact of anaphylaxis on young people and their parents. Clinical and Experimental Allergy, 37, 1213–1220. 10.1111/j.1365-2222.2007.02758.x
4. DunnGalvin, A., Gaffney, A., & Hourihane, J. (2009). Developmental pathways in food allergies: A new theoretical framework. Allergy, 64, 560–568. 10.1111/j.1398-9995.2008.01862.x
5. Fenton, N., Elliott, S., Cicutto, L., Clarke, A., Haranda, L., & McPhee, E. (2011). Illustrating risk: Anaphylaxis through the eyes of the food-allergic child. Risk Analysis, 31(1), 171–183. 10.1111/j.1539-6924.2010.01488.x
6. Fox, J., & Warner, C. (2017). Food allergy and social anxiety in a community sample of adolescents. Children’s Health Care, 46(1), 93–107. 10.1080/02739615.2015.1124773
7. Gallagher, M., Worth, A., Cunningham-Burley, S., & Sheikh, A. (2011). Epinephrine auto-injector use in adolescents at risk of anaphylaxis: A qualitative study in Scotland, UK. Clinical and Experimental Allergy, 41, 869–877. 10.1111/j.1365-2222.2011.03743.x
8. Gallagher, M., Worth, A., Cunningham-Burley, S., & Sheikh, A. (2012). Strategies for living with the risk of anaphylaxis in adolescence: Qualitative study of young people and their parents. Primary Care Respiratory Journal, 21(4), 392–397. 10.4104/pcrj.2012.00072
9. Herbert, L., Lin, A., Matsui, E., Wood, R., & Sharma, H. (2016). Development of a tool to measure youths’ food allergy management facilitators and barriers. Journal of Pediatric Psychology, 363–372. 10.1093/jpepsy/jsv099
10. Lieberman, J., Weiss, C., Furlong, T., & Sicherer, S. (2010). Bullying of pediatric patients with food allergy. Journal of Allergy and Clinical Immunology, 125(Suppl. 1)., AB213.
11. Lyons, A., & Forde, E. (2004). Food allergy in young adults: Perceptions and psychological effects. Journal of Health Psychology, 9, 497–504. 10.1177/1359105304044032
12. MacKenzie, H., Roberts, G., Van Laar, D., & Dean, T. (2010). Teenagers’ experiences of living with food hypersensitivity: A qualitative study. Pediatric Allergy and Immunology, 21(4p1), 595–602. 10.1111/j.1399-3038.2009.00938.x
13. Marklund, B., Wilde-Larsson, B., Ahlstedt, S., & Nordstrom, G. (2007). Adolescents’ experiences of being food hypersensitive: A qualitative study. BMC Nursing, 6(8). 10.1186/1472-6955-6-8
14. Monks, H., Gowland, M., MacKenzie, H., Erlewyn-Lajeunesse, M., King, R., Lucas, J., & Roberts, G. (2010). How do teenagers manage their food allergies? Clinical and Experimental Allergy, 40, 1533–1540. 10.1111/j.1365-2222.2010.03586.x
15. Protjudjer, J., Jansson, S., Middelveld, R., Ostblom, E., Dahlen, S., Arnlind, M.,…Ahlstedt, S. (2016). Impaired health-related quality of life in adolescents with allergy to staple foods. Clinical and Translational Allergy, 6(37), 1–9. 10.1186/s13601-016-0128-5
16. Saleh-Langenberg, J., Flokstra-de Blok, B., Goossens, N., Kemna, J., van der Velde, J., & Dubois, A. (2016). The compliance and burden of treatment with the epinephrine auto-injector in food-allergic adolescents. Pediatric Allergy and Immunology, 27, 28–34. 10.1111/pai.12458
17. Sampson, M., Munoz-Furlong, A., & Sicherer, S. (2006). Risk-taking and coping strategies of adolescents and young adults with food allergy. The Journal of Allergy and Clinical Immunology, 117(6), 1440–1445. 10.1016/j.jaci.2006.03.009
18. Shemesh, E., Annunziato, R., Ambrose, M., Ravid, N., Mullarkey, C., Rubes, M.,…Sicherer, S. (2013). Child and parental reports of bullying in a consecutive sample of children with food allergy. Pediatrics, 131, e10-e17. 10.1542/peds.2012-1180
19. Sommer, I., MacKenzie, H., Venter, C., & Dean, T. (2014). An exploratory investigation of food choice behavior of teenagers with and without food allergies. Annals of Allergy and Immunology, 112, 446–452. 10.1016/j.anai.2014.02.009
20. Stensgaard, A., Bindslev-Jensen, C., & Nielsen, D. (2016). Peanut allergy as a family project: Social relations and transitions in adolescence. Journal of Clinical Nursing, 1–11. 10.1111/jocn.13696
21. Stjerna, M. (2015). Food, risk and place: Agency and negotiations of young people with food allergy. Sociology of Health and Illness, 37(2), 284–297. 10.1111/1467-9566.12215
22. Warren, C., Dyer, A., Otto, A., Smith, B., Kauke, K., Dinaker, C., & Gupta, R. (2017). Food allergy-related risk-taking and management behaviors among adolescents and young adults. Journal of Allergy and Clinical Immunology: In Practice, 5, 381–390. 10.1016/j.jaip.2016.12.012
23. Akeson, N., Worth, A., & Sheikh, A. (2007). The psychosocial impact of anaphylaxis on young people and their parents. Clinical and Experimental Allergy, 37, 1213–1220. 10.1111/j.1365-2222.2007.02758.x
24. Gallagher, M., Worth, A., Cunningham-Burley, S., & Sheikh, A. (2011). Epinephrine auto-injector use in adolescents at risk of anaphylaxis: A qualitative study in Scotland, UK. Clinical and Experimental Allergy, 41, 869–877. 10.1111/j.1365-2222.2011.03743.x
25. Gallagher, M., Worth, A., Cunningham-Burley, S., & Sheikh, A. (2012). Strategies for living with the risk of anaphylaxis in adolescence: Qualitative study of young people and their parents. Primary Care Respiratory Journal, 21(4), 392–397. 10.4104/pcrj.2012.00072
26. Mandell, D., Curtis, R., Gold, M., & Hardie, S. (2005). Anaphylaxis: How do you live with it? Health and Social Work, 30(4), 325–335. 10.1093/hsw/30.4.325
27. Stensgaard, A., Bindslev-Jensen, C., & Nielsen, D. (2016). Peanut allergy as a family project: Social relations and transitions in adolescence. Journal of Clinical Nursing, 1–11. 10.1111/jocn.13696
28. Moules, N.J., McCaffrey, G., Field, J.C., & Laing, C.M. (2015). Conducting hermeneutic research: From philosophy to practice. New York, NY: Peter Lang.

## 19 Real-world effectiveness analysis of preschool tree nut oral immunotherapy

### Simonne L. Horwitz^1^, Lianne Soller^2^, Victoria E. Cook^2,3^, Scott B. Cameron^2,3^, Joanne Yeung^2,4^, Sandeep Kapur^5,6^, Mary McHenry^5,6^, Edmond S. Chan^2^, Raymond Mak^2^, Gregory A. Rex^5,6^, Tiffany Wong^2^, Stephanie C. Erdle^2^

#### ^1^Department of Pediatrics, The Hospital for Sick Children, University of Toronto, Toronto, ON; ^2^Division of Allergy, Department of Pediatrics, University of British Columbia, Vancouver, BC; ^3^Community Allergy Clinic, Victoria, BC; ^4^Vancouver Kids Allergy, Vancouver, BC; ^5^Division of Allergy, Department of Pediatrics, Dalhousie University/IWK Health Centre, Halifax, NS; ^6^Halifax Allergy and Asthma Associates, Halifax, NS

##### **Correspondence:** Simonne L. Horwitz

*Allergy, Asthma & Clinical Immunology* 2024, **20(Suppl 1):**19

**Background:** We previously described the safety of preschool tree nut oral immunotherapy (TN-OIT) and both the safety and effectiveness of preschool peanut oral immunotherapy (P-OIT) in a real-world setting. We sought to determine the effectiveness of preschool TN-OIT after at least one year of daily maintenance TN-OIT.

**Methods:** As part of a Canada-wide quality improvement project, preschool-age children with (1) an objective reaction to tree nut and a positive skin prick test or specific IgE level of 0.35 kU/L or greater or (2) specific IgE level of 5kU/L or greater received a follow-up OFC to cumulative 4000 mg tree nut protein after at least one year of 300 mg maintenance TN-OIT. Each patient completed TN-OIT and follow-up OFC to at least one tree nut (cashew/pistachio, walnut/pecan, hazelnut, almond, or macadamia nut), with some patients completing TN-OIT to more than one tree nut simultaneously, with follow-up OFCs for each. Effectiveness of desensitization was defined as proportion of patients with negative follow-up OFC. Symptoms were classified using the modified World Allergy Organization Subcutaneous Immunotherapy Systemic Reaction Grading System (1, mildest; 5, fatal).

**Results:** 47 patients completed TN-OIT to at least one tree nut and subsequently underwent 54 follow-up OFCs; 45/54 (83.3%) had a successful OFC to 4000 mg protein and 53/54 (98.1%) tolerated a cumulative dose of greater than or equal to 1000 mg protein. Of the 9 reactions, 6 (66.7%) were grade 1 reactions and 3 (33.3%) were grade 2 reactions, with no grade 3–5 reactions. All grade 2 reactions were to cashew. No patients required epinephrine during the follow-up OFC.

**Conclusions:** Our preliminary data demonstrate that real-world preschool TN-OIT is effective, with comparable outcomes to previously published P-OIT data. For those who reacted, the vast majority had their threshold increased sufficiently to protect against accidental exposures. TN-OIT should be considered for preschool-age children as an alternative to strict avoidance.

## 20 SORT: Safety of OIT in a randomized trial for peanut and milk

### Adnan Al Ali^1^, Danbing Ke^1^, Liane Beaudette^1^, Julia Upton^2^, Eyal Grunebaum^2^, Ciriaco Piccirillo^3^, Edmond S. Chan^4^, Xun Zhang^1^, Casey Cohen^1^, Christine McCusker^1^, Bruce Mazer^1^, Abigail Brodovitch^5^, Moshe Ben-Shoshan^1^

#### ^1^Division of Allergy and Clinical Immunology, Department of Pediatrics, Montreal Children’s Hospital, McGill University Health Centre, Montreal, QC, Canada., Montreal, QC; ^2^Division of Immunology and Allergy, Department of Pediatrics, The Hospital for Sick Children, Department of Paediatrics, University of Toronto, Toronto, ON, Canada., Toronto, ON; ^3^Department of Microbiology and Immunology, McGill University Health Center, Montreal, Quebec, Canada., Montreal, QC; ^4^Division of Allergy, Department of Pediatrics, BC Children’s Hospital, University of British Columbia, Vancouver, British Columbia, Canada., Vancouver, BC; ^5^Montreal Children’s Hospital, McGill University Health Centre, Montreal, QC, Canada., Montreal, QC

##### **Correspondence:** Adnan Al Ali

*Allergy, Asthma & Clinical Immunology* 2024, **20(Suppl 1):**20

**Background:** Oral immunotherapy (OIT) has emerged as a promising approach. However, concerns regarding safety and the lack of standardized protocols have impeded the widespread adoption of OIT.

**Methods:** This study aims to address the gaps related to OIT for milk and peanut protocols by focusing on developing safer yet effective protocols. Group A followed the standard protocol with a dose of 200 ml of milk and 300 mg of peanuts. Group B employed lower target doses of 50 ml milk and 30 mg peanuts. Group C incorporated processed forms of the allergens, such as daily baked goods with milk and peanut snacks. The participants were recruited from allergy clinics at the Montreal Children’s Hospital. Allergic reactions were classified as mild allergic reaction (AR), moderate AR; severe AR, anaphylactic allergic reaction (AAR); severe AAR. Anaphylaxis was defined by involving at least two organ systems/hypotension [1]. The severe anaphylactic reaction was defined by cyanosis, hypoxia, respiratory arrest, hypotension, dysrhythmia, confusion, or loss of consciousness.

**Results:** There were 16 children recruited for milk and 16 for peanuts in each group A, B, and C. The median age was 7.5 years (IQR: 3.75). Among children undergoing peanut OIT, 6 had AR, and 6 had AAR. Among children undergoing milk OIT, 10 had AR, and 10 had AAR. For milk, OIT use of a protocol with baked goods was associated with a lower risk of severe allergic reactions (aOR, 7.76; CI, 1.33–45.26). The male sex (8 males) was associated with less moderate/severe anaphylactic reactions (aOR, 0.08; CI, 0.01–0.87).

**Conclusions:** Baked milk may offer a safer strategy for OIT. However, the limited sample size precludes definitive conclusions, and its relative efficacy needs to be studied. Further research with larger sample sizes is necessary to compare appropriately between the three protocols.

**Reference**

1. Panel NS. Guidelines for the diagnosis and management of food allergy in the United States: report of the NIAID-sponsored expert panel. Journal of Allergy and Clinical Immunology. 2010 Dec 1;126(6):S1–58. 10.1016/j.jaci.2010.10.007.

## 21 What does not kill you makes you less sensitive: serological evaluation of a subset of patients undergoing low vs standard maintenance dose peanut oral immunotherapy

### Diana Toscano Rivero^1^, Casey G. Cohen^1^, Wei Zhao^1^, Eisha A. Ahmed^1^, Danbing Ke^2^, Liane Beaudette^2^, Duncan Lejtenyi^2^, Thomas Eiwegger^3^, Moshe Ben-Shoshan^2^, Julia Upton^3^, Bruce D. Mazer^1,2^

#### ^1^Research Institute of the McGill University Health Centre, Montreal, QC; ^2^Division of Allergy and Clinical Immunology, Department of Pediatrics, Montreal Children’s Hospital, Montreal, QC; ^3^Division of Immunology and Allergy, Department of Pediatrics, Hospital for Sick Children, University of Toronto, Toronto, ON

##### **Correspondence:** Diana Toscano Rivero

*Allergy, Asthma & Clinical Immunology* 2024, **20(Suppl 1):**21

**Background:** Peanut oral immunotherapy (OIT) lowers the risk of allergic reactions by gradually exposing allergic individuals to increasing amounts of peanuts until a maintenance dose of 300 mg is reached. However, this process is hindered by frequent dose-related adverse reactions, which often result in participant withdrawals. Data on immunologic parameters linked to maintenance doses below 300 mg are limited.

**Methods:** A subset of 29 peanut-allergic children aged 3 to 18 years from a broader peanut OIT trial of low 30 mg (N = 13) vs standard-maintenance dose 300 mg (N = 16) was evaluated. Serum samples were analyzed at two timepoints: before and after one year of dose escalation. Total peanut- and Ara h2-specific immunoglobulin (Ig) G4 and IgE levels were obtained via ELISA.

**Results:** The median participant ages were: 30 mg group—14 years (53% males), 300 mg group—13.5 years (37.5% males). There were no significant differences between groups for both peanut-specific-IgG4 and IgE levels at baseline. After a median escalation phase of 16 months, peanut-specific-IgG4 significantly increased from a baseline of 89.53 μg/mL (IQR 30.03–189.32) to 453.19 μg/mL (IQR 75.77–1466.71) in the 300 mg group (p < 0.001) and from 184.29 μg/mL (IQR 35.00–585.88) to 803.11 μg/mL (IQR 237.12–953.60) in the 30 mg group (p < 0.01). Arah2-specific-IgG4 significantly increased from 62.90 µg/mL (IQR 30.33–106.46) to 306.87 µg/mL (IQR 56.08–6038.12) in the 300 mg group (p < 0.01) and from 99.24 µg/mL (IQR 27.39–216.44) to 386.15 µg/mL (IQR 87.78–989.49) in the 30 mg group (p < 0.001). Peanut-specific-IgE did not show significant changes in either group, from 117.60 ng/mL (IQR 1.56–1401.31) to 699.76 ng/mL (IQR 1.56–1528.86) in the 300 mg group and from 93.38 ng/mL (IQR 47.44–645.00) to 90.82 ng/mL (IQR 23.6–912.00) in the 30 mg group (p > 0.05). The IgG4/IgE ratio for total-peanut and arah2 significantly increased in both groups (p < 0.05).

**Conclusions:** Our findings suggest that the lower, 30 mg dose of peanut OIT induces comparable immunological changes to higher doses in peanut-allergic patients. Larger studies are needed with greater sample sizes.

## 22 Skin prick test using autoclaved peanut extract results in decreased wheal sizes and is associated with lower peanut-specific IgE levels in allergic patients

### Casey G. Cohen^1^, Wei Zhao^1^, Diana Toscano-Rivero^1^, Danbing Ke^2^, Duncan Lejtenyi^2^, Liane Beaudette^2^, Bertrand J. Jean-Claude^1^, Moshe Ben-Shoshan^2^, Bruce D. Mazer^1,2^

#### ^1^Research Institute of the McGill University Health Centre, Montreal, QC; ^2^Division of Allergy and Clinical Immunology, Department of Pediatrics, Montreal Children’s Hospital, Montreal, QC

##### **Correspondence:** Casey G. Cohen

*Allergy, Asthma & Clinical Immunology* 2024, **20(Suppl 1):**22

**Background:** Peanut oral immunotherapy (OIT) is commonly achieved using crushed raw or roasted peanut, although adverse reactions throughout treatment are frequent. Alternative thermal processing methods and their effects on peanut protein allergens have been explored in recent years. High-pressure and temperature autoclaving has been shown to alter overall protein secondary structure and decrease in vitro binding of peanut-specific immunoglobulin E (sIgE) in sera from peanut-allergic patients. However, data assessing clinical responses to the autoclaved peanut are lacking.

**Methods:** Forty-one peanut-allergic individuals were recruited to the Montreal Children’s Hospital for skin prick testing (SPT) using standard commercial peanut extract and an extract created from autoclaved peanuts (130 °C, 30 min) both adjusted to equal protein concentrations. The median age was 20 years old, and 17 subjects were female. Wheal diameters were recorded, and serum samples were obtained to compare peanut-sIgE levels via ELISA. Group comparisons were done using the Wilcoxon signed-rank test.

**Results:** The SPT results showed a significant decrease in wheal diameter in peanut-allergic subjects using the autoclaved peanut extract (mean ± SD = 6.1 ± 5.5 mm) when compared to the commercial standard (11.3 ± 6.4 mm; p < 0.001). Upon stratifying the participants into two groups, one that experienced smaller wheal sizes when using the autoclaved peanut extract (≥ 3 mm less; N = 30) and another with no significant decrease (N = 11), those with smaller wheal sizes to the autoclaved extract demonstrated significantly lower sIgE levels to total peanut (p = 0.029) and allergen components Ara h 1 (p = 0.015), Ara h 2 (p = 0.007), and Ara h 8 (p = 0.017).

**Conclusions:** Altogether, the observed decreases in SPT wheal sizes and sIgE binding levels with the autoclaved peanut suggest that autoclaving decreases allergenicity, making it a potential improved substrate for peanut OIT. Further clinical studies are ongoing to assess the level of safety of the autoclaved peanut in allergic individuals.

## 23 Anaphylactic hypertension: reexamining the definition of anaphylaxis

### Vivian G. Szeto^1^, Kristine Van Aarsen^2^, Tarek Loubani^2^, Harold Kim^2^

#### ^1^McMaster University, Hamilton, ON; ^2^Western University, London, ON

##### **Correspondence:** Vivian G. Szeto

*Allergy, Asthma & Clinical Immunology* 2024, **20(Suppl 1):**23

**Background:** Anaphylaxis is a potentially life-threatening reaction. It is often triggered by the ingestion of a food allergen or sting from a venomous insect [1]. The current definition of anaphylaxis varies but the guiding principle includes the onset of symptoms in two or more organs within minutes to several hours of exposure to the allergen [2]. Presence of hypotension increases the likelihood that the presentation was a severe systemic reaction [3]. However, there is some data that supports the presence of hypertension immediately after exposure to an allergen as a clinical finding; driven by possibly a compensatory vasopressor response. Therefore, this study looks at redefining the definition of anaphylaxis by studying the vitals of patients who present with anaphylaxis.

**Methods:** This is a retrospective cohort study on patients that have visited London Health Sciences Center (LHSC) emergency department between 2012–2022. Using the LHSC electronic medical records, the vitals of patients with a diagnosis of anaphylaxis will be reviewed for hypertension. Hypertension is defined as a systolic blood pressure above 140 [4]. REB approval was obtained from Western University prior to the initiation of this study.

**Results:** Preliminary data has been collected on 664 patients. Within this data set, 34% of the patients had a systolic blood pressure greater than 140 at time of triage while 3% were hypotensive with a systolic less than 90. 138 females presented to the emergency department while 86 were male and the average was 43.8 years old. Within the 224 patients, 12 were admitted to hospital for further monitoring, 1 patient died, with 7 patients leaving after initial treatment and 204 patients formally discharged.

**Conclusions:** Although a proportion of patients presented to the triage with blood pressures in the normal range, there is still a significant number of anaphylactic patients who present to the emergency department with hypertension.

**References**

1. Shaker MS, Wallace DV, K Golden DB, et al. Anaphylaxis-a 2020 practice parameter update, systematic review, and Grading of Recommendations, Assessment, Development and Evaluation (GRADE) analysis. Published online 2020. 10.1016/j.jaci.2020.01.017
2. Turner PJ, Worm M, Ansotegui IJ, et al. Time to revisit the definition and clinical criteria for anaphylaxis? Published online 2019. 10.1016/j.waojou.2019.100066
3. Solmazgul E, Kutlu A, Dogru S, et al. Anaphylactic reactions presenting with hypertension. *Springerplus*. 2016;5[1]. 10.1186/S40064-016-2913-Y
4. Kuehn BM. New Hypertension Guidelines Apply to Diverse Socioeconomic Settings. *JAMA*. 2020;323(23):2363–2363. 10.1001/JAMA.2020.9824

## 24 Modified sesame desensitization protocol in a pediatric cohort

### Pasquale Mulé^1^, Sofianne Gabrielli^1^, Dima Elgendy^1^, Karen Sigman^1^, Christine McCusker^1^, Sarife Saker^1^, Xun Zhang^2^, Moshe Ben-Shoshan^1^

#### ^1^Division of Allergy and Clinical Immunology, Department of Pediatrics, McGill University Health Centre, Montreal, QC; ^2^Centre for Outcomes Research and Evaluation, Research Institute of McGill University Health Centre, Montreal, QC

##### **Correspondence:** Pasquale Mulé

*Allergy, Asthma & Clinical Immunology* 2024, **20(Suppl 1):**24

**Background:** Sesame and sesame-containing foods have become increasingly prevalent in Western diet, meaning that patients allergic to sesame are at risk for severe, life-threatening reactions. We aimed to assess the efficacy and safety of a modified sesame desensitization protocol in children in real-world clinical practice.

**Methods:** Children with a history of sesame allergy and a positive skin prick test to sesame were recruited at the Montreal Children’s Hospital and the Children’s Clinic located in Montreal. After obtaining consent, an initial dose of sesame protein (3–25 mg) was introduced in the form of either tahini muffin or sesame seeds. Patients continued the same dose for 2–5 weeks at home, filled out a symptom diary, and returned to the clinic for updosing until maintenance was reached (2 teaspoons of hummus or 2 mL of tahini). A proportional odds logistic regression model was used, controlling for age and sex, to determine the difference in the severity of allergic reactions between the tahini muffin and sesame seed groups.

**Results:** Between January 2021 and May 2023, 59 children (57% male; median age 2.4 years) were recruited. The majority of patients (74%) had eczema and 18% had asthma. Oral desensitization was performed using one of two strategies according to the allergist: initial doses were either tahini muffin (59%) or sesame seeds (41%). To date, 10 patients (17%) reached the maintenance dose. Ten patients (17%) experienced a non-anaphylactic reaction, and 6 patients (10%) experienced an anaphylactic reaction. The probability of a severe reaction, such as anaphylaxis, was four times higher in the sesame seeds group compared to the tahini muffin group, with an odds ratio (95% CI) of 4.1 (1.1–14.9).

**Conclusions:** Modified sesame desensitization protocols may be safely used in children with sesame allergy.

## 25 Retrospective review of predictors of peanut allergy at a Canadian allergy and immunology clinic

### Mary Wang^2^, Joella Ho^1^, Harold Kim^3,4^, Samira Jeimy^4^

#### ^1^Faculty of Medicine, University of Ottawa, Ottawa, ON; ^2^Western University, London, ON; ^3^Department of Medicine, McMaster University, Hamilton, ON; ^4^Division of Clinical Immunology and Allergy, Department of Medicine, Western University, London, ON

##### **Correspondence:** Mary Wang

*Allergy, Asthma & Clinical Immunology* 2024, **20(Suppl 1):**25

**Background:** Peanut allergy is one of the most common types of food allergy. Identifying predictors for peanut allergy can help in understanding its pathogenesis and detecting individuals at risk before a life-threatening reaction occurs. Our study aimed to identify predictors of peanut allergy from a patient’s clinical history and commonly used investigations.

**Methods:** A retrospective chart review was conducted on 451 patients referred for food allergy assessment at a Canadian hospital-based allergy and immunology clinic between January 2015 and May 2021. Patients who were investigated for peanut allergy were selected for further analysis. Peanut allergy diagnosis was established through unsuccessful oral food challenges or clinical judgment. Simple logistic regression was used to determine the variables predicting peanut allergy, and multiple logistic regression was then used to develop a peanut allergy prediction model with the variables identified.

**Results:** Among the 451 patients investigated for food allergy, 68.0% presented with concerns of peanut allergy. Among these patients, 70.5% were diagnosed with peanut allergy. The age range of patients varied from 8 months to 67 years. The following variables were identified as predictors of peanut allergy: male sex (odds ratio(OR) = 2.28, 95% confidence interval (CI) = 1.38, 3.78), asthma (OR = 2.20, 95%CI = 1.27, 3.90), age at index reaction (OR = 0.94, 95%CI = 0.88, 0.98), skin-prick test wheal size (OR = 1.23, 95%CI = 1.14, 1.34), and Arah2 IgE titres (OR = 1.02, 95%CI = 1.01, 1.03). These variables were used to develop a prediction model for peanut allergy, with 91.59% positive predictive power and 68.57% negative predictive power.

**Conclusions:** A predictive model integrating sex, presence of asthma, age of index reaction, skin-prick wheal size, and Arah2 IgE titres may facilitate safe diagnosis of peanut allergy. Notably, the negative predictive power of this model remains low. Further research should focus on validating this model using a larger cohort and investigating factors that reduce the likelihood of peanut allergy.

## 26 Allergy-friendly food subsidy perceived to reduce burden on families managing food allergy

### Zoe Harbottle^1,2,3^, Michael A. Golding^2,3^, Manvir Bhamra^1,2,3^, Moshe Ben-Shoshan^4^, Jennifer Gerdts^5^, Elissa M. Abrams^2,3^, Sara J. Penner^6^, Jo-Anne St-Vincent^7^, Jennifer L. Protudjer^2,3,8^

#### ^1^Department of Food and Human Nutritional Sciences, University of Manitoba, Winnipeg, MB; ^2^Children’s Hospital Research Institute of Manitoba, Winnipeg, MB; ^3^Department of Pediatrics and Child Health, University of Manitoba, Winnipeg, MB; ^4^Department of Allergy and Immunology, McGill University, Montreal, QC; ^5^Food Allergy Canada, Toronto, ON; ^6^Department of Business and Administration, University of Winnipeg, Winnipeg, MB; ^7^Children’s Allergy and Asthma Education Centre, Winnipeg, MB; ^8^Institute of Environmental Medicine, Karolinska Institutet, Stockholm, Sweden

##### **Correspondence:** Zoe Harbottle

*Allergy, Asthma & Clinical Immunology* 2024, **20(Suppl 1):**26

**Background:** There is a substantial burden placed on families managing food allergy due to the additional effort required and the disproportionate costs of allergy-friendly food products. With the recent increases in food prices, the impact of this burden is further exacerbated. In this study, we aimed to qualitatively evaluate a milk allergy-friendly subsidy program for lower- and middle-income families in Winnipeg, Canada.

**Methods:** As part of an overarching mixed-methods intervention, families living or working within Winnipeg with an annual net household income of ≤ $70,000 in the year prior to recruitment and a child age < 6 years with a physician diagnosed milk allergy were recruited from a database maintained by the principal investigator, social media, and word-of-mouth. From March–August 2022, participating families received bi-weekly deliveries of subsidy kits containing ~ $50 worth of milk allergy-friendly foods. End-of-study semi-structured interviews were conducted with participants to better understand their thoughts on the program. Each interview was audio-recorded, transcribed verbatim, and analyzed thematically.

**Results:** Eight interviews were completed. On average, parents were 29.88 ± 4.39 years old and children were 2.06 ± 1.32 years old. In addition to physician-diagnosed cow’s milk allergy, other food allergies included peanut and egg (each n = 4). We identified three themes: food allergy poses a substantial burden on families, parents prioritize their child’s dietary needs before their own needs, and families perceived this allergy-friendly food subsidy to have emotional and financial benefits.

**Conclusions:** This subsidy was perceived to positively impact families’ food costs and stress. Not only does this demonstrate the need for help for families managing food allergy but also, provides valuable information to inform the development of programs focused on alleviating the burden of food allergy. Future programs should strive to incorporate a greater variety of foods to further the benefits obtained.

## 27 Engaging with experts as part of the evolving conversation on the treatment of anaphylaxis

### Laura May Miles^1^, Jennifer L. Protudjer^2,3,4^, Jennifer Gerdts^5^, Moshe Ben Shoshan^1^

#### ^1^McGill University, Montreal, QC; ^2^University of Manitoba, Winnipeg, MB; ^3^Karolinksa Institutet, Stockholm, Sweden; ^4^George and Fay Yee Centre for Healthcare Innovation, Winnipeg, MB; ^5^Food Allergy Canada, Toronto, ON

##### **Correspondence:** Laura May Miles

*Allergy, Asthma & Clinical Immunology* 2024, **20(Suppl 1):**27

**Background:** Prompt epinephrine use is the first-line treatment for anaphylaxis. Through engagement via a webinar hosted by Food Allergy Canada, we aimed to assess the level of comfort and knowledge surrounding epinephrine autoinjector (EAI) use.

**Methods:** In May 2023, an immunologist and allergy researcher delivered a webinar titled “Epinephrine, Benadryl®, and the evolving conversation on the treatment of anaphylaxis.” Attendees were polled before and after the webinar.

**Results:** The 132 attendees included: 29% (38) legal guardians of a child with a food allergy, 22% (29) health care professionals, 14% (19) adults with a food allergy, 11% (14) dieticians, 11% (14) individuals interested in food allergies, 8% (11) researchers/educators, and 2% (3) caregivers; data were missing for 3% (4). A total of 76 and 73 responses were recorded before and after the presentation, respectively. There was an overall 80% increase in individuals who reported feeling “very comfortable” in EAI use. Top knowledge concerns were uncertainty of reaction severity (76%), lack of confidence in recognizing when to use (52%), and the need to present to the hospital following EAI use (33%). Among responders, 41% reported that, if given guidance on the “wait and see” approach, they would be more likely to use EAI.

**Conclusions:** Expert-led information sessions to patients, caregivers and other health professionals may support improved comfort in EAI use to treat anaphylaxis and contribute to identifying gaps in knowledge of anaphylaxis management.

## 28 Soy-induced anaphylaxis in paediatric patients from Cross-Canada Anaphylaxis Registry (C-CARE)

### Emma Lim^15^, Anne E. Clarke^1^, Judy Morris^2^, Jocelyn Gravel^3^, Rodrick Lim^5^, Edmond S. Chan^4^, Ran D. Goldman^6^, Andrew O’Keefe^7^, Jennifer Gerdts^8^, Derek K. Chu^9^, Julia Upton^10^, Elana Hochstadter^11^, Adam Bretholz^12^, Jennifer L. Protudjer^13^, Elissa M. Abrams^14^, Elinor Simons^14^, Moshe Ben-Shoshan^15^

#### ^1^Division of Rheumatology, Department of Medicine, Cumming School of Medicine, University of Calgary, Calgary, AB; ^2^Department of Emergency Medicine, Sacré-Coeur Hôpital, Montreal, QC; ^3^Department of Emergency Medicine, Sacré-Coeur Hôpital, Montreal, QC; ^4^Division of Allergy, Department of Pediatrics, BC Children’s Hospital, University of British Columbia, Vancouver, BC; ^5^Division of Pediatric Emergency Medicine, Department of Pediatrics, Children’s Hospital at London Health Science Centre, London, ON; ^6^Division of Clinical Pharmacology and Emergency Medicine, Department of Pediatrics, BC Children’s Hospital, University of British Columbia, Vancouver, BC; ^7^Department of Pediatrics, Faculty of Medicine, Memorial University, St. John’s, NL; ^8^Food Allergy Canada, Toronto, ON; ^9^Division of Clinical Immunology & Allergy, Department of Medicine, and Department of Health Research Methods, Evidence & Impact, McMaster University, Hamilton, ON; ^10^Division of Immunology and Allergy, Department of Pediatrics, The Hospital for Sick Children, Department of Paediatrics, University of Toronto, Toronto, ON; ^11^Department of Pediatric Emergency Medicine, The Hospital for Sick Children, University of Toronto, Toronto, ON; ^12^Division of Pediatric Emergency Medicine, Department of Pediatrics, Montreal Children’s Hospital,, Montreal, QC; ^13^Department of Pediatrics and Child Health, Children’s Hospital Research Institute of Manitoba, Winnipeg, MB; ^14^Department of Pediatrics and Child Health, Section of Allergy and Clinical Immunology, Children’s Hospital Research Institute of Manitoba, Winnipeg, MB; ^15^Department of Pediatrics, Montreal Children’s Hospital, McGill University, Montreal, QC

##### **Correspondence:** Emma Lim

*Allergy, Asthma & Clinical Immunology* 2024, **20(Suppl 1):**28

**Background:** Evaluate the clinical features and treatment strategies employed in paediatric cases of soy-induced anaphylaxis, while also identifying factors linked to epinephrine usage.

**Methods:** Between 2011–2023, data was collected on soy-induced anaphylaxis in children presenting to paediatric emergency departments (ED) utilizing standardized recruitment forms, as part of the Cross-Canada Anaphylaxis Registry (C-CARE). Multivariable logistic regression was used to evaluate factors associated with epinephrine usage and severe reactions (cyanosis, respiratory arrest, hypotension, circulatory collapse, dysrhythmia, bowel incontinence, cardiac arrest or serious bradycardia, confusion, and unconsciousness).

**Results:** Soy-induced anaphylaxis (n = 46) accounted for 1% of all food-induced anaphylaxis reactions (n = 4393). Of these, 65.2% occurred in males, and the median age was 9.4 years (IQR = 11.1). Soy sauce and tofu were the most common food exposures, triggering 17.0% and 13.2% of reactions, respectively. The majority of reactions were moderate (87.0%), while severe (8.7%) and mild (4.3%) reactions were less common. The most common symptoms were pruritus (76.1%), urticaria (63.0%), and angioedema (60.9%). Severe reactions were associated with physician diagnosed asthma [aOR (1.29 (95% CI 1.06, 1.58)]. Pre-ED epinephrine administration occurred in 60.9% of cases (n = 28) and was strongly associated with other known allergies [adjusted odds ratio (aOR) 1.65 (95% CI 1.26, 2.18)]. Among children with soy-induced anaphylaxis, 69.5% had a known food allergy. Only 26.1% of these were soy allergies, while 39.1% were peanut allergies. Children with soy-induced anaphylaxis who had known food allergies were more likely to be male [aOR 1.38 (95% CI 1.06, 1.80)].

**Conclusions:** In patients who presented with soy-induced anaphylaxis, Individuals with known allergies are more likely to receive epinephrine prior to presentation in the emergency department. Severe reactions are more likely to occur in individuals with diagnosed asthma. Children who experience anaphylaxis to soy are more likely to have a physician diagnosed peanut allergy.

## 29 The impact of allergic disease on the quality of parent–child relationships: a scoping review

### Rebecca Kim^1,2^, Cari Slayen^3^, Anthony Lagunda^4^, Michael A. Golding^2,3^, Leslie E. Roos^3^, Camelia E. Hostinar^4^, Jennifer L. Protudjer^2,3,5^

#### ^1^Western University, London, ON; ^2^The Children’s Hospital Research Institute of Manitoba, Winnipeg, MB; ^3^University of Manitoba, Winnipeg, MB; ^4^University of California, Davis, CA, USA; ^5^Karolinska Institutet, Stockholm, Sweden

##### **Correspondence:** Rebecca Kim

*Allergy, Asthma & Clinical Immunology* 2024, **20(Suppl 1):**29

**Background:** Atopic dermatitis (AD), food allergy, and asthma are common pediatric conditions, which are negatively associated with health-related quality of life. Previous research has primarily focused on the association between asthma and parent–child relationships, leaving uncertainty regarding the consequences of AD and food allergy on this relationship. We aimed to conduct a scoping review of the literature examining associations between allergic disease and parent–child relationships.

**Methods:** We conducted a scoping review, informed through consultation with a health sciences librarian. Articles involving the quality of parent–child relationships amongst children (< 18 years) with AD, food allergy, or asthma were included. Searches were conducted in the MEDLINE, PsycInfo, and Scopus databases, and limited to original research published in English, French, or Spanish. After the initial search, two co-authors independently screened studies against the inclusion criteria. A secondary search was completed to identify eligible articles. We identified common themes between the included articles, by specific condition and cross-conditions. As findings for asthma were different than AD and food allergy, we compared asthma with the latter two conditions.

**Results:** In total, 860 articles were identified, of which 28 articles (3.3%) were included. Parents of children with asthma exhibited more overprotective/intrusive parenting styles compared to parents of healthy children, as indicated by common themes across included articles. Although inconsistent, some studies also observed higher levels of critical attitudes among parents of children with asthma, and lower levels of attachment security among their children. There were no eligible studies addressed the relationship between food allergy and parent–child attachment, while three studies explored the relationship between AD and parent–child relationship quality, for which the results were mixed.

**Conclusions:** Asthma seemingly contributes to more overprotective/intrusive parenting styles, and potentially decreased attachment security amongst children with vs. without allergic conditions. Corresponding studies for AD and food allergy are limited.

## 30 Relationship between age and IgE levels to reactivity thresholds during double-blind, placebo-controlled peanut challenges

### Douglas Mack^1,2^, Philippe Bégin^3^, Edmond S. Chan^4^, Katharine J. Bee^5^, Henry T. Bahnson^5^, Todd D. Green^5,6^

#### ^1^McMaster University, Hamilton, ON; ^2^Halton Pediatric Allergy, Burlington, ON; ^3^CHU Sainte-Justine, Montreal, QC; ^4^British Columbia Children’s Hospital & The University of British Columbia, Vancouver, BC; ^5^DBV Technologies, Montrouge, France; ^6^UPMC Children’s Hospital of Pittsburgh and University of Pittsburgh School of Medicine, Pittsburgh, PA, USA

##### **Correspondence:** Douglas Mack

*Allergy, Asthma & Clinical Immunology* 2024, **20(Suppl 1):**30

**Background:** Reactivity thresholds can help assess risk from allergenic foods and provide critical information for the management of food allergy [1]. However, there is an unmet need to identify predictors of reaction thresholds as they currently have not been estimated with certainty and vary significantly per individual [1, 2]. Two Phase 3, multicenter, randomized, double-blind, placebo-controlled clinical trials, EPITOPE and PEPITES, were designed to evaluate the safety and efficacy of a peanut patch containing 250 µg peanut protein (VP250) for 12 months in children aged 1 through 3 years and 4 through 11 years, respectively [3, 4]. These studies provided an extensive database of double-blind, placebo-controlled food challenges (DBPCFC) to understand the relationship between baseline factors, such as age and IgE levels, and reaction thresholds.

**Methods:** DBPCFCs were performed per PRACTALL guidelines at entry in peanut-allergic children. Oral food challenges (OFC) were discontinued when symptoms met prespecified stopping criteria. A linear regression analysis was performed to assess the relationship between eliciting dose (ED) and baseline age and sIgE in all children screened in the EPITOPE and PEPITES studies.

**Results:** In combination, 769 children were randomized. The last ingested dose and baseline ED were negatively correlated with age, sIgE (P < 0.0001), and Ara h2 sIgE (P < 0.0001).

**Conclusions:** In this analysis of a large database of DBPCFC’s to peanut, age and sIgE are negatively correlated with baseline ED. Consistent with other studies, high sIgE here is associated with a lower ED [5], while age is positively correlated with sIgE [6]. Just as food allergy prevention and treatment studies suggest increased plasticity of the allergic immune system and greater success rates at younger ages, these data may suggest an increased benefit to initiating peanut allergy treatment early after diagnosis.

**References**

1. Crevel RW, Ballmer-Weber BK, Holzhauser T, et al. *Allergy*. 2008 May;63(5):597–609.
2. Moneret-Vautrin DA, Kanny G. *Curr Opin Allergy Clin Immunol*. 2004 Jun;4(3):215–9.
3. Greenhawt M, Sindher SB, Wang J, et al. *NEJM*. 2023 May 11;388(19):1755–1766.
4. Fleischer DM, Greenhawt M, Sussman G, et al. *JAMA*. 2019 Mar 12;321(10):946–955.
5. Yanagida N, Sato S, Ebisawa M. *Curr Opin Allergy Clin Immunol*. 2023 Jun 1;23(3):226–232.
6. van der Zee T, Dubois A, Kerkhof M, van der Heide S, Vlieg-Boerstra B*. J Allergy Clin Immunol*. 2011 Nov;128(5):1031–6.

## 31 Efficacy and safety of egg oral immunotherapy in children

### Tydia Allouche, Danbing Ke, Liane Beaudette, Duncan Lejtenyi, Manel Ouamrane, Mackenzie Lawson, Christine McCusker, Bruce D. Mazer

#### McGill University Hospital Center, Montreal, QC

##### **Correspondence:** Tydia Allouche

*Allergy, Asthma & Clinical Immunology* 2024, **20(Suppl 1):**31

**Background:** Egg Oral Immunotherapy (OIT) has become a treatment option to desensitize individuals allergic to hen’s egg. Though promising, better understanding of efficacy and safety of egg OIT is needed.

**Methods:** Participants with egg allergy were confirmed by oral food challenges. Twenty-six underwent OIT at the Montreal Children’s Hospital. During dose escalation, graded doses were given in-clinic followed by at-home dosing as instructed until the target dose of 300 mg egg protein was reached. Adverse reactions were recorded following each dosing if present and their severity graded. Anaphylaxis was determined according to clinical criteria by Sampson et al. 2006. Risk factors associated with anaphylaxis were examined via Poisson regression.

**Results:** Among 26 participants eligible for egg OIT, 58% were females and mean age was 12.2 years. Apart from 9 in progress and one withdrawn, 15 out of 16 (93.8%) participants reached 300 mg, including 6 crossed over following one-year observation. The threshold doses in these 6 participants were comparable before and after the observation (Wilcoxon test, p = 0.1). During dose escalation, a total of 6859 doses were taken, and 1190 episodes of adverse reactions were recorded, in which 53.7% involved the gastro-intestinal system 21.2% involved the mucocutaneous system, and 8.9% involved the respiratory system. Organ systems affected varied widely by individuals. The three most common complaints were abdominal discomfort, oral pruritus and nausea. In total, 1.5% (18/1190) episodes of adverse reactions were anaphylaxis. Factors (Incidence rate ratios, Confidence Interval) significantly associated with anaphylaxis included Male (3.59, 1.37–10.56, p = 0.013), eczema (0.32, 0.11–0.84, p = 0.025), as well as cumulative dose [log] (0.63, 0.46–0.83, p = 0.002) and wheal size of skin prick test (0.84, 0.73–0.95, p = 0.01).

**Conclusions:** Egg OIT is effective with high success rate of desensitization and low incidence of severe adverse reactions.

## 32 Optimization of a novel casein-specific anaphylactic mouse model

### Wei Zhao, Eisha Ahmed, Nicholas Vonniessen, Casey Cohen, Bruce Mazer

#### The Research Institute of the McGill University Health Centre and the Department of Pediatrics, Faculty of Medicine, McGill University, Montreal, QC

##### **Correspondence:** Wei Zhao

*Allergy, Asthma & Clinical Immunology* 2024, **20(Suppl 1):**32

**Background:** Cow’s milk allergy, caused by ingestion of proteins such as casein, is one of the leading causes of anaphylaxis in children. Mouse models that mimic symptoms of anaphylaxis in humans are needed to evaluate potential new therapies for food allergy. According to the atopic march, severe eczema is associated with food allergy in children and may be the portal of sensitization. Therefore, we established a mouse model in which the mice are transdermally sensitized.

**Methods:** Six- to eight-week-old male and female C57BL/6 mice (n = 3–5) were exposed via gently abraded skin to dissolved casein or PBS weekly for six weeks. Two weeks after the last sensitization, all mice were challenged intragastrically with 50 mg of dissolved milk powder. The challenged mice were observed with live and infrared cameras to assess clinical symptoms and skin surface temperature. Following sacrifice, tissue samples including spleen, inguinal lymph nodes, and axillary lymph nodes were assessed. We also compared transdermal and subcutaneous sensitizations to determine the more effective route of eliciting anaphylactic reactions in mice.

**Results:** Following casein sensitization, we detected casein-specific IgE in all sensitized female mice which peaked on day 28. IgE+ B cells were significantly increased in the inguinal lymph nodes of sensitized female mice compared to controls. We also observed signs of moderate anaphylactic reactions including continuous scratching and decreased reactivity in the female casein-sensitized mice. We were unable to detect an increase in casein-specific IgE in subcutaneously sensitized mice.

**Conclusions:** These findings suggest that four weekly transdermal sensitizations were sufficient to induce systemic reactions in intragastrically challenged female C57BL/6 mice. This mouse model can serve as a preclinical model to determine the efficacy of potential treatments for food allergy.

## 33 CRAFT: Canadian registry for anaphylaxis fatalities

### Khaled M. Hazzazi^1^, Jennifer Protudjer^2^, Elissa Abrams^2^, Waleed Alqurashi^3^, Edmond S. Chan^4^, Anne Ellis^5^, Jennifer Gerdts^6^, Tim Vander Leek^7^, Rod Lim^8^, Paul-Andre Perron^9^, Moshe Ben-Shoshan^1^

#### ^1^Department of Pediatrics, Montreal Children’s Hospital, McGill University, Montreal, QC; ^2^Children’s Hospital Research Institute of Manitoba, Winnipeg, MB; ^3^Department of Pediatrics and Emergency Medicine, University of Ottawa, Ottawa, ON; ^4^Division of Allergy, Department of Pediatrics, BC Children’s Hospital, University of British Columbia, Vancouver, BC; ^5^Kingston Health Sciences Centre, Kingston, ON; ^6^Executive Director, Food Allergy Canada, Toronto, ON; ^7^Department of Pediatrics, Division of Clinical Immunology and Allergy, University of Alberta, Edmonton, AB; ^8^Science Centre, London, ON; ^9^Bureau du coroner, Québec, QC

##### **Correspondence:** Khaled M. Hazzazi

*Allergy, Asthma & Clinical Immunology* 2024, **20(Suppl 1):**33

**Background:** Anaphylaxis is the most severe manifestation of an allergic reaction, affecting more than 2% of the North American population, with fatalities rare but reported. Detailed data of fatal anaphylaxis in Canada are scarce. We aimed to assess cases of anaphylaxis fatality in Canada, between January 2020 and June 2023, and identify risk factors.

**Methods:** The CRAFT: Canadian Registry for Anaphylaxis Fatalities includes data, from January 2020 onward, on fatal anaphylaxis, obtained from social media, professional associations, patient organizations (Food Allergy Canada, Canadian Anaphylaxis Initiative) and through coroners’ reports. Anaphylaxis was operationalised as coroner-reported cases of fatal anaphylaxis, even if the history was not consistent with National Institute of Allergy and Infectious Disease (NIAID) definition of anaphylaxis, namely involvement of at least two organ systems/hypotension [1]. Probable anaphylaxis was operationalised as a history consistent with this NIAID definition.

**Results:** Between January 2020 and June 2023, 10 cases of fatal anaphylaxis were identified. The male to female ratio was 1:1, five were adults, and five were defined as probable anaphylaxis. Suspected triggers included food (3/10; including one case of each: milk, peanut, and pine nut), drugs (3/10; including one case each of Septra, amoxicillin, iodine contrast media), insect allergy (1/10; wasp), dog hair (1/10), and unknown (1/10). Reported comorbidity included asthma (5/10) and heart disease (2/10). About half (5/9) received pre-hospital epinephrine. Timing of epinephrine administration was available for four cases (one was after five minutes, one after 15 min, one after 1 h [as an inpatient], and one after few hours).

**Conclusions:** Consistent with data from other countries, food and drug were the main triggers of fatal anaphylaxis, as was the delayed use of epinephrine. Asthma was a common comorbidity. Continued patient- and population-level education regarding allergen avoidance, and the prompt use of epinephrine are needed.

**Reference**

1. Boyce JA, Assa’ad A, Burks AW, et al. Guidelines for the diagnosis and management of food allergy in the United States: report of the NIAID-sponsored expert panel. *J Allergy Clin Immuno*l. 2010;126(6 Suppl):S1–58.

## 34 Predominant cofactors for epinephrine administration during preschool oral immunotherapy

### Alexandra Baaske^1^, Edmond S. Chan^1^, Raymond Mak^1^, Tiffany Wong^1^, Stephanie Erdle^1^, Scott B. Cameron^1^, Joanne Yeung^1^, Sandeep Kapur^2^, Mary McHenry^2^, Gregory A. Rex^2^, Sara Leo^1^, Thomas V. Gerstner^3^, Victoria E. Cook^1^, Lianne Soller^1^

#### ^1^University of British Columbia, Vancouver, BC; ^2^Dalhousie University, Halifax, NS; ^3^University of Manitoba, Winnipeg, MB

##### **Correspondence:** Alexandra Baaske

*Allergy, Asthma & Clinical Immunology* 2024, **20(Suppl 1):**34

**Background:** Patients may experience reactions requiring epinephrine during oral immunotherapy (OIT) for food allergy, and certain cofactors may reduce the patient’s threshold at which they experience symptoms. We examined cofactors reported at the time of epinephrine administration for patients receiving OIT as part of a Canada-wide quality improvement project.

**Methods:** Canadian academic and community allergists administered OIT to predominantly preschool-aged patients. Data collection forms capturing information about epinephrine administration, including whether the patient was on OIT buildup or maintenance, and cofactors present, were analyzed.

**Results:** Between July 1, 2017 and May 16, 2023, 126 epinephrine administration forms were completed for 88 OIT patients. Of these, median age at start of OIT was 47 months (IQR: 30.5, 69.5), 70.5% of patients were male, 78.4% had one or more atopic conditions (asthma, eczema, allergic rhinitis), and 62.5% were receiving single-food OIT. Epinephrine administrations occurred during build-up (75.4%; n = 95) and maintenance (24.6%; n = 31). There were 21 patients (23.9%) who received epinephrine on multiple occasions (2 occasions, 10.2%; 3 occasions, 9.1%; 4 occasions, 2.3%; 5 occasions, 2.3%). Of the 88 patients who received epinephrine, 42 (47.7%) had ≥ 1 cofactors present at the time of their reaction. The most common were: comorbid illness (40.5%), vigorous exercise (23.8%), and dosing on an empty stomach (23.8%). Other cofactors included: Dosing errors (n = 7), Emotional stress/anxiety (n = 3), and dosing during pollen season in patients who are grass-pollen allergic (n = 2).

**Conclusions:** Comorbid illness, vigorous exercise, and taking OIT doses on an empty stomach appear to be important cofactors present at the time of allergic reaction with epinephrine administration. A discussion about cofactors should be included in the educational aspect of the enrollment process for OIT. Future research will examine potential differences on the impact of cofactors on reactions during build-up versus maintenance, and whether there are other risk factors for epinephrine use beyond cofactors.

## 35 Can patients who required epinephrine after administration of the COVID-19 vaccine safely receive the vaccine in the future?

### Sarah K. Lohrenz, Godfrey Lam, Amin Kanani

#### University of British Columbia, Vancouver, BC

##### **Correspondence:** Sarah K. Lohrenz

*Allergy, Asthma & Clinical Immunology* 2024, **20(Suppl 1):**35

**Background:** Currently the Centers for Disease Control (CDC) and the World Allergy Organization (WAO) recommend against readministration of a second dose of a SARS-CoV-2 mRNA vaccine in those with moderate-severe immediate type reactions. However, accumulating data supports readministration in a supervised setting [1].

**Methods:** We performed a retrospective analysis of patients who were able to tolerate a SARS-CoV-2 mRNA vaccine challenge after receiving epinephrine with their initial dose. Charts were reviewed in allergy/immunology offices in Vancouver from December 14 2020 to November 30, 2022.

**Results:** 9 patients were identified, 8/9 were female, age range was 18–71 years, and the median age was 47 years. All reactions occurred within 1 h of vaccine administration. The most common symptoms reported were dyspnea, cough, dysphagia, oropharyngeal swelling, dizziness, flushing/pruritus, nausea and vomiting. Hypotension was not a prominent feature. 7/9 reactions were to the Pfizer vaccine, 1/9 to the Moderna and 1/9 due to AstraZeneca. All patients required between 1–5 doses of epinephrine. 7/9 had negative intradermal testing to the SARS-CoV-2 mRNA vaccine, 1 patient had an equivocal test and 1 patient did not undergo testing. All patients (9/9) tolerated graded administration of the Pfizer SARS-CoV-2 mRNA vaccine in an allergy specialist clinic.

**Conclusions:** To our knowledge, there have been no dedicated studies looking at the success of SARS-CoV-2 mRNA vaccine challenges after an initial systemic reaction specifically requiring epinephrine. Our data supports the safety of SARS-CoV-2 mRNA revaccination in patients who received epinephrine with a previous vaccination after careful evaluation by an allergy specialist. Administration of epinephrine should not be considered an absolute contraindication to future vaccination.

**Reference**

1. Chu DK, Abrams EM, Golden DBK, et al. Risk of Second Allergic Reaction to SARS-CoV-2 Vaccines: A Systematic Review and Meta-analysis. *JAMA Intern Med*. 2022;182(4):376-385. 10.1001/jamainternmed.2021.8515

## 36 Low-dose food reintroduction and gradual advancement may be a safe approach in management of mild-to-moderate food protein-induced enterocolitis syndrome to solid foods

### Linlei Ye^1^, Samira Jeimy^2^, Timothy K. Vander Leek^3^, Elana Lavine^4^, Tiffany Wong^5^, Stephanie C. Erdle^5^, Victoria E. Cook^5^

#### ^1^Department of Pediatrics, University of Alberta, Edmonton, AB; ^2^Division of Clinical Immunology and Allergy, Department of Medicine, Western University, London, ON; ^3^Pediatric Allergy and Immunology, Department of Pediatrics, University of Alberta, Edmonton, AB; ^4^Department of Pediatrics, University of Toronto, Humber River Hospital, Toronto, ON; ^5^Division of Allergy, BC Children’s Hospital, Vancouver, BC

##### **Correspondence:** Linlei Ye

*Allergy, Asthma & Clinical Immunology* 2024, **20(Suppl 1):**36

**Background:** Current long-term management of acute food protein-induced enterocolitis syndrome (FPIES) involves strict avoidance of the food trigger for 12–18 months, followed by resource-intensive oral food challenges (OFC). However, prolonged avoidance may increase the risk of IgE-mediated food allergy and adversely impacts quality of life. For IgE-mediated food allergy, food ladders have shown success in accelerating tolerance for egg and milk. Our case series evaluated the safety of low-dose food reintroduction and gradual advancement in patients with mild-to-moderate FPIES to solid foods (non-egg, non-milk).

**Methods:** Between 2020 to 2022, pediatric patients with mild-to-moderate FPIES to solid foods were reintroduced to the food trigger within 6 months after their last reaction. Beginning with a grain-sized amount, portions were gradually increased and provided on a regular basis. Patients were reassessed every 3–6 months, at which time treating allergists collected information on symptoms experienced and provided instructions for dose advancement. If symptoms were experienced, patients returned to the previously tolerated dose. All patients were offered a prescription for ondansetron. Descriptive statistics were analyzed using Excel.

**Results:** Fifteen patients with mild-to-moderate FPIES to solid foods were reintroduced to the offending food at a median time of 3 months (IQR, 2–4) after their last reaction. The most common food trigger was oat (27%, n = 4). All patients successfully tolerated an age-appropriate serving of food within a median duration of 5 months (IQR, 3–8). Four (27%) patients experienced symptoms during the intervention, including belching (20%), abdominal discomfort (6.7%), and a single episode of vomiting (6.7%). No patients required acute medical assessment or intravenous fluids. No patient discontinued the intervention.

**Conclusions:** This small case series demonstrated that allergist-guided gradual food reintroduction and advancement within 6 months of the most recent FPIES reaction may be a safe approach in some patients with mild-to-moderate FPIES to solid foods.

## 37 Safety of home initiation of the Canadian Egg Ladder in children with isolated cutaneous symptoms following egg exposure

### Lu (Nancy) Yu^1^, Samira Jeimy^1^, Stephanie C. Erdle^2^, Tiffany Wong^2^, Victoria Cook^2,3^

#### ^1^Division of Clinical Immunology and Allergy, Dept of Medicine, London, ON; ^2^Division of Allergy, BC’s Children’s Hospital, Vancouver, BC; ^3^Community Allergy Clinic, Victoria, BC

##### **Correspondence:** Lu (Nancy) Yu

*Allergy, Asthma & Clinical Immunology* 2024, **20(Suppl 1):**37

**Background:** Patients with cutaneous symptoms following egg exposure are often referred to allergy and instructed to avoid egg in all forms. The Canadian Egg Ladder has demonstrated safety in patients with IgE-mediated allergy to egg [1], and home ladder initiation appears to be safe in egg-allergic patients [2]. Initiation of the ladder by non-allergist providers in patients with isolated cutaneous symptoms after egg exposure may facilitate reintroduction of egg into the diet and reduce the need for allergist assessment. This case series describes safety of home egg ladder initiation in patients with isolated cutaneous symptoms following egg exposure.

**Methods:** Between 2018 and 2022, patients 0 to 3 years old with isolated local or generalized cutaneous symptoms following egg exposure were instructed to start the egg ladder at home. Written instructions and an epinephrine autoinjector were provided. Patients were followed every 3–6 months to monitor progress. Ladder completion was defined as ingestion of an age-appropriate serving sized of cooked egg. Baseline demographics were recorded. Descriptive statistics were analyzed with Excel.

**Results:** 43 patients with isolated cutaneous symptoms were started on the egg ladder. Median age at initiation was 11 months (IQR 8–14mo). Twenty (39%) patients tolerated egg in baked form prior to or following the initial reaction. All patients completed the ladder, with an average time to completion 4.4 months (IQR 2–5mo). Eight patients (19%) experienced an adverse reaction. Most (n = 5, 63%) were rashes limited to local contact urticaria; none required epinephrine or ED visit.

**Conclusions:** This case series demonstrates that the egg ladder can safely guide reintroduction in pediatric patients with isolated cutaneous symptoms following egg exposure and provides additional support for the safety of at home ladder initiation. Use of this intervention in low-risk children by non-allergist providers could facilitate earlier reintroduction of egg into the diet and may reduce the need for allergist assessment.

**References**

1. Chomyn A, Chan ES, Yeung J, Vander Leek TK, Williams BA, Soller L, Abrams EM, Mak R, Wong T. Canadian food ladders for dietary advancement in children with IgE-mediated allergy to milk and/or egg. Allergy Asthma Clin Immunol. 2021 Aug 5;17[1]:83. 10.1186/s13223-021-00583-w. PMID: 34353372; PMCID: PMC8340453.
2. Thomas L, Belcher J, Phillips R, et al. Use of an egg ladder for home egg introduction in children with IgE-mediated egg allergy. *Pediatr Allergy Immunol*. 2021; 32(7): 1572- 1574. 10.1111/pai.13541

## 38 Utilization of telephone support service by patients undergoing oral immunotherapy

### Victor Paradis, Anne Des Roches, Charles Elbany, Camille Braun, François Graham, Kathryn Samaan, Roxane Labrosse, Louis Paradis, Philippe Bégin

#### CHU Sainte-Justine, Montréal, QC

##### **Correspondence:** Victor Paradis

*Allergy, Asthma & Clinical Immunology* 2024, **20(Suppl 1):**38

**Background:** Oral immunotherapy (OIT) is the only available treatment for food allergies. A telephone support service managed by nurses and operators from the Network Activities Coordination Center (CCAR) has been established at CHU Sainte-Justine to assist parents during the OIT protocol, due to the risk of allergic reactions. The objective of this study was to evaluate the utilization of this service within the context of OIT and to determine the reasons for seeking assistance.

**Methods:** This was a retrospective study using patient records who contacted the call center regarding their oral immunotherapy. Data were collected using an Excel file, and descriptive analysis was conducted using means, standard deviations, medians, and percentages.

**Results:** Between January 2017 and December 2020, 884 children were undergoing OIT, and 907 parental calls were received and handled by the nurses of the service during clinic hours (n = 843) for 396 children (89.8%) and buy CCAR during evenings and weekends (n = 64) for 45 children (10.2%). The most frequent reasons for calls during daytime were related to reactions following dose intake (n = 374; 37.2%) and dose adjustments during infectious episodes (n = 209; 19.3%). Symptoms reported by parents were mainly gastrointestinal (n = 365; 24.5%) and cutaneous (n = 350; 23.4%). During off-hours, the most frequent reasons were also related to reactions following dose intake (n = 31; 39.2%) and dose adjustments during infectious episodes (n = 12; 15.2%). The on-call allergist was consulted for 20 (31.3%) of off-hours calls received by CCAR.

**Conclusions:** This study shows that most OIT support calls occur during the day. The amount of off-hours calls is low and can be included in existing pediatric support lines without requiring dedicated OIT nurses to be on-call 24/7.

***Victor Paradis—CSACI Summer Studentship Award winner***

## Immunology

## 39 Once-daily oral berotralstat led to improvements in work productivity and activity impairment in patients with hereditary angioedema: results from the APeX-2 randomized phase III trial

### Rémi Gagnon^1^, William R. Lumry^2^, Teresa Caballero^3^, Douglas T. Johnston^4^, Dianne Tomita^4^, Bhavisha Desai^4^, Daniel F. Soteres^5^

#### ^1^Clinique spécialisée en allergie de la capitale, Québec, QC; ^2^Allergy and Asthma Specialists of Dallas, Dallas, TX, USA; ^3^Hospital Universitario La Paz, Madrid, Spain; ^4^BioCryst Pharmaceuticals, Inc., Durham, NC, USA; ^5^Asthma & Allergy Associates and Research Center, P.C., Colorado Springs, CO, USA

##### **Correspondence:** Rémi Gagnon

*Allergy, Asthma & Clinical Immunology* 2024, **20(Suppl 1):**39

**Background:** Berotralstat is a first-line, once-daily (QD) oral prophylaxis for hereditary angioedema (HAE), which has demonstrated sustained attack rate reduction in clinical trials [1]. Here we present the impact of berotralstat on work productivity and activity impairment, as measured by the Work Productivity and Activity Impairment (WPAI) Questionnaire, in patients from APeX-2 (NCT03485911).

**Methods:** APeX-2 was a randomized, placebo-controlled, parallel group, Phase III trial that evaluated the efficacy and safety of oral berotralstat 110 and 150 mg in patients ≥ 12 years with type I or II HAE [1, 2]. The WPAI Questionnaire was administered at baseline and several predefined visits during the study. The questionnaire consists of absenteeism, presenteeism, work productivity loss, and activity impairment domains. WPAI outcomes are expressed as percentages; higher scores indicate greater impairment or less productivity. A reduction in score indicates improvement. WPAI scores for patients who received berotralstat 150 mg QD from Day 1 are summarized descriptively using mean percentages ± standard error of the mean values.

**Results:** In total, 40 patients received berotralstat 150 mg QD from Day 1. Work productivity loss improved from 36.0 ± 6.16 at baseline (n = 25) to 18.1 ± 5.01 (n = 25), 21.0 ± 5.45 (n = 27), 17.5 ± 5.25 (n = 22), and 1.7 ± 1.67 (n = 12) after 4, 24, 48, and 96 weeks of berotralstat treatment, respectively. Activity impairment improved from 35.8 ± 4.46 at baseline (n = 40) to 17.4 ± 3.78 (n = 38), 21.6 ± 4.23 (n = 38),15.3 ± 4.12 (n = 34), 6.3 ± 3.52 (n = 19) after 4, 24, 48, and 96 weeks of berotralstat treatment. Absenteeism, which was low at baseline, and presenteeism also improved.

**Conclusions:** Patients with HAE who received long-term treatment with oral berotralstat 150 mg in the APeX-2 study reported improvements in work productivity and activity impairment. Improvements across all four domains were observed as early as Week 4 and were sustained through 96 weeks of berotralstat treatment.

**References**

1. Zuraw B, Lumry WR, Johnston DT, et al. Oral once-daily berotralstat for the prevention of hereditary angioedema attacks: A randomized, double-blind, placebo-controlled phase 3 trial. *J Allergy Clin Immunol*. 2021 Jul; 148[1]: 164–72.e9. 10.1016/j.jaci.2020.10.015. Epub 2020 Oct 21.
2. Wedner HJ, Aygören-Pürsün E, Bernstein J, et al. Randomized trial of the efficacy and safety of berotralstat (BCX7353) as an oral prophylactic therapy for hereditary angioedema: results of APeX-2 through 48 weeks (part 2*). J Allergy Clin Immunol Pract.* 2021; 9: 2305–14.e4.

## 40 Optimizing DC-based strategies for HIV-1 vaccine development: insights from gene transfer efficiency enhancement and immunization regimens

### Alireza Milani, Azam Bolhassani

#### Department of Hepatitis and AIDS, Pasteur Institute of Iran, Tehran, Iran

##### **Correspondence:** Alireza Milani

*Allergy, Asthma & Clinical Immunology* 2024, **20(Suppl 1):**40

**Background:** The development of an effective HIV-1 vaccine remains a significant challenge in the field of immunology. Dendritic cells (DCs) play a critical role in orchestrating immune responses, making them an attractive target for vaccine design. In this study, our aim was to design a novel HIV-1 vaccine using DC-based strategies and Hsp27 as a natural adjuvant.

**Methods:** In this study, the Hsp27-Nef construct was considered as a promising candidate for HIV-1 vaccine development. To optimize the transfection efficiency of DCs derived from mouse bone marrow cells, a comprehensive approach involving cell heating at three different stages (before, during, and after transfection) was implemented. The cells underwent a 2-h heat treatment at 42 °C. The method that demonstrated the highest efficiency of gene transfer was selected for immunization. Additionally, a subset of DCs was pulsed with Hsp27-Nef protein and subsequently utilized for immunization. Moreover, the heterologous DC^DNA^ prime/DC^protein^ boost regimen was employed for immunization in BALB/c mice. Finally, immune responses were assessed by monitoring antibodies, IFN-γ and IL-5 cytokines, as well as Granzyme B secretion.

**Results:** Our results demonstrated that heat treatment of the cells before transfection for 2 h at 42 °C significantly increased the efficiency of gene transfer in DCs. Moreover, immunization with the heterologous DC^DNA^ prime/DC^protein^ approach utilizing Hsp27-Nef resulted in elevated levels of IgG2a and IFN-γ, which are indicative of Th1 responses, as well as increased secretion of Granzyme B, compared to other immunization strategies. Furthermore, the incorporation of Hsp27 provided an effective means of stimulating robust immune responses.

**Conclusions:** These findings highlight the importance of optimizing transfection conditions for efficient antigen delivery in HIV-1 DC-based vaccine design. Furthermore, the insights gained from this study make a valuable contribution to the ongoing endeavors aimed at developing an effective HIV-1 vaccine.

## 41 Functional and phenotypic characterization of novel loss-of-function variants in CTLA4

### Sean Duke^1^, James Maiarana^2^, Pariya Yousefi^1^, Samantha Gerrie^1^, Cornelius Boerkoel^1^, Ali Amid^1^, Dewi Schrader^1^, Orlee Guttman^1^, Sally Lawrence^1^, Meera Rayar^1^, Connie Yang^1^, Anna F. Lee^1^, Amin Kanani^3^, Persia Pourshahnazari^3^, Audi Setiadi^1^, Jacob Rozmus^1^, Stephanie Erdle^1^, Kyla Hildebrand^1^, Elliot James^1^, Stuart Turvey^1^, Janet Markle^2^, Catherine M. Biggs^1^

#### ^1^BC Children’s Hospital, The University of British Columbia, Vancouver, BC; ^2^Department of Pathology, Microbiology, and Immunology, Vanderbilt University Medical Center, Nashville, TN, USA; ^3^St Paul’s Hospital, The University of British Columbia, Vancouver, BC

##### **Correspondence:** Sean Duke

*Allergy, Asthma & Clinical Immunology* 2024, **20(Suppl 1):**41

**Background:** Heterozygous loss-of-function variants in *CTLA4* result in the immune dysregulation disorder termed CTLA-4 insufficiency. Clinical manifestations vary, and may include immunodeficiency, lymphocytic organ infiltration, malignancy, and autoimmunity. Diagnosing CTLA-4 insufficiency facilitates use of life-saving targeted therapies; however, can be hampered by inconclusive genetic testing results. Here we report the clinical presentations and functional validation of novel *CTLA4* variants identified at our center.

**Methods:** Patients with clinical phenotypes compatible with CTLA-4 insufficiency and a variant of uncertain significance in *CTLA4* were enrolled in the study. Variant significance was analyzed by computational analytical tools and by assessing CTLA-4-mediated transendocytosis of its ligand CD80, using plasmids expressing the variants. Clinical and immunological features were assessed before and after treatment.

**Results:** We studied 2 unrelated families with immune dysregulation and heterozygous variants in *CTLA4*. Sequencing of a 14-year-old male with a history of infections, Crohn disease, granulomatous and lymphocytic interstitial lung disease, lymphocytic infiltration of the brain, hypogammaglobulinemia, and autoimmune cytopenias revealed a de novo variant in *CTLA4* (c.424G>C, p.G142R). Targeted therapy with abatacept in combination with corticosteroids and sirolimus led to dramatic clinical improvement. Functional validation of p.G142R demonstrated impaired CTLA-4-mediated transendocytosis, confirming its pathogenicity. A 2-month-old male infant inherited a novel *CTLA4* variant (*CTLA4* c.416A>C, p.Y139S) from his father who had undergone genetic testing for a history of autoimmunity and immune deficiency. At the time of genetic diagnosis, the infant had developed autoimmune neutropenia. Both variants are absent from healthy reference populations and are predicted damaging by in silico tools.

**Conclusions:** This report identifies and characterizes 2 novel disease-causing variants in *CTLA4*, expanding the genotypic profile of CTLA-4 insufficiency. Our experience highlights the importance of considering inborn errors of immunity when evaluating patients with immune dysregulation, and the value of resolving variants of uncertain significance to confirm diagnoses and advance care.

All participants/their guardians gave written and/or oral informed consent to participate and to be published.

## 42 Additional booster doses of COVID-19 vaccine enhance neutralization efficiency against XBB.1.5

### Elsa Sakr^1^, Nisha D. Almeida^4,6^, Ian Schiller^5,6^, Danbing Ke^1^, Maria Plesa^1^, Marc-André Langlois^3^, Kaberi Dasgupta^4,5^, Bruce D. Mazer^1,2^

#### ^1^Translational Research in Respiratory Diseases, Meakins-Christie Laboratories, Research Institute of the McGill University Health Center, Montréal, QC; ^2^Department of Pediatrics, Faculty of Medicine and Health Sciences, McGill University, Montréal, QC; ^3^Department of Biochemistry, Microbiology and Immunology, Faculty of Medicine, University of Ottawa, Ottawa, ON; ^4^Department of Medicine, Faculty of Medicine and Health Sciences, McGill University, Montréal, QC; ^5^Center for Outcomes Research and Evaluation, Research Institute of the McGill University Health Center, Montréal, QC; ^6^Health Technology Assessment Unit, McGill University Health Centre, Montréal, QC

##### **Correspondence:** Elsa Sakr

*Allergy, Asthma & Clinical Immunology* 2024, **20(Suppl 1):**42

**Background:** The recent emergence of variants, including XBB.1.5, has raised concerns about the effectiveness of current vaccines. Literature has shown that a third dose of mRNA vaccines elicits spike recognition and neutralization against some Omicron subvariants, suggesting that additional doses may be necessary to maintain adequate protection against emerging variants, like XBB.1.5. In addition, it is unknown how additional doses of mRNA vaccines against the original COVID-19 strain compare to newer bivalent vaccines.

**Methods:** 44 participants in a prospective cohort provided serum samples at 28-days post-third and fourth vaccine doses. IgG anti-spike, anti-RBD and neutralizing antibodies were measured against the Wuhan strain and XBB1.5. A multivariate mixed-effects model was employed to investigate the association between several predictors and covariates, including visit, vaccine type, recent infection, age, and sex, on ID50, a measure of neutralization efficiency. Linear regression within a multivariate mixed-effects framework was used to account for clustering, since participants received multiple doses and different vaccine types.

**Results:** Our analysis shows that a fourth dose provides greater neutralizing log(ID50) compared to a third dose (p < 0.001, 95% CI [0.29, 0.78]). Moreover, there was no significant association between vaccine type and log(ID50) (p = 0.197, 95% CI [− 1.08, 0.23]), showing that receiving a bivalent vaccine as an additional booster did not give better neutralizing efficiency against XBB.1.5. A significant association was observed between recent infection, characterized by the presence of anti-N IgG, and log(ID50) (p = 0.010, 95% CI [0.16, 1.10]), proposing that a recent infection can enhance vaccine response and provide additional protection with greater neutralizing power against the XBB.1.5 variant.

**Conclusions:** Receiving additional boosters of the COVID-19 vaccine elicits the best vaccine response against XBB.1.5, while recent infection can enhance this response. However, there is no significant advantage of the bivalent over the original vaccine in neutralizing XBB.1.5.

## 43 Nociceptor neurons control vaccine-induced immunity

### Surbhi Gupta^1^, Francesco Borriello^2^, Jo-Chiao Wang^3^, Hannah Merrison^4^, Abigail J. Dutton^4^, David Dowling^2^, Clifford J. Woolf^2^, Ofer Levy^2^, Simmie Foster^4^, Sebastien Talbot^1,5^

#### ^1^Queen’s University, Kingston, ON; ^2^Children’s Hospital Boston, Boston, MA, USA; ^3^University of Montreal, Montreal, QC; ^4^Massachusetts General Hospital and Harvard Medical School, Boston, MA, USA; ^5^Karolinska Institutet, Solna, Sweden

##### **Correspondence:** Surbhi Gupta

*Allergy, Asthma & Clinical Immunology* 2024, **20(Suppl 1):**43

**Background:** Nociceptors, the sensory neurons that detect noxious stimuli and trigger pain, interact with immune cells to modulate immune responses. The nociceptor-released neuropeptide Substance P promotes B cell polarization, antibody class switching to IgE, and IgE release in models of allergic inflammation. In this study, we investigated whether nociceptors respond to vaccine adjuvants and control IgG production and clonal selection in the context of vaccination.

**Methods:** We activated and sensitized sensory neurons from mice with vaccines and adjuvants against influenza virus and pneumococcal and meningococcal bacteria in vitro and evaluated influenza vaccine-specific IgG antibody levels in mice with ablated nociceptors.

**Results:** Our results showed that sensory neurons respond to vaccines and exhibit differential activation by various noxious ligands. In mice with ablated nociceptors, IgG2c titers were reduced, while capsaicin-treated mice showed increased IgG titers.

**Conclusions:** These findings suggest a role for nociceptors in maintaining humoral immunity after vaccination. We will further explore how sensory neuron ablation or overactivation affects B-cell trafficking and antibody production in response to vaccination and pathogen challenges in mice. This research provides insights into the role of nociceptor neurons in humoral immune responses during vaccination and has implications for the development of more effective vaccines.

## 44 Interleukin-4-induced loss of smell in mice is associated with transcriptome/proteome changes suggestive of neuroinflammation and altered olfactory/calcium signaling

### Yannis Hara^1^, Mithilesh Kumar Jha^1^, Yingnan Han^2^, Hamid Mattoo^2^, Erika Martire^3^, Scott Nash^4^, Juby A. Jacob-Nara^5^, Jamie M. Orengo^6^, Alexandra Hicks^1^

#### ^1^Dupixent Research, Immunology & Inflammation, Sanofi, Cambridge, MA, USA; ^2^Precision Medicine and Computational Biology, Sanofi, Cambridge, MA, USA; ^3^Sanofi Specialty Care, Sanofi, Mississauga, ON; ^4^Medical Affairs, Regeneron Pharmaceuticals Inc., Tarrytown, NY, USA; ^5^Global Medical Affairs, Sanofi, Bridgewater, NJ, USA; ^6^Research, Allergy and Immunity, Regeneron Pharmaceuticals Inc., Tarrytown, NY, USA

##### **Correspondence:** Erika Martire

*Allergy, Asthma & Clinical Immunology* 2024, **20(Suppl 1):**44

**Background:** Dupilumab inhibits the shared interleukin (IL)-4/IL-13 receptor, IL-4Rα, and rapidly improves sense of smell and health-related quality of life in patients with chronic rhinosinusitis with nasal polyps (CRSwNP). In mice, administration of IL-4, but not IL-13, induces smell loss by Day 5, suggesting that IL-4-signaling effects play a dominant role in the restoration of smell. This study investigated the pathophysiological mechanisms of IL-4-evoked loss of smell in mice using transcriptomic and proteomic profiling.

**Methods:** Male BALB/cJ mice received intranasal administration of IL-4 and/or IL-13 daily for 5 days (Days 0–4). IL-4Rα antibody (dupilumab surrogate) was injected intraperitoneally on Days -3, 0, and 3. After treatments, the olfactory epithelium was dissected and subjected to RNA extraction for transcriptomic profiling and protein extraction for proteomic analysis. Gene set enrichment analysis (GSEA) was used to identify regulated functional pathways.

**Results:** In transcriptome analysis, IL-4, but not IL-13, upregulated genes involved in olfactory signaling (pim3, crem, creb3L1), calcium signaling (clca3b, ryr1), neuronal regeneration (ngfr, dlx3, nr4a1), and immune response (ccl8, cd163, Ly6d). In addition, IL-4, but not IL-13, downregulated genes encoding olfactory receptors (olfrs). GSEA indicated significant effects of IL-4, but not IL-13, on gene module scores for neuroimmune interactions and synaptic signaling/neuron activity. Proteomic analysis demonstrated a dominant effect of IL-4 over IL-13 on inflammatory cell recruitment to the olfactory epithelium and showed activation of neuroinflammation pathways by IL-4, but not IL-13. Finally, IL-4Rα blockade with the dupilumab surrogate restored immune homeostasis and olfactory receptors in olfactory epithelium.

**Conclusions:** IL-4 induces transcriptome and proteome changes suggestive of neuroinflammation and altered olfactory/calcium signaling in mouse olfactory epithelium. These findings provide mechanistic insight into IL-4-evoked loss of smell in mice and importance of IL4Rα signaling in smell restoration.

## 45 Real-time cytokine profiling of critically ill septic patients

### Jana Abi Rafeh^1,2^, Josie Campisi^3^, Raham Rahgoshai^3^, Nicholas Vonniessen^1,2^, Peter Goldberg^3^, Salman T. Qureshi^1,2,3^, Bruce Mazer^1,2^

#### ^1^Translational Research in Respiratory Diseases Program, McGill University Health Centre Research Institute, Montreal, QC; ^2^Meakins-Christie Laboratories, McGill University, Montreal, QC; ^3^Critical Care Program, McGill University Health Centre, Montreal, QC

##### **Correspondence:** Jana Abi Rafeh

*Allergy, Asthma & Clinical Immunology* 2024, **20(Suppl 1):**45

**Background:** Sepsis is a complex and dysregulated immune response to infection. Previous attempts to understand differences in the immune response of sepsis survivors compared to non-survivors using blood samples have not yielded consistent findings. Functional analysis of immune cells during sepsis could offer valuable prognostic information and ultimately guide treatment decisions. We are collaborating on an innovative whole blood Enzyme-Linked ImmunoSpot (ELISpot) assay with excellent dynamic range and rapid turnaround time to immunophenotype septic patients [1]. We hypothesize that this assay will identify and discriminate septic patients from critically ill non-septic patients (CINS) and that septic patients with impaired levels of TNF-α and IFN-γ may be prone to greater adverse outcomes and immune dysfunction, leading to more severe sepsis-related complications.

**Methods:** In this prospective cohort study, 50 septic patients, 30 CINS patients and 30 healthy controls will be enrolled. Serial blood samples will be collected from septic and CINS patients on specific days during their illness (days 1, 4, 7(± 1), 14(± 2), 21(± 2), and 28(± 2)). A whole blood ELISpot assay will be used to characterize the innate and adaptive immune function by quantifying TNF-α and IFN-γ-secreting cells, respectively.

**Results:** Patient recruitment for the study is ongoing. Preliminary data indicates that septic patients who do not survive show a significant and long-lasting suppression of both their innate and adaptive immune responses, in contrast to survivors and CINS patients. The adaptive response of sepsis non-survivors exhibits minimal to non-existent IFN-γ production in both CD3/CD28 stimulated and unstimulated conditions relative to survivors. Moreover, TNF-α levels of sepsis survivors reflect an exaggerated innate response to infection as compared to non-survivors.

**Conclusions:** By tracking key components of innate and adaptive immune function and correlating the results with clinical outcomes, this study will provide valuable insights into using a patient’s cytokine profile as an indicator of disease severity.

**Reference**

1. Monty B. Mazer, Charles C. Caldwell, Jodi Hanson, Daniel Mannion, Isaiah R. Turnbull, Anne Drewry, Dale Osborne, Andrew Walton, Tessa Blood, Lyle L. Moldawer, Scott Brakenridge, Kenneth E. Remy, Richard S. Hotchkiss; A Whole Blood Enzyme-Linked Immunospot Assay for Functional Immune Endotyping of Septic Patients. *J Immunol* 1 January 2021; 206 (1): 23–36.

## 46 Integrative computational analysis to identify the expression and implications of inborn errors of immunity (IEI)-related genes in cancer

### Samuel Lokanc^1^, Son Tran^1^, Ritul Sharma^1^, Pinaki Bose^1^, Nicola Wright^1^, Luis Murguia-Favela^1^, Eyal Grunebaum^2^, Aru Narendran^1^

#### ^1^University of Calgary, Calgary, AB; ^2^Hospital for Sick Children, Toronto, ON

##### **Correspondence:** Samuel Lokanc

*Allergy, Asthma & Clinical Immunology* 2024, **20(Suppl 1):**46

**Background:** The intersection of cancers and IEI related genes has been of interest to immunologists and oncologists for years. However, currently, the details of the role of IEI associated molecular alterations in the genesis and progression of cancer remain unknown. In this study we aim to identify distinct expression signatures of these genes in tumour tissues to further understand this phenomenon.

**Methods:** The TCGAbiolinks R package was used to evaluate the genomic data from 28 different tumors in the Cancer Genome Atlas database. For comparison, healthy tissue samples were incorporated from the Genotype-Tissue Expression Project (GTEx). Using this approach, we performed differential gene expression analysis using the limma-voom and edgeR pipelines through TCGAbiolinks. Each cancer type was examined with its respective healthy tissue. These genes were subjected to pathway enrichment analysis using the pathfinder R package. Examination of somatic mutations within each cancer type was also conducted. Further analysis was restricted to 472 IEI-related genes, which are curated from the Fulgent Genetics Comprehensive PID Panel.

**Results:** Our findings revealed that among the 472 IEI-related genes, 151 (32%) were upregulated and 88 (18.6%) were downregulated across all 28 TCGA datasets compared to GTEx. Cancers that had the greatest number of IEI-related genes differentially expressed were Diffuse Large B-cell Lymphoma, Testicular Germ Cell Tumours, Pancreatic Adenocarcinoma, and Skin Cutaneous Melanoma. Interestingly, genes encoding distinct complement components were predominantly downregulated in most cancers. Mutational analysis further identified a distinct IEI gene signature within individual cancer types. Kaplan–Meier survival plots identified a unique gene set that significantly stratified overall survival rates.

**Conclusions:** Our results showed that the differential expression of certain IRI-related genes may be associated with oncogenic processes and aggressive behavior in malignancies. We discuss the potential mechanisms and implications of these findings to cancer pathophysiology.

## 47 Rapid mitochondrial ROS production promotes primary NF-κB activation by controlling TRAF6 ubiquitination mechanism in macrophages

### Parinaz Tavakoli Zaniani

#### National Centre for Biotechnology (CNB-CSIC), Madrid, Spain

##### **Correspondence:** Parinaz Tavakoli Zaniani

*Allergy, Asthma & Clinical Immunology* 2024, **20(Suppl 1):**47

**Background:** Mitochondrial reactive oxygen species (mROS) play a crucial role in macrophage pro-inflammatory activation, although a detailed understanding of the mechanism and kinetics by which mROS drive signaling molecules is still lacking. Previous studies examined the late effects of mROS on TLR4 stimulation-dependent macrophage activity. In general, it is thought that NF-κB activation drives mROS and general ROS production.

**Methods:** mROS production was measured in vitro after LPS treatment with fluorescent probes. A group of inhibitors and quenchers of mROS were also investigated. In vivo mROS inhibition was examined in relation to survival and pro-inflammatory cytokine production in septic shock models. To investigate the relation between mROS and TRAF-6 ubiquitination, Western blotting and immunoprecipitation were employed.

**Results:** We performed a detailed kinetic analysis of mROS production during macrophage activation and found an unforeseen mROS generation as early as 5 min after LPS stimulation. mROS were suppressed by inhibiting or by quenching mROS intensity. Remarkably, early mROS signaling induced primary NF-κB activity and MAPK stimulation, leading to pro-inflammatory cytokine production. In contrast to the inhibitory effect of mROS suppression on NF-κB activation, inhibition of NF-κB had no effect on mROS production. In addition, our findings point for the first time to a mechanism by which mROS increase TRAF-6 ubiquitination that initiates NF-κB activity. mROS inhibition reduced LPS-induced lethality in an in vivo septic shock model by controlling pro-inflammatory cytokine production.

**Conclusions:** Overall, our results portray mROS as initiator of macrophage activation pathways and as a regulator of inflammatory responses. Taking together with previous data, our findings suggest that early mROS regulates TRAF-6 ubiquitination and initiates NF-κB activation while at later time points NF-κB activation could induce mROS production. These events support the notion that a feedback mechanism between mROS and NF-κB activation drive optimal macrophage stimulation.

## 48 Transcriptomic signature of CpG-induced regulatory B-cells is highly dependent on originating cell subset

### Eisha A. Ahmed^1^, Nicholas Vonneissen^1^, Wei Zhao^1^, Bruce D. Mazer^1,2^

#### ^1^Research Institute of the McGill University Health Centre, Montreal, QC; ^2^Division of Allergy and Immunology, Department of Pediatrics, Montreal Children’s Hospital, McGill University Health Centre, Montreal, QC

##### **Correspondence:** Eisha A. Ahmed

*Allergy, Asthma & Clinical Immunology* 2024, **20(Suppl 1):**48

**Background:** Regulatory B-cells (Bregs) are of growing interest with the capacity to supress inflammatory responses such as in asthma. Bregs are defined by expression of interleukin-10 (IL-10), however B-cell subsets which express IL-10 and their mechanism of IL-10 induction remains unclear. Toll-like receptor activation is known to induce IL-10 expression in both mouse and human B-cells, with CpG—a TLR9 agonist—commonly used to induce Bregs in vitro.

**Methods:** In this study, we use CpG to induce IL-10 expression in sorted primary B-cell subsets from naïve IL-10-eGFP reporter mice: follicular B-cells, marginal zone B-cells, and peritoneum-derived B1-cells. After 48 h of culture, B-cell subsets were sorted a second time for GFP expression, and gene expression assessed via RNA sequencing. We performed differential gene expression analysis, weighted correlation network analysis, and gene set, pathway, and gene ontology enrichment analysis to understand the similarities and differences in transcriptomes between CpG-induced Breg subsets.

**Results:** The transcriptomes of IL-10-expressing B-cell subsets differed substantially from each other, with the majority of differentially expressed genes between GFPhi and GFPlo subsets differing depending on the originating B-cell subset. Only 37 genes were identified that were differentially regulated in the same direction. Enrichment analysis revealed substantial differences between Breg subsets, though some similarities in Toll-like receptor-related pathways were identified. We further show that peritoneum-derived B1-cells express greater amounts of IL-10 compared to other Breg subsets and are characterized by a plasmablast transcriptional signature, suggesting regulatory plasmablasts may originate from B1 rather than B2 lineage.

**Conclusions:** This work highlights the necessity of deconvoluting heterogeneous cell responses to better understand mechanisms of gene regulation due to the significant differences between lymphoid and circulating B-cell populations. Since severe asthmatics have been shown to have low Bregs, understanding pathways to IL-10 production in B-cells can provide a potential novel therapeutic avenue in severe asthma.

## 49 Immunoglobulin replacement therapy in patients with immunodeficiencies—the impact of age upon drug packaging and administration preference

### Oleksandra Lepeshkina^1^, Geneviève Solomon^2^

#### ^1^Centre hospitalier Universitaire de Québec, Université Laval, Québec, QC; ^2^Association des patients immmunodéficients du Québec (APIQ), Saint-Jean sur Richelieu, Québec, QC

##### **Correspondence:** Geneviève Solomon

*Allergy, Asthma & Clinical Immunology* 2024, **20(Suppl 1):**49

**Background:** Here, the experience of young adults and adults with primary/secondary immunodeficiency receiving different combinations of subcutaneous immunoglobulin (SCIg) packaging/administration methods (i.e., vials/pre-filled syringes [PFS] with pump/manual push) were compared.

**Methods:** Responses from an incentivized online survey between Oct20–Mar21 [1] were compared by age (young adults [14–24 yrs]; adults [> 24 yrs]) and their current SCIg packaging/administration method. Questions included baseline characteristics, current and previous use of SCIg packaging/administration methods, reasons for switching (if applicable), and infusion duration (preparation, administration, and clean-up).

**Results:** In total, 28 young adults and 228 adults were using SCIg at the time of the survey. Of the SCIg packaging/administration method combinations used, PFS with pump was the most used among both young adults and adults (n = 21, 75% and n = 128, 66%, respectively; p < 0.001). Regardless of age, most respondents (> 67%) have not changed their packaging/administration method since they started using SCIg; of those that did change their SCIg packaging/administration method (young adults: n = 4, 33%; adults: n = 35, 26%). There was a trend that young adults (n = 3, 75%) switched SCIg packaging/administration method following a recommendation from a doctor/nurse, whereas most adults (n = 19, 54%) did so for ease of administration. In the PFS with pump subgroup, young adults had a longer mean (standard deviation) preparation duration compared with adults (33 [44] vs 18 [17] mins, respectively; p < 0.01), all other durations were not significantly different.

**Conclusions:** We recommend discussing all SCIg packaging/administration methods and how to optimize administration with all patients, but particularly young adults as they may be less likely to self‑advocate.

**Reference**

1. Mallick R, Solomon G, Bassett P, Zhang X, Patel P, Lepeshkina O. Immunoglobulin replacement therapy in patients with immunodeficiencies: impact of infusion method on patient-reported outcomes. Allergy Asthma Clin Immunol. 2022;18(1):110.

## 50 Functional assessment of B cell development in vitro and its potential use in CVID

### Katarzyna Farrell, Sarah M. McAlpine, Alejandro Palma, Thomas Issekutz

#### Dalhousie University, Halifax, NS

##### **Correspondence:** Katarzyna Farrell

*Allergy, Asthma & Clinical Immunology* 2024, **20(Suppl 1):**50

**Background:** Common variable immunodeficiency (CVID) is characterized by low levels of blood immunoglobulins and increased susceptibility to infections. The exact cause of CVID is not fully understood but is likely a result of genetic and environmental factors that cause primarily B cell dysfunction. Cytokines such as IL-4, IL-6, IL-10, and IL-21 have a critical role in B cell proliferation, differentiation, antibody production, isotype switching and plasma cell formation. Our objective was to develop in vitro assays using blood lymphocytes from normal donors to compare the results of normal donor B cells to those from patients with known genetic causes of CVID.

**Methods:** Blood mononuclear cells were obtained from normal donors after informed consent. The cells were cultured in vitro with various activators including CD40L, IL-4 and IL-21. After six days multiparameter flow cytometry was used to assess B cell proliferation, naïve and memory B cells, class switching and antibody secreting cells.

**Results:** CD40L, IL-4 or IL-21 alone induced little B cell activation. However, CD40L plus IL-4 strongly stimulated B cells, resulting in a 20–25 fold increase of CD19+ B cells, with a high proportion of naïve and unswitched memory B cells, and 12% switched memory B cells. The addition of IL-21 increased B cell proliferation even further, decreased the proportion of naïve and unswitched memory B cells and induced further differentiation to switched memory B cells CD27-IgD− B cells and, antibody-secreting cells. Ongoing experiments are assessing additional stimuli and examining selected CVID patients.

**Conclusions:** Blood B cells can be activated to strongly proliferate in vitro after stimulation with CD40L and cytokines in combination. IL-4 and IL-21 together can stimulate B cells to differentiate through several stages, leading to plasma cell formation. These assays should be useful in identifying the defective stage of B cell differentiation in patients with CVID.

***Katarzyna Farrell—CSACI Summer Studentship Award winner***

## 51 Multiplexed immunofluorescence imaging of tertiary lymphoid organs in the human nasal polyp

### Vitoria Murakami Olyntho^1^, Jake Colautti^1^, Manel Jordana^1^, Doron Sommer^2^, Joshua F. Koenig^1^

#### ^1^McMaster University, Hamilton, ON; ^2^Hamilton Health Sciences, Hamilton, ON

##### **Correspondence:** Vitoria Murakami Olyntho

*Allergy, Asthma & Clinical Immunology* 2024, **20(Suppl 1):**51

**Background:** Chronic rhinosinusitis (CRS) is an inflammatory condition associated with nasal congestion, difficulty breathing, pain, and can present with nasal polyps (NPs). NPs are growths of inflamed tissue populated by immune cells that may form organized lymph node-like structures called tertiary lymphoid organs (TLOs). TLOs in NPs have been associated with worse disease outcomes by exacerbating local inflammation. However, the mechanisms behind their formation, structure, and disease-perpetuating mechanisms are poorly understood.

**Methods:** NPs from three CRS patients at the McMaster University ENT clinic were processed for immunofluorescence microscopy. An iterative staining-and-bleaching technique was employed to enable multiplexed imaging on the same tissue. A 10-marker panel was designed to characterize immune and stromal cell subsets within the NP. Tissues were treated with 3,3’-diaminobenzidine to reduce non-specific eosinophil fluorescence.

**Results:** We generated the first 10-marker immunofluorescence image of the NP, allowing for the simultaneous visualization of T and B cell subsets, lymphoid tissue organizer (LTi) cells, plasma cells, germinal center B cells, stem cells, and stromal cells. TLOs were characterized as dense aggregates of CD20^+^CD45^+^ B cells surrounded by CD3^+^CD45^+^ T cells, CD4^+^CD3^−^CD45^+^ LTi cells and CD31^+^ venules. Notably, we observed a previously underappreciated diversity among TLOs. Some resembled mature TLOs with germinal center activity around CD31^+^ venules, while others formed large, dense aggregations of non-dividing B cells around seromucous glands with IL-4Rɑ^hi^ goblet cells.

**Conclusions:** We demonstrate the feasibility of high-plex immunofluorescence imaging in human NPs, having overcome eosinophil fluorescence challenges that limit imaging of this tissue. We provide preliminary evidence of TLO heterogeneity which will be pursued in future experiments. The proximity of larger TLOs to seromucous glands suggests a potential mechanism for lymphocyte recruitment in response to environmental antigens, possibly influencing NP development. These findings apply to other diseases involving TLOs, including cancer, autoimmune disorders, and other inflammatory conditions.

***Vitoria Murakami Olyntho—CSACI Summer Studentship Award winner***

## Case reports

## 52 Anaphylaxis due to a pet guinea pig bite

### Abdulrahman Al Ghamdi^1,3^, Stephen Betschel^2^

#### ^1^The Hospital for Sick Children, Toronto, ON; ^2^St. Michael’s Hospital, Toronto, ON; ^3^King Faisal Specialist Hospital and Research Center, Jeddah, Saudi Arabia

##### **Correspondence:** Abdulrahman Al Ghamdi

*Allergy, Asthma & Clinical Immunology* 2024, **20(Suppl 1):**52

**Background:** Anaphylaxis due to rodent bites is a rare occurrence. Guinea pigs are a species of rodents that have been popular as pets and in research laboratories. There have been no reported cases of anaphylaxis due to guinea pig bites.

**Case presentation:** An 18-year-old male acquired guinea pigs as pets when they were newly born. A few months later, he started noting nasal pruritus and rhinorrhea when he was in contact with the guinea pigs. 18 months after acquiring the guinea pigs one of them bit him on the left index finger. He immediately started to have rhinorrhea, nasal congestion and pruritus then he started to develop pharyngeal pruritus and a feeling of tightness in the upper airway. His symptoms progressed to difficulty breathing and he also vomited twice. He had no skin rash and no cardiovascular symptoms. He was taken to his place of work (a restaurant) that was stocked with epinephrine auto-injectors and epinephrine was administered about 30 min after the bite. His symptoms resolved completely within a few minutes. He was taken by ambulance to the ER, his first documented vital signs were 4 h after the incident and were all within normal. He was discharged home after a short period of observation.

There were no other possible triggers of his reaction and no noted co-factors for a more severe reaction. When he presented for evaluation, he was well. Skin prick testing showed a 0 mm wheal to normal saline, 3 mm wheal to histamine, and a 7 mm wheal to guinea pig epithelium extract. Specific IgE to guinea pig epithelium was 8.05 kU/L.

**Conclusions:** Our patient’s history and investigations are in keeping with a diagnosis of anaphylaxis due to the bite of a guinea pig and provides further evidence of the risk of anaphylaxis when dealing with rodents.

All participants/their guardians gave written and/or oral informed consent to participate and to be published.

## 53 Cannabis-fruit/vegetable syndrome: an unusual case without pollen co-sensitization

### Joanne Wang, Gofrey Lam, Raymond Mak

#### University of British Columbia, Vancouver, BC

##### **Correspondence:** Joanne Wang

*Allergy, Asthma & Clinical Immunology* 2024, **20(Suppl 1):**53

**Background:** Cannabis use has become increasingly popular since its legalization. In 2022, 19% of Canadians over 16 years reporting use over the past 30 days. Cannabis is associated with an extensive spectrum of cross-reactivity with fruits and vegetables through a phenomenon known as cannabis-fruit/vegetable syndrome. While most patients are co-sensitized with cross-reactive pollen, we present a unique case of cannabis-fruit/vegetable syndrome without birch pollen co-sensitization.

**Case presentation:** Since 2021, a 26-year-old female with intermittent cannabis smoking began noticing IgE mediated symptoms when eating previously tolerated fruits within the birch pollen family. Her first instance was with fresh cherries where she instantly experienced ocular/throat pruritus and generalized urticaria. In 2022, she had similar reactions to fresh peaches and raspberries. She also began experiencing immediate ocular/throat pruritus with Cannabis sativa but not with Cannabis indica. Her fresh fruit skin test was positive for nectarine (10 mm), plum (6 mm), raspberry (12 mm), blackberry (6 mm), and both Cannabis sativa (7 mm) and indica (11 mm). Her environmental panel was negative to common grass, tree and weed pollens. She was prescribed an epinephrine autoinjector given her systemic symptoms.

**Conclusions:** Multiple potential allergens including non-specific lipid transfer proteins (nsLTP), thaumatin-like protein, ribulose-1,5-bisphosphate carboxylase oxygenase, and oxygen evolving enhancer protein are thought to be contributors to cannabis allergies. Of these, nsLTP is a pan-allergen found ubiquitously throughout the plant kingdom, potentially explaining cross activities between cannabis, fruits, and vegetables. Our case of cannabis-fruit/vegetable syndrome in an otherwise healthy individual is interesting as her skin testing showed no reaction against common pollens, specifically birch, a well-known aeroallergen to cross-react with cherries, peaches, and plums. These findings suggest the patient became primary sensitized through cannabis. With increasing cannabis accessibility, more research is needed to study not only health implications, but also its culprit allergens for more widespread clinical testing and treatment in upcoming years.

All participants/their guardians gave written and/or oral informed consent to participate and to be published.

## 54 A rare case of transfer of Shellfish allergy from allergic donor to anergic recipient in Solitary Renal Transplant

### Jung Min (Julie) Hong, Lundy McKibbin

#### University of Manitoba Department of Internal Medicine, Winnipeg, MB

##### **Correspondence:** Jung Min (Julie) Hong

*Allergy, Asthma & Clinical Immunology* 2024, **20(Suppl 1):**54

**Background:** Since the first case of transplant acquired food allergy (TAFA) reported in the 1990s, TAFA has been increasingly recognized in solid organs transplantation. Due to the concern of anaphylaxis, acquiring food allergy significantly impact patients’ lives. There have been many cases of IgE mediated food allergy transmission post-solid organ transplant most predominantly in pediatric liver transplant recipients. While such transmissions have been reported in lung, heart and pancreatic transplant, TAFA in solitary renal transplants are very rare.

**Case presentation:** Here we report a case of 61-year-old female with history of end stage renal disease secondary to diabetic nephropathy who underwent recent solitary renal transplant two months prior to presentation to the allergy clinic. She had known childhood history of dust mite sensitization without clinical symptoms but had no IgE mediated hypersensitivity to shellfish prior to transplant. She was started on Tacrolimus, Mycophenolate Mofetil and prednisone after transplant for immunosuppression. It was disclosed to her that the donor had serious shellfish allergy and a question was raised whether or not allergy could be transferred to the recipient. Due to abundance of caution, she avoided shellfish until she could be evaluated in the allergy clinic. Interestingly, percutaneous skin testing revealed immediate hypersensitivity to shellfish believed to be acquired post transplantation. Further testing revealed elevated shellfish specific IgE level in her serum. Oral challenge was not pursued as intradermal skin testing revealed large positive reaction with a wheal diameter of 12 mm to fresh shrimp.

**Conclusions:** Based on the paucity of published cases, incidences of TAFA in solitary renal transplant patients are exceedingly rare. Due to the serious consequences of allergic reaction, this raises the importance of disclosure of allergic history as part of transplant screening, as well as, implementing appropriate protocol and patient education to reduce the risk to the recipient.

All participants/their guardians gave written and/or oral informed consent to participate and to be published.

## 55 Recurrent pre-menstrual expression of contact hypersensitivity reactions in a woman sensitized to nickel

### Maggie Jiang, Peter Vadas

#### Division of Allergy and Clinical Immunology, St. Michael’s Hospital, University of Toronto, Toronto, ON

##### **Correspondence:** Maggie Jiang

*Allergy, Asthma & Clinical Immunology* 2024, **20(Suppl 1):**55

**Background:** It is recognized that women may be more prone to immediate hypersensitivity reactions in the few days leading up to the start of menses. This can occur with exposure to exogenous allergens (i.e., anaphylaxis to peanut during OIT) or endogenous allergens (i.e. catamenial anaphylaxis). Here, we report a case of cyclical contact hypersensitivity to nickel which recurs only pre-menstrually.

**Case presentation:** We describe a 30-year-old woman who presented with a 2-year history of recurrent periumbilical dermatitis for the 2–7 days prior to the onset of menses. She had been referred for assessment of suspected autoimmune progesterone dermatitis due to the timing of symptoms. Dermatitis never involved areas outside of the periumbilical area, where she wore an umbilical piercing made with costume jewelry. Skin testing done to progesterone 50 mg/mL was negative, with negative saline control and positive histamine control. Patch tests applied in duplicate to nickel, chromate and cobalt from the North American panel were strongly positive at 72 h to nickel. She was advised to avoid further nickel jewelry exposure and to remove the umbilical piercing. With these precautions, she noted complete resolution of further periumbilical dermatitis during subsequent menstrual cycles.

**Conclusions:** There are a number of considerations in the investigation of premenstrual reactions including autoimmune progesterone dermatitis, adverse reactions to medications taken pre-menstrually, and foods ingested at that time. This case highlights another important consideration: premenstrual accentuation of pre-existing allergic contact dermatitis. Clues suggesting contact dermatitis over other premenstrual conditions include specific, reproducible areas of reactions in atypical patterns, and known exposure to common contact allergens such as nickel. Thorough evaluation includes the use of both patch testing and progesterone skin testing in order to help narrow the differential diagnosis.

## 56 Wasp-sting induced takotsubo cardiomyopathy

### Karl Maxemous, Noah Lewis, Samira Jeimy, Kara Robertson, Patrick Teefy

#### Western University, London, ON

##### **Correspondence:** Karl Maxemous

*Allergy, Asthma & Clinical Immunology* 2024, **20(Suppl 1):**56

**Background:** Takotsubo cardiomyopathy (TCM) is characterized by reversible left ventricular remodeling, triggered by emotional or physical stress leading to a surge in catecholamines. While insect stings can induce adrenaline surges, its association with TCM is rare. To our knowledge, this is the first reported case in Canada.

**Case presentation:** A 68-year-old woman with no known comorbidities presented to the emergency department (ED) with worsening chest pain, left arm weakness and dizziness after being stung by a paper wasp. She developed localized swelling and erythema immediately after the sting, followed by pruritus, exertional dyspnea and dizziness within 24 h. In the ED, she received treatment with prednisone, topical hydrocortisone, cetirizine and then was discharged. She returned 24 h later with chest pain, tachycardia, and diaphoresis. Cardiac investigations revealed T-wave inversions, elevated troponin-I and D-dimer levels and imaging ruled out pulmonary embolism. Echocardiography showed reduced ejection fraction, an apical thrombus, and an apical wall-motion abnormality. Cardiac catheterization excluded coronary artery disease. She was diagnosed with Takutsobu cardiomyopathy, presumably related to the catecholamine surge from the sting reaction. She was started on medical management with a beta blocker and angiotensin receptor blocker (ARB).

**Conclusions:** Allergic reactions can be associated with cardiac manifestations, for example Kounis syndrome. In this case, a stinging insect reaction was associated with Takotsubo cardiomyopathy. This case raises clinical dilemmas regarding the use of epinephrine autoinjectors and venom immunotherapy (VIT) in patients with cardiac sequelae and a previous epinephrine surge. After shared decision making around the risks, a modified protocol for venom desensitization was chosen while also considering the patient’s cardioprotective medications. The patient tolerated the VIT buildup phase well, thus far, and her anxiety related to venom has significantly decreased.

All participants/their guardians gave written and/or oral informed consent to participate and to be published.

## 57 Anaphylaxis to leech bite: a case report with exploration of cross-sensitization and protein homologies

### Maggie Jiang^1^, Thomas Eiwegger^2^, Sebastian Kvist^3^, Gabriele Gadermaier^4^, Carmen Li^2^, Jennifer Hoang^2^, Peter Vadas^1^

#### ^1^Division of Allergy and Clinical Immunology, St. Michael’s Hospital, University of Toronto, Toronto, ON; ^2^Division of Clinical Immunology and Allergy, The Hospital for Sick Children, University of Toronto, Toronto, ON; ^3^Collection and Research, Museum of Natural History, Stockholm, Sweden; ^4^Department of Biosciences, University of Salzburg, Salzburg, Austria

##### **Correspondence:** Maggie Jiang

*Allergy, Asthma & Clinical Immunology* 2024, **20(Suppl 1):**57

**Background:** Various types of allergic reactions to leech bite have been reported including local reactions, type IV hypersensitivity reactions, and anaphylaxis [1]. However, there are no published studies that have characterized the allergenic components of leech bite or examined patterns of cross-reactivity [1].

**Case Presentation:** We describe a 10-year-old boy with a past history of eczema and delayed local reactions to leech bite, who developed anaphylaxis to a bite from a freshwater leech in southern Ontario. Skin prick testing to mashed frozen leech (placobdella rugosa and macrobdella decora) collected from the lake was positive and the patient also developed malaise and lightheadedness requiring administration of IM epinephrine × 1. Two years later, he also developed anaphylaxis to a hymenoptera sting, with venom skin testing positive to white faced hornet, paper wasp, yellow hornet, and yellow jacket. Baseline serum tryptase was normal on two occasions (4.1 and 4.0 ng/mL). Specific IgE testing using ALEX chip assay was positive to American house dust mite, European house dust mite, paper wasp, yellow jacket, common mussel, house cricket, migratory locust, and mealworm. Fresh leech from the lake was collected and protein was extracted and identified using Western blots and mass spectrometry. Several proteins were identified, with a number sharing homologies with dust mite, finned fish, and shellfish.

**Conclusions:** This is the first attempt at characterizing leech salivary proteins and characterizing patterns of cross-reactivity. Basophil activation testing is in progress to characterize major leech salivary proteins causing anaphylaxis as well as exploration of possible cross reactivity between leech and hymenoptera venom.

All participants/their guardians gave written and/or oral informed consent to participate and to be published.

**Reference**

1. Pourrahimi M, Abdi M, Ghods R. Complications of leech therapy. Avicenna Journal of phytomedicine. 2020 May;10(3):222.

## 58 Sialodochitis fibrinosa managed with chronic steroid and subcutaneous immunotherapy

### Ran Huo^1^, Juan Ruiz^2^

#### ^1^University of Calgary, Calgary, AB; ^2^Clinical Instructor, University of British Columbia, Vancouver, BC

##### **Correspondence:** Ran Huo

*Allergy, Asthma & Clinical Immunology* 2024, **20(Suppl 1):**58

**Background:** Sialodochitis fibrinosa, or Kussmaul disease, is a rare condition of obstruction of salivary ducts by mucofibrinous plugs, causing recurring swelling and pain in major salivary glands, primarily in middle-aged Japanese females. While the exact etiology remains unknown, there is a correlation with allergic background, with symptoms including gland swelling, discharge of eosinophil-rich plugs, elevated IgE levels, and dilated salivary ducts on imaging. Treatment involves compressive massages of the salivary glands, antihistamines, corticosteroids, and in severe cases, instrumental dilation of the ducts. Anti-IL5 therapy shows potential due to the high levels of salivary IL5 observed in patients.

**Case presentation:** A 43-year-old Japanese female with atopic family history and past medical history including breast cancer, asthma, and allergic rhinitis presents with over 10 years of swelling and pain in all four major salivary glands, accompanied by excessive mucus. Standard skin prick testing indicates allergies to dust, cockroach, cat, and dog. In 2020, the patient was diagnosed with sialodochitis fibrinosa based on salivary gland biopsy. Stained samples of the mucous material demonstrate eosinophils. IgE level was normal. Various therapies have been attempted, including endoscopy dilation and stenting of the ducts, antihistamines, massage, without much success. Chronic treatment with prednisone at a daily dose of 5 mg, increased to 20 mg during flares, has decreased symptom burden. In 2022, the patient was initiated on subcutaneous immunotherapy (SCIT) targeting dust, cat, and dog for the off-label treatment of sialodochitis fibrinosa, currently with good treatment response. If SCIT fails, the next step would be to consider anti-IL5 therapy.

**Conclusions:** When encountering patients with recurrent swelling of major salivary glands, it is important to consider sialodochitis fibrinosa as a potential diagnosis, particularly in individuals with atopic background, elevated eosinophils, and IgE levels. This case highlights the successful off-label use of SCIT alongside chronic steroid treatment for sialodochitis fibrinosa.

All participants/their guardians gave written and/or oral informed consent to participate and to be published.

## 59 Refractory anti-IFN-γ autoantibody-associated immunodeficiency syndrome

### Ran Huo^1^, Juan Ruiz^2^

#### ^1^University of Calgary, Calgary, AB; ^2^University of British Columbia, Vancouver, BC

##### **Correspondence:** Ran Huo

*Allergy, Asthma & Clinical Immunology* 2024, **20(Suppl 1):**59

**Background:** Interferon-gamma (IFN-γ) plays a crucial role in connecting myeloid and lymphoid immune pathways, which is essential for controlling intracellular infections. The presence of anti-IFN-γ autoantibodies has been linked to various opportunistic infections such as disseminated nontuberculous mycobacterium, and is predominantly seen in individuals of Southeast Asian descent. Antimicrobial therapy typically fails, and many will require immunosuppression. There is no established standard treatment for anti-IFN-γ autoantibody-associated immunodeficiency syndrome. Rituximab is a monoclonal antibody against CD20 on B cells and leads to a significant reduction in anti-IFN-γ autoantibodies levels.

**Case presentation:** A 58-year-old Filipino male experienced multiple opportunistic infections since 2017, including *Salmonella*, disseminated *Mycobacterium avium complex (MAC)*, and *Mycobacterium abscessus*. He had a high titer of anti-IFN-γ autoantibodies and was diagnosed with anti-IFN-γ autoantibody associated immunodeficiency syndrome. In 2020, he received three doses of Rituximab therapy. In 2021, he had breakthrough infections: *Aeromonas gastroenteritis* and *Helicobacter pylori*, and his IFN-γ release assay test showed no mitogen stimulation. In February 2022, he initiated a second course of Rituximab at a weekly dosage of 700 mg (375 mg/m^2^) for four weeks, followed by maintenance doses every eight weeks for a total of eight doses. In May 2022, he experienced an adverse drug reaction (palmar pustulosis and delayed hypotension) to Rituximab, prompting a split dose of 300 mg/400 mg administered over two days and premedication with prednisone. One year later, he remains infection-free.

**Conclusions:** We present a case of refractory anti- IFN-γ autoantibody-associated immunodeficiency syndrome treated with Rituximab. Rituximab is usually given at 375 mg/m^2^ weekly for four doses and then at wider intervals, with a total of four to 18 doses, however, there is no standardized protocol. Our case report provides additional insight into the duration and dosage of Rituximab therapy. Clinicians should suspect anti-IFN-γ autoantibody in Southeast Asian patients who develop severe opportunistic infections.

All participants/their guardians gave written and/or oral informed consent to participate and to be published.

## 60 Suspected drug-induced Sweet’s syndrome in a patient receiving allopurinol and hydralazine

### Madelaine P. Beckett, Arun Dhir

#### University of British Columbia, Vancouver, BC

##### **Correspondence:** Arun Dhir

*Allergy, Asthma & Clinical Immunology* 2024, **20(Suppl 1):**60

**Background:** Sweet’s syndrome was originally described in 1964 as an acute inflammatory skin eruption with fever and leukocytosis [1]. A bullous variant of Sweet’s syndrome has also been described, which is more commonly associated with malignancy [2]. Multiple medications have been associated with a drug-induced form of Sweet’s syndrome [2, 3].

**Case presentation:** An 81-year-old woman with crystalline arthritis began allopurinol alongside ongoing methotrexate treatment. She had been on long-term hydralazine for hypertension. Two weeks later, she developed a flare of arthritis and hand edema and started a short course prednisone taper. Concurrently, she was admitted to the hospital for diverticulitis. On admission, prednisone was discontinued. The patient subsequently developed violaceous, edematous plaques and bullae on the extremities. She also had conjunctivitis, chemosis, and mucositis. The patient’s vitals were within normal limits. Bloodwork showed leukocytosis with neutrophilia (peak 13.3 × 10^9^ cells/L) but no eosinophilia. Creatinine peaked at 203 umol/L from baseline, while liver enzymes (ALT 171 U/L, ALP 360 U/L, GGT 166 U/L) were elevated with normal bilirubin. The CRP reached a maximum of 260 mg/L. Abdominal ultrasound was normal, and blood cultures were negative. HLA-B5801 testing was negative. Skin biopsies showed neutrophilic infiltrates suggestive of Sweet’s syndrome. Malignancy screens were up-to-date with normal SPEP results. Methotrexate was stopped due to mucositis. The patient was restarted on prednisone at 50 mg daily, gradually tapering. The patient also received intravenous hydration. Renal and hepatic abnormalities resolved with treatment. Presently, the patient is being monitored for recurrence while on tapering prednisone.

**Conclusions:** Drug-induced Sweet’s syndrome is an unusual hypersensitivity reaction. Previous reports have associated hydralazine [4] and allopurinol [5] with similar reactions. Although uncommon, bullous lesions can be present. Along with other severe adverse cutaneous reactions, clinicians should consider drug-induced Sweet’s syndrome in cases of multi-organ blistering drug reactions.

All participants/their guardians gave written and/or oral informed consent to participate and to be published.

## 61 Inflammation of actinic keratoses induced by subcutaneous immunoglobulin therapy

### Arun Dhir^1^, Catherine Biggs^2^, Persia Pourshahnazari^1^

#### ^1^Division of Adult Allergy and Immunology, Department of Medicine, University of British Columbia, Vancouver, BC; ^2^Division of Immunology, Department of Pediatrics, University of British Columbia, Vancouver, BC

##### **Correspondence:** Arun Dhir

*Allergy, Asthma & Clinical Immunology* 2024, **20(Suppl 1):**61

**Background:** Actinic keratoses (AK) are common erythematous papules with scale that occur on sun-damaged skin and may progress to squamous cell carcinoma. Inflammation of actinic keratosis has been reported as a side effect of systemic chemotherapies. Cutaneous reactions (e.g. urticaria, injection site reactions) have been associated with immunoglobulin replacement therapy, including subcutaneous immunoglobulin (SCIG). However, inflammation of actinic keratoses due to immunoglobulin therapy has not been previously reported.

**Case presentation:** A 76-year-old male with MPO-positive ANCA-associated vasculitis developed hypogammaglobulinemia secondary to rituximab. Medical history included chronic obstructive pulmonary disease and actinic keratoses with prior SCC resection. Recurrent respiratory infections and low IgG levels prompted SCIG therapy at 0.1 g/kg/week. At this time, the patient was tapering off of prednisone. No other medications were started. Eight weeks into SCIG therapy, and near the end of the prednisone taper, the patient developed a diffuse eruption of erythematous, pruritic papules and plaques. Bloodwork showed no eosinophilia or liver/kidney dysfunction, though CRP was elevated at 9.8 mg/L. Given the history of vasculitis, prednisone was initiated empirically at 20 mg/day with a 5 mg/week taper. Topical betamethasone 0.1% ointment and oral bilastine were also given. As immunoglobulin levels were robust, SCIG therapy was temporarily halted. Dermatology performed a skin biopsy, suggesting actinic keratoses without signs of a drug reaction. The lesions were thus thought to be inflamed actinic keratoses. The patient’s condition improved within a week. Gradual resumption of SCIG therapy with close monitoring is planned given potential recurrence.

**Conclusions:** Inflammation of actinic keratoses induced by SCIG is a novel phenomenon. Our patient was successfully treated with oral and topical steroids, with a plan to gradually restart SCIG therapy. This may represent a rare reaction due to immune system reconstitution, though the specific mechanism is unclear.

All participants/their guardians gave written and/or oral informed consent to participate and to be published.

## 62 A rare presentation of acquired angioedema treated with lanadelumab

### Karl Maxemous, Rongbo Zhu

#### Western University, London, ON

##### **Correspondence:** Karl Maxemous

*Allergy, Asthma & Clinical Immunology* 2024, **20(Suppl 1):**62

**Background:** Acquired angioedema (AAE) is a rare cause of bradykinin mediated angioedema and can be life-threatening. It is non-hereditary, presents later in life and can be associated with lymphoproliferative disorders and autoimmunity. No established guidelines exist on the treatment of this condition. We describe a case of a patient who developed AAE and was successfully treated with lanadelumab.

**Case presentation:** A 66-year-old male with chronic lymphocytic leukemia was referred for new onset episodes of angioedema. Initially, he presented with lip, tongue and eyelid angioedema in the Emergency Department (ED) and was treated as presumed cellulitis. The second episode involved lip and eyelid angioedema, exacerbated by ibuprofen. He went to the ED, where he was prescribed diphenhydramine, prednisone, and epinephrine, with resolution of symptoms 48 h later.

Initial investigations showed a low C4 of 0.05 (0.10–0.40 g/L) with a normal C1 inhibitor level of 0.28 (0.21–0.38 g/L) and function of 0.84 (0.75–1.59 U/mL). In subsequent months, following a dental procedure, the patient had significant laryngeal swelling requiring ICU admission. C4 and C1 inhibitor level drawn during this episode were both low. The patient had repeat blood work in clinic, demonstrating persistently low levels of C4 and C1 inhibitor level. Subsequent C1q performed was undetectable.

The patient was diagnosed with AAE and with collaborative decision-making was put on lanadelumab as long term prophylaxis and icatibant for acute therapy. Following initiation of lanadelumab, the patient had improvement of his angioedema episodes and overall quality of life (AE-QoL score from 47 to 25).

**Conclusions:** This case highlights a rare cause of angioedema and encompasses the need to perform sequential testing, especially during acute attacks. Currently, treatment options for AAE are limited to case series. Overall, this case underscores the importance of creating guidelines surrounding AAE, similar to current guidelines for hereditary angioedema.

All participants/their guardians gave written and/or oral informed consent to participate and to be published.

## 63 Tomatillo allergy in a patient with pollen-food allergy syndrome

### Arun Dhir, Godfrey Lam

#### Allergy and Immunology, Department of Medicine, University of British Columbia, Vancouver, BC

##### **Correspondence:** Arun Dhir

*Allergy, Asthma & Clinical Immunology* 2024, **20(Suppl 1):**63

**Background:** Tomatillos are a member of the nightshade family and are commonly used in Mexican cuisine. Though related to tomatoes, they are a distinct fruit. Tomatillos are a rare food allergen with few cases reported in the literature.

**Case presentation:** A 33-year-old woman with a medical history of allergic rhinitis and experienced intermittent oral pruritus when consuming raw fruits, vegetables, peanuts, and tree nuts, with occasional lip swelling. Cooking food alleviated her symptoms.

Previously, the patient had an immediate oral pruritus reaction to raw tomatillos in salsa. Thirty minutes later, she developed progressive abdominal pain. She took ibuprofen with no relief. Approximately 4 h later, as her abdominal pain was ongoing, she presented to an emergency department. Subsequently, she developed systemic urticaria and dyspnea, promptly treated with epinephrine with instant relief. Following this event, the patient avoided tomatillos but consumed tomatoes and used NSAIDs without experiencing any adverse reactions. She had another reaction to a meal containing raw tomatillos, developing oral pruritus and abdominal pain one hour later, improving with diphenhydramine.

Skin testing was positive to birch and hazelnut extracts (4 mm) but negative to tomato extract, peanuts, and other tree nuts. Fresh food skin testing was positive to tomatillo (12 mm) but negative to tomato. Subsequently, the patient received a diagnosis of tomatillo allergy concurrent with a pollen-food allergy syndrome involving other birch cross-reactive foods. The patient was advised to strictly avoid tomatillos and to carry an epinephrine autoinjector. In the absence of systemic symptoms, continued cautious consumption of birch cross-reactive foods was permitted.

**Conclusions:** Tomatillos are a rare food allergen. As commercial extracts are not available, fresh food testing is required to confirm the diagnosis. Cross-reactivity with tomatoes may not be present. Further studies are necessary to identify the underlying allergenic protein components.

All participants/their guardians gave written and/or oral informed consent to participate and to be published.

## 64 An unexpected culprit of delayed drug hypersensitivity reaction

### Erika Y. Lee^1,2^, Jackie Campbell^1^, Jonathan Zipursky^3^

#### ^1^Drug Safety Clinic, Sunnybrook Health Sciences Centre, Toronto, ON; ^2^Division of Clinical Immunology & Allergy, Department of Medicine, University of Toronto, Toronto, ON; ^3^Division of Clinical Pharmacology & Toxicology, Sunnybrook Health Sciences Centre, Toronto, ON

##### **Correspondence:** Erika Y. Lee

*Allergy, Asthma & Clinical Immunology* 2024, **20(Suppl 1):**64

**Background:** Delayed drug hypersensitivity reactions (DHR) are T-cell mediated with prominent skin findings that usually manifest days to weeks after drug exposure. Drug allergy testing can assist with the diagnosis and confirmation of the culprit drug. Common testing for delayed DHR includes patch testing and intradermal testing (IDT), sometimes followed by drug provocation testing (DPT).

**Case presentation:** A 37-year-old woman was referred for a suspected labetalol allergy. She was started on oral labetalol in the third trimester for gestational hypertension. After approximately 6 weeks on the drug, she noticed a rash in her groin. On the following day, she underwent an urgent caesarian delivery and received Penicillin G and oxytocin. In the ensuing days, she developed a generalized morbilliform rash, fever and facial swelling, and was subsequently readmitted to the hospital. Laboratory investigations showed no evidence of peripheral eosinophilia nor internal organ involvement. Labetalol, which was suspected to be the culprit due to the continuous exposure and timing of the reaction, was discontinued, and the rash improved in a week.

In clinic, we performed IDT to labetalol (0.05 mg/mL and 0.5 mg/mL), penicillin G (10,000 U/mL), ampicillin (20 mg/mL), and Pre-Pen. The patient had negative IDT to labetalol at immediate and delayed readings. The IDT to penicillins was negative at 20 min but became positive to ampicillin and penicillin G at 24 h. She later tolerated an oral challenge to labetalol. She was therefore diagnosed with penicillin allergy and counselled to avoid penicillins.

**Conclusions:** Despite a clinical history suggestive of a delayed DHR to labetalol, our skin testing identified penicillin as the actual culprit. The case illustrates the role of drug allergy testing to assist with DHR diagnosis and highlights the importance of referring patients to a drug allergy clinic to identify potential culprits and verify relevant drug allergies.

All participants/their guardians gave written and/or oral informed consent to participate and to be published.

## 65 Utilizing a ladder approach for the management of peanut food protein-induced enterocolitis syndrome (FPIES)

### Nicole A. Mar, Edmond S. Chan, Stephanie C. Erdle

#### Division of Allergy, Department of Pediatrics, University of British Columbia, BC Children’s Hospital, Vancouver, BC

##### **Correspondence:** Nicole A. Mar

*Allergy, Asthma & Clinical Immunology* 2024, **20(Suppl 1):**65

**Background:** Current management of food protein-induced enterocolitis syndrome (FPIES) can be burdensome as it involves strict avoidance and a resource-intensive oral food challenge (OFC) when older. While the use of ladders has been successful in treating milder IgE-mediated cow’s milk and egg allergies, there is limited evidence for its use in the management of FPIES to foods such as peanut. We present two infants with FPIES to both egg and peanut, who after being managed with the Canadian Egg Ladder were successfully started on a similar ladder approach to peanut.

**Case presentation:** Case 1: 6-month-old male with several episodes of severe vomiting, diarrhea, and lethargy 2 h after ingestion of egg. At 8-months, multiple episodes of projectile vomiting 2–3 h after ingestion of peanut. Skin prick test (SPT) was mildly positive to egg and negative to peanuts. Patient successfully completed the egg ladder by 2-years but repeat trials of peanut every few months still caused delayed vomiting. Recently was successfully started on a peanut ladder.

Case 2: 6-month-old male with 3 episodes of severe vomiting 2 h after ingestion of egg, and 2 episodes of profuse vomiting 2–3 h after ingestion of peanut. SPT negative to egg and peanut. Successfully completed both the egg ladder and peanut ladder by 1-year follow-up.

**Conclusions:** These two cases of egg and peanut FPIES managed with a ladder approach reduce/eliminate the need for burdensome exit OFCs, and are a safe and effective alternative to strict avoidance. Additionally, this approach could potentially prevent the development of IgE-mediated allergy by allowing earlier and continued introduction of allergens. Larger studies assessing the use of a ladder approach for the management of FPIES to foods other than cow’s milk and egg should be considered.

All participants/their guardians gave written and/or oral informed consent to participate and to be published.

## 66 Bradykinin-induced angioedema triggered by venom immunotherapy: a case report

### Junghoon Ko, Juan C. Ruiz

#### University of British Columbia, Vancouver, BC

##### **Correspondence:** Junghoon Ko

*Allergy, Asthma & Clinical Immunology* 2024, **20(Suppl 1):**66

**Background:** Bradykinin-induced angioedema is often classified into three types: hereditary (HAE), acquired, and drug-induced. HAE involves decreased production or function (Type I vs II) of the C1 esterase inhibitor (C1-INH) protein responsible for breaking down bradykinin whereas the acquired form is associated with overconsumption of C1-INH. Clinically, while both HAE and acquired angioedema are characterized by cutaneous, respiratory and/or gastrointestinal manifestations, HAE tends to present earlier, usually in adolescence, and acquired angioedema is often associated with other lymphoproliferative or autoimmune disorders. Common triggers for both include stress, trauma, and infection.

**Case presentation:** Here, we present a 61-year old male who reported 2 episodes of severe facial angioedema within 48–72 h after Venom immunotherapy (VIT). On both occasions, he described asymmetrical tongue swelling following VIT which was not relieved by 80 mg of bilastine and lasting for approximately 1–2 days. Interestingly, he had not had an adverse reaction during the first 11 months of VIT and denied any symptoms of infection, trauma or use of suspicious medications such as ACE-inhibitors. Laboratory tests demonstrated decreased C1-INH function and C4 level, during both events. While the later age of onset was initially suggestive of acquired angioedema, this patient had no signs or symptoms of lymphoma or autoimmune disease and had an unremarkable serum protein electrophoresis (SPEP). Moreover, his sister had an episode of angioedema in her childhood requiring a tracheostomy, suggesting a genetic component and shifting the provisional diagnosis to HAE Type II.

**Conclusions:** We are currently awaiting further labs to rule out acquired angioedema. The patient’s family members have also been asked to undergo testing for HAE. Regardless of the ultimate diagnosis, this report outlines an unusual case of bradykinin-induced angioedema triggered by VIT. The patient continues to be followed monthly for VIT at a reduced dose while we secure Icatibant for management of acute attacks.

All participants/their guardians gave written and/or oral informed consent to participate and to be published.

## 67 A case report of deer meat anaphylaxis in a child

### Vicky Le Blanc, Mary M. McHenry

#### Dalhousie University, Department of Paediatrics, Division of Allergy, Halifax, NS

##### **Correspondence:** Vicky Le Blanc

*Allergy, Asthma & Clinical Immunology* 2024, **20(Suppl 1):**67

**Background:** Allergic reactions to deer meat is known in the context of the alpha-gal syndrome. Patients typically react to other mammalian meats such as beef, lamb and pork. Reactions are delayed, occurring a few hours after ingestion. Ticks, such as lone star tick, has been identified as a cause of sensitization to the alpha-gal antigen. In contrast, no case of IgE-mediated allergy to deer meat has been reported to our knowledge.

**Case presentation:** We describe a case of an 18-month-old girl with anaphylaxis following ingestion of deer meat. She is an atopic child with personal history of mild atopic dermatitis and multiple food allergies (milk, peanut and shellfish). She is on peanut oral immunotherapy and on the Canadian milk ladder, which she tolerates both well. After the first exposure to deer meat around 10 months of age, she developed urticaria and emesis within minutes of ingestion. Parents were suspicious for cross contamination with shellfish as the pan used to cook the deer meat was previously used to cook shellfish. Accordingly, deer meat was not avoided. On second ingestion, she developed a very similar reaction. Parents were then suspicious that butter might have been used during the preparation. Finally, on third exposure, great caution was used during the preparation of the meat to avoid any cross contamination with her known allergens. Despite this, she developed the same symptoms with immediate hives and vomiting. She tolerates other mammalian meats such as beef, pork and lamb. She has never been bit by a tick. Skin testing was positive to cooked deer meat (5 mm), with a positive histamine and negative saline control. She continues to avoid deer meat and carry an epinephrine auto-injector at all times.

**Conclusions:** Anaphylaxis to mammalian meats outside the context of alpha-gal syndrome is very rare but recognition is important.

All participants/their guardians gave written and/or oral informed consent to participate and to be published.

## 68 Case report: new onset Bullous Pemphigoid (BP) following 5th COVID-19 vaccination with Pfizer-BioNTech

### Eman Badawod, Stephen Betschel

#### Division of Allergy and Clinical Immunology, St. Michael’s Hospital (Unity Health Toronto), University of Toronto, Toronto, ON

##### **Correspondence:** Eman Badawod

*Allergy, Asthma & Clinical Immunology* 2024, **20(Suppl 1):**68.

**Background:** Bullous pemphigoid (BP) is the most common autoimmune blistering skin disease. It is characterized by circulating autoantibodies toward the basement membrane antigens BP180 and BP230 [1]. Patients present with tense blisters on a background of underlying erythematous base. Different triggers have been identified for BP including medications, infections, malignancies, and vaccinations [2]. Post-vaccination BP is rare with a few case reports of new onset BP triggered by diphtheria, tetanus, influenza, pneumococcal, whooping cough, poliomyelitis, meningococcal, hepatitis B, BCG, and rabies vaccinations [3]. In patients with underlying autoimmune blistering skin disease there was evidence of a disease flare after receiving Pfizer-BioNTech vaccine during a remission period [4].

**Case presentation:** We describe the first case in Canada of a 54-year-old male who has no known medical problems and is not on any prescribed medications who presented four weeks following his 5th COVID booster with widespread erythematous rash. His family history was negative for significant diseases and skin disorders. He was initially treated with systemic corticosteroid (Prednisone 50 mg) however the rash spread to his perineum and inguinal folds, and he started to develop tense blisters. His Eosinophil count peaked at 2.33. A skin biopsy confirmed the diagnosis of BP, with direct positive serum immunofluorescence showing a linear band of C3 along the basement membrane. His prednisone dose was increased to 1 mg/kg. His course was complicated by superimposed bacterial infection with the growth of Staphylococcus aureus on blood cultures requiring further treatment with IV Cefazolin. A complete resolution of the blisters was obtained after 6 weeks of treatment.

**Conclusions:** A wide variety of delayed cutaneous reactions were reported in relation to COVID-19 vaccinations including erythema multiforme like eruption, eczematous eruptions, psoriasis exacerbation and urticarial vasculitis. It’s important to consider new onset BP following COVID-19 vaccinations in patients presenting with blisters.

All participants/their guardians gave written and/or oral informed consent to participate and to be published.

## 69 Food introduction in infant post-liver transplantation from deceased donor due to fatal anaphylaxis

### Ala El Baba, Julia Upton

#### University of Toronto, Toronto, ON

##### **Correspondence:** Ala El Baba

*Allergy, Asthma & Clinical Immunology* 2024, **20(Suppl 1):**69

**Background:** Transplant acquired food allergies (TAFA) are a well-documented phenomenon in Food Allergy literature. Many mechanisms for food allergy transfer have been postulated including direct transfer of allergen-specific IgE and lymphocytes. Risk factors include age of the recipient, type of organ transplanted and choice of immunosuppression. Guidance on food introduction in infants post solid-organ transplantation from donors deceased of fatal anaphylaxis however has not been well-described.

**Case presentation:** We present a 7-month-old female seen 10 days post-liver transplantation for biliary atresia. Her donor’s cause of death was anaphylaxis due to a suspected pine-nut exposure. Donor allergies listed included many priority allergens such as cow’s milk, soy, sesame, peanuts, treenuts, “gluten” (wheat) including legumes and fruits. No further information was accessible due to donor’s anonymity. Post-operatively, her diet consisted of elemental formula exclusively and therefore, the Allergy team was consulted to advise on food introduction given her risk factors for developing transplant acquired food allergies. She was receiving Tacrolimus, methylprednisolone and Benadryl which limited skin testing at the time. Allergen-specific IgE was drawn to each listed food allergen including pine-nuts which were negative (< 0.35 kU/l). We recommended introducing a single food allergen at each meal separated by a few hours to facilitate timely introduction of listed foods. For pine-nuts specifically, we suggested an oral graded challenge only after negative skin testing which would be facilitated as an outpatient with strict avoidance in the interim. Her parents were counselled to always carry an epinephrine autoinjector.

**Conclusions:** Management of transplant acquired food allergies can be challenging especially in the context of a donor deceased from fatal anaphylaxis, a restrictive list of suspected food allergies and limited information due to donor anonymity. Our approach would facilitate introduction of many listed food allergies without delays that could compromise growth and development of pediatric patients post-transplant.

All participants/their guardians gave written and/or oral informed consent to participate and to be published.

## 70 Two sides of the same coin: a case report of amoxicillin induced serum sickness like reaction (SSLR) and its mimicker the classic serum sickness

### Devyani Bakshi, Xinxin Tang, Susan Waserman

#### McMaster University, Hamilton, ON

##### **Correspondence:** Devyani Bakshi

*Allergy, Asthma & Clinical Immunology* 2024, **20(Suppl 1):**70

**Background:** Serum sickness-like reactions (SSLRs) are non-immune complex-mediated presentations characterized by rash, fever, and polyarthralgias [1]. SSLRs are triggered by viral infections, vaccines, and drugs such as amoxicillin [1, 2]. SSLRs differ from serum sickness (SS) in that they do not involve immune complexes and are not IgE-mediated [3]. SS and SSLRs share clinical features, hence distinguishing them can be difficult.

**Case presentation:** A 4-year-old boy presented to McMaster Children’s Hospital with a pruritic rash on day 7 of a 10-day course of amoxicillin for otitis media, accompanied by fever (38.7 degrees Celsius). His first course of amoxicillin was well tolerated and separated from his second course by an unknown time period. He developed erythematous, raised, pruritic sternal lesions with central clearing. He presented to hospital with emesis, progression of the rash to his torso, back, legs, and face, hypotension, angioedema, and difficulty ambulating.

His blood work demonstrated a leukocytosis of 18.6 × 109 g/L with neutrophilic predominance and thrombocytosis with a platelet count of 653 × 109 g/L.

He was treated with 5 mg oral cetirizine daily and 1 mg/kg oral prednisone which improved his rash and angioedema. The allergy and immunology consult service suggested up to 4 times the usual dose of second-generation antihistamines PRN for pruritus.

At the Adverse Drug Reactions clinic, skin testing to penicillin and ampicillin was negative; his parents decided to defer an oral challenge. A diagnosis of probable SSLR to amoxicillin was made.

**Conclusions:** Our case report demonstrates that patients with previous tolerance to Amoxicillin can develop SSLR with repeat exposure, thereby expanding our understanding of the range of adverse reactions which can be seen even with previously tolerated drugs. Healthcare providers need to remain vigilant to drug reactions and correctly diagnose SSLR, since safe reuse of Amoxicillin is possible even in this situation.

All participants/their guardians gave written and/or oral informed consent to participate and to be published.

## 71 Late presentation of X-linked inhibitor of apoptosis (XIAP) deficiency in a young adult

### Samina Nazarali, Beata Derfalvi, Lori Connors, Pascale Clark

#### Dalhousie University, Halifax, ON

##### **Correspondence:** Samina Nazarali

*Allergy, Asthma & Clinical Immunology* 2024, **20(Suppl 1):**71

**Background:** X-linked inhibitor of apoptosis (XIAP) deficiency is a rare inborn error of immunity which occurs secondary to mutations in the XIAP/BIRC4 gene. Disease onset usually manifests within the first few years of life, and is associated with a spectrum of clinical features, secondary to immune dysregulation. Males typically present with refractory chronic colitis, hemophagocytic lymphohistiocytosis, and severe and/or recurrent infections. Laboratory analysis may reveal hypogammaglobulinemia and cytopenias. At present, the only curative treatment is allogenic hematopoietic stem cell transplantation.

**Case presentation:** A 24-year-old gentleman, immigrant from the Democratic Republic of Congo, was referred to outpatient immunology for evaluation of an inborn error of immunity given a past medical history significant for refractory fistulizing Crohn’s disease, arthritis, liver abscesses, prior disseminated tuberculosis, anemia, and recurrent infections. He had been asymptomatic throughout his childhood and adolescence, with no infections or symptoms of inflammatory disease until the age of 19, when he was diagnosed with Crohn’s disease. He was soon after admitted to hospital and was diagnosed with hemophagocytic lymphohistiocytosis. Primary immunodeficiency gene panel testing revealed a nonsense variant XIAP c833C > G p.(Ser278*), which generates a premature stop codon at exon 2 (of total 7 exons). On flow cytometry analysis, XIAP protein expression was significantly reduced, confirming the diagnosis of XIAP deficiency.

**Conclusions:** This is one of the few documented reports of a patient with XIAP deficiency, presenting with symptom-onset in adulthood. This case highlights the need to maintain a high index of suspicion for XIAP deficiency in patients with the appropriate clinical presentation, despite advanced age of presentation.

All participants/their guardians gave written and/or oral informed consent to participate and to be published.

## 72 Drug rash with eosinophilia and systemic symptoms and co-sensitization to vancomycin and meropenem

### Mariam Eldaba^1^, Marie-Soleil Masse^2^, Ana-Maria Copaescu^2^, Jason Trubiano^3^

#### ^1^University of Ottawa, Ottawa, ON; ^2^Centre Hospitalier de l’Université de Montréal, Montréal, QC; ^3^Department of Infectious Diseases, University of Melbourne, at the Peter Doherty Institute for Infection and Immunity, Melbourne, VIC, Australia

##### **Correspondence:** Mariam Eldaba

*Allergy, Asthma & Clinical Immunology* 2024, **20(Suppl 1):**72

**Background:** Drug rash with eosinophilia and systemic symptoms (DRESS) syndrome is a severe cutaneous adverse reaction (SCAR) that occurs within 2–8 weeks following drug exposure. The management may prove challenging, particularly in patients with comorbidities requiring multiple medications. The optimal approach aims to identify the culprit drug with minimal restriction of potentially useful medications. In this study, we discuss the in vivo and ex vivo workup for a patient with DRESS syndrome associated with broad-spectrum antibiotics.

**Case presentation:** The patient is a 76-year-old male diagnosed with DRESS syndrome (RegiSCAR 5) following exposure to meropenem, vancomycin and rifampicin, received in the context of a polymicrobial paraspinal infection. Following the acute management and resolution of DRESS, in vivo drug allergy testing showed a positive delayed skin patch and intradermal test (IDT) to meropenem, a positive delayed IDT to vancomycin and the presence of HLA-A:32:01 associated with vancomycin DRESS. Delayed IDT tests for penicillins, amoxicillin, ceftriaxone, cefuroxime, and cefazolin were negative. Ex vivo testing using enzyme-linked ImmunoSpot (ELISpot) showed INF-y cytokine secretion following stimulation with meropenem and vancomycin. Based on his clinical timeline and investigation results, vancomycin and meropenem-induced DRESS was deemed most likely. He was counselled on avoidance of these drugs, whereas a drug re-introduction, following a patient risk–benefit discussion, may be considered for penicillins, cephalosporins and rifampin.

**Conclusions:** SCAR are often challenging to manage in the context of a serious infection and multiple drug exposures. Since drug avoidance has lasting implications, targeting culprit drugs with diagnostic accuracy is essential. Although rare, IDT testing could trigger SCAR relapse. Drug provocation tests are also contraindicated in SCAR. This case report describes a patient with DRESS and co-sensitization to vancomycin and meropenem and demonstrates the role of in vivo and ex vivo testing, including genetic testing.

All participants/their guardians gave written and/or oral informed consent to participate and to be published.

## 73 Recognition of Chronic Granulomatous Disease (CGD) in a teenage girl presenting with Inflammatory Bowel Disease (IBD) and recurrent *Staphylococcus lymphadenitis*

### Alicia Polack, Julia Upton

#### The Hospital for Sick Children, Toronto, ON

##### **Correspondence:** Alicia Polack

*Allergy, Asthma & Clinical Immunology* 2024, **20(Suppl 1):**73

**Background:** Chronic Granulomatous Disease is an uncommon hereditary primary immunodeficiency characterized by recurrent bacterial and fungal infections, intestinal disease, and granulomas. Defects in NADPH (nicotinamide-adenine-dinucleotide-phosphate) oxidase prevent formation of reactive oxygen species, an important process in killing microbes through phagocytosis, leading to increased susceptibility to catalase-positive organisms*.* X-linked CGD is more common, usually presenting in males during early childhood compared to autosomal recessive CGD which may present later in childhood or adulthood. Prompt treatment of infections and antimicrobial prophylaxis have improved prognosis however, hematopoietic stem cell transplantation (HSCT) is the current established curative therapy.

**Case presentation:** A 13-year-old girl presented with chronic abdominal pain, alternating bowel habits, and longstanding history of recurrent salivary gland enlargement and cervical lymphadenopathy. She was diagnosed with Crohn’s disease and started on infliximab, however one year later, developed cervical lymphadenitis. Nodal biopsy revealed necrotizing granulomatous and suppurative inflammation, cultures isolated *Staphylococcus aureus*, and a four-week course of cephalexin was started. A month later, she developed submental lymphadenitis on treatment and was switched to piperacillin-tazobactam. Neutrophil-oxidative-burst-index testing demonstrated low levels favouring a diagnosis of CGD. She was discharged on amoxicillin-clavulanate, trimethoprim-sulfamethoxazole, and prophylactic itraconazole but worsened two days later with abscess formation, requiring incision, drainage (I&D) plus meropenem for confirmed *Staphylococcus aureus* infection. Antibiotics were de-escalated to doxycycline and trimethoprim-sulfamethoxazole, but shortly after she developed recurrent symptoms necessitating prolonged hospitalization for intravenous cefazolin, levofloxacin and repeat I&Ds. Genetics confirmed pathogenic NCF1 (neutrophil-cytosolic-factor-1) variants supporting autosomal recessive CGD and HSCT was subsequently arranged.

**Conclusions:** This case highlights CGD as an etiology for IBD, even in a teenaged female. Moreover, it underscores that *Staphylococcus aureus* can be difficult-to-treat, requiring multiple agents for treatment of lymphadenitis. Although symptoms may overlap, increased suspicion for immunodeficiency despite atypical age of presentation and sex can facilitate earlier diagnosis and management, thereby preventing frequent and difficult-to-treat infections.

All participants/their guardians gave written and/or oral informed consent to participate and to be published.

## 74 Allergy recurrence post-oral immunotherapy in the preschool aged population, a case report

### Bryan C. Ng^1^, Deborah LeBlanc^2^, Scott B. Cameron^2,3^, Victoria E. Cook^2,3^

#### ^1^Faculty of Medicine, University of British Columbia, Vancouver, BC; ^2^Community Allergy Clinic, Victoria, BC’; ^3^Division of Allergy and Immunology, Department of Pediatrics, University of British Columbia, Vancouver, BC

##### **Correspondence:** Bryan C. Ng

*Allergy, Asthma & Clinical Immunology* 2024, **20(Suppl 1):**74

**Background:** Oral immunotherapy (OIT) is an emerging treatment approach for IgE-mediated food allergies. Current evidence suggests OIT is safe and effective for desensitization of food allergies in preschool-aged children [1, 2]. However, there is limited evidence describing long term outcomes in this population. We describe a case of symptom recurrence in a preschool-aged male after completion of OIT.

**Case presentation:** A 2-year-old male with allergist-diagnosed cashew allergy was treated with OIT. The highest grade of reaction prior to OIT initiation was a grade 2 systemic reaction managed with epinephrine. Baseline skin prick test (SPT) and serum specific IgE (ssIgE) values were 15 mm and 7.4 Ku/L, respectively. He spent 4 months on buildup and 12 months at a maintenance dose of 300 mg cashew protein, during which the only adverse reaction was oropharyngeal pruritus. Repeat testing was reduced at 0 mm and 5.7 kU/L, and he passed a 1-month sustained unresponsiveness food challenge of 4000 mg cashew protein. Cashew was eaten in small amounts regularly for 2–3 months after the challenge, but frequency and volume of cashew ingestion declined over time and parents reported infrequent ingestions of ≤ 300 mg of cashew protein prior to symptom recurrence 15-months after OIT completion. He experienced a grade 2 reaction of abdominal pain, conjunctivitis, hives, and oropharyngeal pruritus at recurrence. He was managed with a prescription for an epinephrine autoinjector and has restarted cashew OIT.

**Conclusions:** We describe a case of allergy recurrence after completion of OIT in a preschool-aged male. Our patient had persistently positive ssIgE at OIT completion and did not regularly ingest the food allergen. Symptoms at recurrence were mild and did not require epinephrine. Further research is required to determine the rate of allergy recurrence, identify risk factors for recurrence, and to optimize OIT protocols and follow-up to prevent recurrence.

All participants/their guardians gave written and/or oral informed consent to participate and to be published.

## 75 Hepatocellular carcinoma from danazol in a patient with hereditary angioedema

### Moss A. Bruton Joe, Anthony Cusano, Amin Kanani

#### University of British Columbia, Vancouver, BC

##### **Correspondence:** Moss A. Bruton Joe

*Allergy, Asthma & Clinical Immunology* 2024, **20(Suppl 1):**75

**Background:** Danazol is an attenuated androgen used in the long-term prophylaxis of hereditary angioedema (HAE). Danazol has hepatotoxic side effects including acute liver injury, hepatocellular necrosis, cholestasis, and hepatocellular adenoma. There are several case reports describing danazol-induced hepatocellular carcinoma (HCC) in patients with immune thrombocytopenia, systemic lupus erythematosus and endometriosis. However, at the dose used for HAE, risk of hepatotoxicity is considered low. There are only three other cases, to our knowledge, that describe HCC in patients taking danazol for HAE.

**Case presentation:** We present a case of a 70-year-old man with hereditary angioedema diagnosed at age 18. He was treated with danazol 100 mg twice daily for greater than 20 years for long-term prophylaxis. His medical history was notable for renal transplant secondary to diabetic nephropathy on tacrolimus, mycophenolate, and prednisone. His history was negative for alcohol use and viral hepatitis. On a routine annual screening ultrasound, the patient was found to have a well-circumscribed mass to the liver. A biopsy confirmed hepatocellular carcinoma, later staged as pT1b. At the time of diagnosis, his liver enzymes were normal except for a mild elevation in ALP, and his Alpha-1-Fetoprotein was normal. He was managed with a surgical resection of the liver mass and cholecystectomy alone. The resected liver parenchyma was negative for cirrhosis on pathology. A CT scan performed one year later showed no evidence of residual tumor, and no new lesions. Once diagnosed with HCC, the patient was switched from danazol to lanadelumab.

**Conclusions:** We report a case of HCC, a rare but serious toxicity from danazol in a patient with HAE. We hypothesize that concomitant immunosuppression in the setting of renal transplant may have increased his susceptibility to the development of HCC. Newer long-term prophylactic therapies with improved side effect profiles should be considered in patients with HAE.

All participants/their guardians gave written and/or oral informed consent to participate and to be published.

## 76 An uncommon culprit: anaphylaxis from rice hidden in beverages

### Moss A. Bruton Joe, Juan C. Ruiz

#### University of British Columbia, Vancouver, BC

##### **Correspondence:** Moss A. Bruton Joe

*Allergy, Asthma & Clinical Immunology* 2024, **20(Suppl 1):**76

**Background:** Anaphylaxis without an identifiable trigger presents a complex challenge in terms of diagnosis and management. Traditionally, the diagnostic process focuses on the most common food allergens which includes wheat but overlooks the potential involvement of other grains. Grains are increasingly prevalent in processed foods and can be concealed in vegan foods. Despite rice being a widely consumed staple food, reports of rice allergy remain relatively uncommon. Immediate IgE-mediated reactions following rice ingestion have been rarely documented, with fewer than 20 cases reported in the available literature.

**Case presentation:** We present a case report of a 43-year-old woman who experienced two episodes of anaphylaxis without an apparent trigger. On both occasions, the episodes occurred shortly after consuming breakfast, a protein shake and a sports recovery drink. The initial presentation occurred within thirty minutes, characterized by dyspnea, pre-syncope, and generalized pruritus, resolving with the administration of steroids and antihistamines. The second episode developed within minutes, presenting with neck angioedema, dyspnea, and pre-syncope, requiring two epinephrine injections. It was subsequently revealed that the protein shake contained rice protein, while the sports recovery drink contained brown rice syrup. There were no cofactors such as exercise or NSAIDS. We considered the possibility of a mast cell disorder, but her baseline tryptase was normal.

Skin prick testing yielded positive results to rice milk. To confirm the diagnosis, an oral challenge with rice milk was performed. Within ten minutes of ingesting 0.2 mL of rice milk, the patient developed hives and angioedema. Serum-specific IgE testing revealed positive results for wheat, buckwheat, and barley. The patient has since avoided rice and all other grains without experiencing anaphylaxis.

**Conclusions:** This case report serves as an illustration of anaphylaxis without an identifiable trigger, emphasizing the importance of considering uncommon allergens and concealed sources during the diagnostic evaluation process.

All participants/their guardians gave written and/or oral informed consent to participate and to be published.

## 77 Chocolate allergy and immunotherapy

### Jumana U. Sarraj, Lina Thabit, Harold Kim

#### Western University, London, ON

##### **Correspondence:** Jumana U. Sarraj

*Allergy, Asthma & Clinical Immunology* 2024, **20(Suppl 1):**77

**Background:** Chocolate allergy is rare and not well studied. Majority of defined chocolate/cocoa allergies are thought to be due to cross contamination from common allergens that are present in store-available chocolate including dairy, tree nuts, and peanuts. The American Academy of Asthma and Immunology warned that chocolate is notorious for having hidden allergens.

**Case presentation:** 20-year-old male with no medical history or medications comes in for chocolate allergy reassessment. At age 3 ingested unknown chocolate bar with immediate anaphylaxis. Remote skin prick testing positive to chocolate and tree nuts. So he avoided these foods. He tolerates peanuts and dairy. Repeat skin testing was positive to cashew, pistachio, walnut and pecan. Fresh food skin testing was negative to chocolate. IgE testing was positive to cacao, almond, cashew, hazelnut, walnut. He passed oral challenge to almonds and incorporated into diet. He failed oral challenge to chocolate after ingesting 15 g of 70% Lindt dark chocolate (2 g of protein). He has started chocolate oral immunotherapy with Hershey’s cacao powder capsules.

**Conclusions:** While many allergic reactions to chocolate likely are attributed to contaminants or hidden ingredients, true cocoa allergy exists with case reports in the literature. There is no specific data to suggest appropriate serving size for chocolate based oral challenges or desensitization protocol data published. Our patient is receiving desensitization with Hershey’s cocoa powder which is free of contaminants. Future plans include repeat skin testing, IgE testing and oral challenge at end of oral immunotherapy.

All participants/their guardians gave written and/or oral informed consent to participate and to be published.

## Other allergy/immunology

## 78 Specificity comparison of penicillin de-labelling tools: PENFAST, JAMA, and FIRSTLINE in a pregnant population

### Joanne Wang^1^, Chelsea Elwood^1^, Vanessa Paquette^3^, Natasha Kwan^3^, Stephanie Erdle^1^, Melissa Watt^2^, Julie Van Schalkwyk^1^, Jeffrey Bone^4^, Ashley Roberts^1^, Raymond Mak^1^, Tiffany Wong^1^

#### ^1^University of British Columbia, Vancouver, BC; ^2^Women’s Health Research Institute, BC Women’s Hospital, Vancouver, BC; ^3^Children’s and Women’s Health Centre of British Columbia, Vancouver, BC; ^4^BC Children’s Hospital Research Institute, Vancouver, BC

##### **Correspondence:** Joanne Wang

*Allergy, Asthma & Clinical Immunology* 2024, **20(Suppl 1):**78

**Background:** Penicillin allergy adversely impacts patient care, yet most cases do not have true allergies. Due to the high prevalence of reported penicillin allergy, clinicians require an efficient, reliable clinical tools to identify low-risk patients who can be safely de-labeled. We compare the diagnostic test performances of three risk stratification tools: FIRSTLINE, JAMA, and PENFAST, to assess their ability to to accurately identify low-risk patients appropriate for direct oral challenge.

**Methods:** In this single-center, retrospective, observational study, a cohort of 181 pregnant females with self-reported penicillin allergy between July 2019 to June 2021 from BC Women’s Hospital, Vancouver, Canada was used to assess the sensitivity of the three penicillin decision tools. Their self-reported history of the penicillin use and symptoms were used for scoring. Scoring results and recommendations were compared to actual patient outcomes after direct oral challenge or intradermal tests.

**Results:** From our cohort, FIRSTLINE had the highest specificity (sp = 86.6%; 95%CI 81.7%–91.6%) compared to PENFAST (sp = 82.8; 95%CI 77.3%–88.3% and JAMA (sp = 60.5%; 95%CI 53.3%–67.6%).

**Conclusions:** To our knowledge, this is the first study to provide direct specificity comparison between PENFAST, JAMA, and FIRSTLINE whereby using the same population minimizes participant bias. Providing clinical algorithms to accurately identify patients with low-risk penicillin allergy can enable healthcare professionals to safely risk stratify individuals to direct penicillin oral challenges versus referral to specialists. This can generalize penicillin allergy de-labelling and increase efficiency.

## 79 Implementation of antibiotic prescribing reports in a pediatric emergency department: a quality improvements study

### Carsten Krueger^1^, Waleed Alqurashi^1^, Nicholas Barrowman^2^, Maria Litwinska^3^, Nicole Le Saux^1^

#### ^1^Children’s Hospital of Eastern Ontario (CHEO), Ottawa, ON; ^2^Children’s Hospital of Eastern Ontario Research Institute, Ottawa, ON; ^3^Business Intelligence Team, Information Services, Children’s Hospital of Eastern Ontario, Ottawa, ON

##### **Correspondence:** Waleed Alqurashi

*Allergy, Asthma & Clinical Immunology* 2024, **20(Suppl 1):**79

**Background:** Most antibiotics prescribed to children are provided in the outpatient and emergency department (ED) settings, yet these prescribers are seldom engaged in antibiotic stewardship programs (ASP). We implemented an intervention to optimize antibiotic prescriptions for common infections leveraging anonymous peer-to-peer comparison.

**Methods:** In January 2022, we presented contemporary literature at ED grand rounds on antibiotic durations, data summary of ED antibiotics prescribing between 2018–2021, and introduced the antibiotic prescribing report (APR) program. These APRs included Pareto charts highlighting key contributors to prescriptions given with durations longer than minimally recommended (LMR) and details around department-level prescribing practices. A pre-implementation APR using prescription data from Quarter 4 2021 was circulated. Prescription data were extracted quarterly between January 1st 2022 and December 31st 2022 from the records of children discharged from our ED with urinary tract infection (UTI), community-acquired pneumonia (CAP), acute otitis media (AOM; ≥ 2 years of age), and cellulitis. These data were used to construct subsequent semi-annual APRs. Changes in prescribing over the intervention period were described, and department-level reductions in antibiotic use were estimated compared with 2021 prescribing patterns.

**Results:** At the year-end after implementation of the reports, 3017 prescriptions were included (582 UTI, 871 CAP, 1344 AOM, and 220 cellulitis diagnoses. In total, there were 2421 antibiotic-days less after the APR program, resulting in a decrease in total antibiotic use for these infections by 11.5%. The proportion of prescriptions with LMR durations for each infection decreased in the post-implementation period (2021 vs 2022), corresponding with statically significant decreases in antibiotic-days prescribed normalized per 1000 infection-specific visits (UTI 7759 vs 6576, CAP 7123 vs 6100, AOM 6374 vs 5825, cellulitis 6826 vs 6359).

**Conclusions:** Implementing an APR program in addition to peer-to-peer comparison charts is a simple, resource-effective way to greatly reduce antibiotic exposure of children diagnosed with common infections in ED.

## 80 Pilot mobile allergy teaching unit in Rwanda

### Michelle Kwok^1^, Ghislaine A. Isabwe^1,2^

#### ^1^Division of Allergy and Clinical Immunology, Department of Medicine, McGill University Health Center, McGill University, Montréal, QC; ^2^The Research Institute of McGill University Health Center, McGill University, Montréal, QC

##### **Correspondence:** Michelle Kwok

*Allergy, Asthma & Clinical Immunology* 2024, **20(Suppl 1):**80

**Background:** Asthma and atopy are rapidly becoming significant causes of mortality and morbidity in Africa. However, they are largely undiagnosed and undertreated due to both poor access to allergist care and remarkably few allergists on the African continent [1]. Data on anaphylaxis is extremely limited with no register-based African studies [2]. One small cohort study showed that most anaphylaxis episodes were managed solely by a layperson and epinephrine was rarely administered even by healthcare professionals [3]. We conducted a pilot allergy teaching unit in Rwanda, which currently has no local postgraduate allergy-immunology training program. Our main objective was to train medical residents to recognize and appropriately treat anaphylaxis, atopy, and asthma.

**Methods:** We conducted a one-week intensive allergy training course at the University of Rwanda which was integrated into the local curriculum. Seventeen senior medical residents in internal medicine, emergency medicine, otolaryngology, and dermatology attended. All materials were based on Canadian guidelines and modified for the Rwandan context, with instruction in English, supplemented by French and Kinyarwanda. Didactic lectures were given on immunodeficiency, allergic rhinitis, asthma, drug allergy, urticaria, food allergy, venom allergy and anaphylaxis. These were followed by objective structured clinical examinations and a final exam.

**Results:** All residents were required to complete a survey to assess the effectiveness of this course. Overall, 16/17 (94%) of residents rated this course well (4–5/5), with main benefits being increased confidence in managing atopic conditions in their everyday practice. The main criticism was insufficient time allocation.

**Conclusions:** Rwanda, like many other African nations, requires its own allergy and immunology program. Until then, our pilot teaching project can help fill the knowledge gap by instructing medical residents to recognize and treat anaphylaxis and other allergic conditions. Eventually, we hope that future Rwandan allergists can create their locally based and internationally standardized registries.

**References**

1. The Global Asthma Report 2022. *Int J Tuberc Lung Dis* 2022;26(1):1–104.
2. Kung SJ, Steenhoff AP, Gray C. Food allergy in Africa: myth or reality? *Clin Rev Allergy Immunol* 2014;46(3):241–9. 10.1007/s12016-012-8341-z
3. Chippendale SE, Reichmuth K, Worm M, et al. Paediatric anaphylaxis in South Africa. *World Allergy Organ J* 2022;15(9):100,666. 10.1016/j.waojou.2022.100666 [published Online First: 20220912]

## 81 Skin testing and challenge in patients with immediate hypersensitivity reactions to gadolinium-based contrast agents identify safe future options

### Florian Stehlin^1,2^, Rabea Y. Khoudja^1,3^, Derek Lee^4^, Toupin Jean-François^5^, Ghislaine A. Isabwe^1,3^, Ibtihal Al-Otaibi^1,6^, Elizabeth J. Phillips^7,8^, Jason A. Trubiano^9,10,11^, Ana-Maria Copaescu^1,3,12^

#### ^1^Division of Allergy and Clinical Immunology, Department of Medicine, McGill University Health Center (MUHC), McGill University, Montreal, Montreal, QC; ^2^Division of Clinical Immunology and Allergy, Department of Medicine, Centre Hospitalier Universitaire Vaudois (CHUV), Lausanne, Switzerland; ^3^The Research Institute of McGill University Health Center, McGill University, Montreal, QC; ^4^Pharmacy Department, Montreal General Hospital, Montreal, QC; ^5^Department of Diagnostic Radiology, McGill University Health Centre (MUHC), McGill University, Montreal, QC; ^6^Division of Allergy and Clinical Immunology, Department of Paediatrics, Central Second Health Cluster, Ministry of Health, Riyadh, Saudi Arabia; ^7^Center for Drug Safety and Immunology, Vanderbilt University Medical Centre, Nashville, TN, USA; ^8^Institute for Immunology and Infectious Diseases, Murdoch University, Murdoch, WA, Australia; ^9^Center for Antibiotic Allergy and Research, Department of Infectious Diseases, Austin Health, Heidelberg, VIC, Australia; ^10^The National Centre for Infections in Cancer, Peter MacCallum Cancer Centre, Parkville, VIC, Australia; ^11^Department of Infectious Diseases, University of Melbourne, at the Peter Doherty Institute for Infection and Immunity, Melbourne, VIC, Australia; ^12^Department of Medicine, Austin Health, The University of Melbourne, Heidelberg, VIC, Australia

##### **Correspondence:** Florian Stehlin

*Allergy, Asthma & Clinical Immunology* 2024, **20(Suppl 1):**81

**Background:** Gadolinium-based contrast agents (GBCA) used for magnetic resonance imaging differ physicochemically, can be macrocyclic or linear, and rarely induce immediate hypersensitivity reactions (IHR) [1]. We assessed the performance and safety of GBCA skin testing and drug provocation testing (DPT) in patients with GBCA IHR.

**Methods:** Undiluted skin prick testing (SPT) and intradermal testing (IDT) with 1:10 dilution [2] were performed with gadobutrol, gadoteridol (both macrocyclic), gadobenate and gadoxetate (both linear). A placebo-controlled (normal saline), 2-step (1 ml intravenous (IV) followed, if negative, by 4 ml IV) DPT with GBCA was performed following negative skin testing.

**Results:** Out of 8 patients, 5 had a history of anaphylaxis to a GBCA (patients 1, 3, 4, 7, 8; Ring-Messmer grade III for patients 1 and 3). Patients 2, 5 and 6 had non-anaphylactic IHR (urticarial, angioedema). Patient 1, who reacted to gadobenate, had positive SPT and IDT to the latter with otherwise negative GBCA SPT/IDT and tolerated gadobutrol DPT. Patient 2, where gadobenate was implicated, had negative GBCA IDT and tolerated gadobutrol DPT. Patient 3, who reacted to gadoteridol, had a positive gadoteridol IDT. Gadobutrol IDT and DPT were negative. Patient 4, where gadoteridol was implicated, had positive IDT to gadoteridol and gadobutrol. Gadoxetate and gadobenate IDT were negative. Gadobenate DPT was tolerated. Patient 5, where gadoteridol was implicated, had negative IDT and DPT to gadoteridol. Patient 6, who reacted to gadobutrol, had negative gadobutrol IDT and DPT. Patients 7 and 8, where gadobutrol was implicated, had negative GBCA IDT. Upon gadobutrol and gadoteridol DPT respectively, they developed mild urticaria self-resolving in less than one hour, suggesting a non-IgE mediated reaction.

**Conclusions:** This study highlights the value of IDT with diverse GBCA, even after life-threatening IHR, to identify a tolerated GBCA. We propose a simple and safe 2-step DPT that can be easily implemented in clinical allergy practice.

**References**

1. Vega, F., et al., *Fast challenge tests with gadolinium-based contrast agents to search for an alternative contrast media in allergic patients.* Allergy, 2022. **77**(10): p. 3151–3153.
2. Chiriac, A.M., et al., *Clinical value of negative skin tests to gadolinium contrast agents.* Allergy, 2011. **66**(11): p. 1504–6.

## 82 Introduction of an inpatient penicillin allergy de-labelling program with direct oral challenge: a multicenter parallel cohort with crossover study

### Adhora Mir^1^, Derek Lanoue^2^, Carl van Walraven^1,3^, Timothy Olynych^1^, Veronica Zanichelli^3^, Caroline Nott^1,3^, Derek Macfadden^1,3^

#### ^1^University of Ottawa, Ottawa, ON; ^2^McGill University, Montreal, QC; ^3^The Ottawa Hospital Research Institute, Ottawa, ON

##### **Correspondence:** Adhora Mir

*Allergy, Asthma & Clinical Immunology* 2024, **20(Suppl 1):**82

**Background:** Reported penicillin allergies are common and often inaccurate, leading to reduced use of first-line beta-lactam antibiotics and worse outcomes. Direct oral challenge of penicillin-based antibiotics has been shown to be safe and effective at de-labelling adult inpatients with remote, low-risk, cutaneous-only reactions. We evaluated the impact of a novel inpatient penicillin allergy de-labelling program with oral amoxicillin challenge on penicillin allergy delabelling and subsequent antibiotic use.

**Methods:** A retrospective comparison of parallel cohorts from two separate tertiary care hospital campuses across two penicillin de-labelling intervention periods was conducted. Outcomes, including penicillin allergy labelling and antibiotic use, were collected for the index admission and the subsequent 6-month period. Descriptive statistics and multivariate regression analyses were performed.

**Results:** Among 368 patients with penicillin allergy label were included across two campuses and study periods, 24 (13.8%) patients in the intervention groups had sustained penicillin allergy label removal at 30 days from admission vs. 3 (1.5%) in the non-intervention group (p < 0.001). Of the 24 patients de-labelled in the intervention arm, 19 had received a direct oral challenge and 5 were directly de-labelled based on history. When multivraiate analysis was applied, we found that beta-lactams were prescribed more frequently in the intervention groups vs. the non-intervention groups for all patients (OR 2.49, 95%CI 1.29–5.02) and in those prescribed at least one antibiotic (OR 2.44, 95%CI 1.00–6.15) in the 6 months following index admission. No drug-related adverse events were reported.

**Conclusions:** The penicillin allergy de-labelling intervention was associated with a reduction in penicillin allergy labels and increased utilization of beta-lactams in the subsequent 6-months. These results support the adoption of a proactive penicillin allergy de-labelling program to aid low-risk penicillin allergy de-labelling with direct oral challenge and support beta-lactam antibiotic use where indicated.

## 83 Development of a virtual clinical decision support tool for penicillin allergy de-labelling

### Joshua V. Yu^1^, Derek Lanoue^2^, Adhora Mir^3^, Rongbo Zhu^4^, Harold Kim^4,5^

#### ^1^Department of Medicine, Faculty of Medicine, McMaster University, Hamilton, ON; ^2^Division of Allergy and Clinical Immunology, Montreal General Hospital, McGill University Health Centre, Montreal, QC; ^3^Department of Medicine, Faculty of Medicine, University of Ottawa, Ottawa, ON; ^4^Division of Clinical Immunology and Allergy, Department of Medicine, Western University, London, ON; ^5^Division of Clinical Immunology and Allergy, Department of Medicine, McMaster University, Hamilton, ON

##### **Correspondence:** Joshua V. Yu

*Allergy, Asthma & Clinical Immunology* 2024, **20(Suppl 1):**83

**Background:** Patient-reported penicillin allergies are common but often inaccurate, leading to suboptimal antibiotic selection, worse patient outcomes, and increased healthcare expenses. Limited availability of Allergy-Immunology subspecialists and uncertainty among non-allergist physicians contribute to difficulty in assessing these allergies.

**Methods:** We aimed to develop an accessible virtual clinical decision tool for accurately assessing the risk of true penicillin allergy and appropriateness for a challenge based on the previously validated PEN-FAST decision rule. Additionally, we used OpenAI’s large language model to create a preliminary assessment chatbot for patients.

**Results:** We created a user-friendly website [https://penicillinallergy.ca/] with a risk stratification tool for patients and healthcare providers that automatically generates a personalized referral note, including a preliminary assessment that is generated for their personal doctor. It also offers a physician-led protocol for direct oral challenges in low-risk patients and high-quality resources for patients and physicians. We also developed a high-fidelity proof-of-concept chatbot using Chat-GPT-3.5 to guide patients through the decision tool.

**Conclusions:** Penicillin allergy labels are prevalent but often inaccurate. Enhancing accessibility to user-friendly tools for providers and patients to evaluate these labels and determine the need for further testing could substantially impact antibiotic stewardship, patient outcomes, and healthcare costs. This tool will be used in a future QI initiative to test the feasibility of primary care de-labelling.

## 84 Real-time monitoring of Fel d 1 concentrations during controlled cat allergen exposure in the SPaC-EEU

### Lubnaa Hossenbaccus^1^, Terry Walker^2^, Anne K. Ellis^1,2^

#### ^1^Queen’s University, Kingston, ON; ^2^Kingston Health Sciences Centre—KGH Site, Kingston, ON

##### **Correspondence:** Lubnaa Hossenbaccus

*Allergy, Asthma & Clinical Immunology* 2024, **20(Suppl 1):**84

**Background:** The Specialized Particulate Control Environmental Exposure Unit (SPaC-EEU) is a controlled allergen challenge clinical model of allergic rhinitis (AR) designed for perennial allergens, located at the Kingston Health Sciences Centre—Kingston General Hospital site. It has been previously validated for house dust mite and recently underwent a successful technical validation for cat allergen, specifically *Felis domesticus allergen 1* (Fel d 1). We sought to investigate the relationship between the laser particle counter (LPC) device and Fel d 1 concentrations collected from sampling cassettes.

**Methods:** Cat dander allergen (Greer®, USA) was dispersed into the SPaC-EEU during four runs, each between 1.5 to 2 h in length. The LPC recorded particles sized 2.5, 5.0, 10.0, 15.0, 20.0, and 25.0 µm at 10 s intervals during the runs. Air samples were collected using sampling cassettes (Zefon International, USA) at 30 to 120-min intervals. Fel d 1 concentrations were quantified from sampling cassettes using a Fel d 1-specific ELISA (Indoor Biotechnologies, USA).

**Results:** Fel d 1 concentrations across the four runs in the SPaC-EEU were not significantly different (mean: 51.58 ng/m^3^, 75.94 ng/m^3^, 53.99 ng/m^3^, 69.39 ng/m^3^). Similar trends were observed between Fel d 1 concentrations from sampling cassettes and particle counts from the LPC in the runs when both were overlayed graphically. Fel d 1 concentrations were significantly and positively correlated (p < 0.05; r = 0.6071) with average particle counts at corresponding timepoints.

**Conclusions:** The LPC can be used to indirectly monitor Fel d 1 concentrations in real-time during cat dander exposure in the SPaC-EEU. This enables greater control and consistency of dispersed allergen in the SPaC-EEU while ensuring participants’ safety.

## 85 Clinical characteristics and management of food protein induced enterocolitis syndrome (FPIES) in Canadian children

### Angela Mulé, Pasquale Mulé, Adnan Al Ali, Catherine Prattico, Xun Zhang, Christine McCusker, Moshe Ben-Shoshan

#### Division of Allergy and Clinical Immunology, Department of Pediatrics, Montreal Children’s Hospital, McGill University Health Centre, Montreal, QC

##### **Correspondence:** Angela Mulé

*Allergy, Asthma & Clinical Immunology* 2024, **20(Suppl 1):**85

**Background:** Data on food protein induced enterocolitis syndrome (FPIES) are sparse. We aimed to evaluate sociodemographic characteristics, co-morbidities, and triggers of children presenting with FPIES, as well as their tolerance to baked goods.

**Methods:** Children with physician-diagnosed FPIES were enrolled and followed at the Montreal Children’s Hospital and an affiliated clinic. Families were queried on the food trigger and co-morbidities, as well as clinical characteristics of reaction and management. A severe reaction was defined as vomiting 4 or more times, altered behaviour/lethargy, pallor, dehydration, and need for IV fluids [1].

**Results:** Between November 2021 and June 2023, 31 children with a confirmed history of FPIES were enrolled. Patient ages ranged from 1 to 167 months old (median 7 months), and 45% were males. Food triggers included egg (32%), milk (26%), oat (23%), shellfish (10%), fish (10%), soy/grains (6%), fruit (6%), peanut (6%), and rice (3%). Six patients (19%) had 2 or more food triggers. Co-morbidities included asthma (3 patients, 10%) and eczema (15 patients, 48%). The most common symptoms were vomiting (98%), lethargy (21%), pallor (18%), dehydration (13%), and diarrhea (10%). Symptoms developed in the majority of cases within 1–4 h of exposure. Seven patients (18%) had a severe reaction. Among fifteen patients who tried baked goods, nine (60%) were tolerant.

**Conclusions:** The most common food triggers are egg and milk. The majority of reactions are not severe and baked goods containing the culprit food are often tolerated.

**Reference**

1. Nowak-Węgrzyn A, Chehade M, Groetch ME, et al. International consensus guidelines for the diagnosis and management of food protein-induced enterocolitis syndrome: Executive summary-Workgroup Report of the Adverse Reactions to Foods Committee, American Academy of Allergy, Asthma & Immunology. *J Allergy Clin Immunol*. 2017;139(4):1111-1126.e4. 10.1016/j.jaci.2016.12.966

## 86 Can we predict true penicillin allergy in a pediatric population?

### Joshua Yu^2^, Derek Lanoue^1^, Adhora Mir^3^, Andrew O’Keefe^4^, Christine McCusker^8^, Elissa M. Abrams^5^, Thomas Eiwegger^6,7^, Adelle Atkinson^7^, Vy Kim^7^, Ana-Maria Copaescu^1^, Moshe Ben-Shoshan^8^

#### ^1^Division of Allergy and Clinical Immunology, Montreal General Hospital, McGill University Health Centre, Montreal, QC; ^2^Department of Medicine, Faculty of Medicine, McMaster University, Hamilton, ON; ^3^Department of Medicine, Faculty of Medicine, University of Ottawa, Ottawa, ON; ^4^Department of Pediatrics, Faculty of Medicine, Memorial University, St. John’s, NL; ^5^Department of Pediatrics and Child Health, Section of Allergy and Clinical Immunology, Children’s Hospital Research Institute of Manitoba, Winnipeg, MB; ^6^Department of Pediatric and Adolescent Medicine, University Hospital St. Pölten, Karl Landsteiner University of Health Sciences, Krems an der Donau, Austria; ^7^Division of Clinical Immunology and Allergy, Department of Pediatrics, The Hospital for Sick Children, University of Toronto, Toronto, ON; ^8^Division of Allergy and Clinical Immunology, Department of Pediatrics, Montreal Children’s Hospital, McGill University Health Centre, Montreal, QC

##### **Correspondence:** Derek Lanoue

*Allergy, Asthma & Clinical Immunology* 2024, **20(Suppl 1):**86

**Background:** Pediatric self-reported penicillin/penicillin derivatives allergies are 10%. However, most tolerate drug challenges. Previous attempts to validate predictive models have yet to be successful. We aimed to determine indicators of true amoxicillin allergy using an extensive multicenter Canadian database.

**Methods:** We assessed data collected on children referred for suspected amoxicillin allergy using a Canadian multicenter prospective pediatric cohort. All participants were challenged to amoxicillin. Reactions were defined as immediate versus non-immediate based on symptoms onset within or after one hour of challenge. Over 100 different patient and index reaction characteristics were analyzed. Backwards stepwise logistic regression and Random Forest machine learning models were employed to validate predictive factors for challenge-confirmed amoxicillin allergy.

**Results:** Among 2,379 children, 118 (5%) had a positive oral challenge, with 54 (2.3%) immediate reactions and 64 (2.7%) non-immediate reactions. All reactions were mild. Multivariate logistic regression and Random Forest models yielded AUCs of approximately 0.7 for composite, immediate and non-immediate reactions. For composite reactions, a history of chronic spontaneous urticaria (CSU) had the highest adjusted odds ratio (aOR 7.1, 95% CI 2.8–16.3, p < 0.001) and > 1 year between index reaction and challenge had the lowest aOR 0.46, 0.24–0.84, p < 0.001. Positive predictors for immediate reactions were CSU and index reaction gastrointestinal symptoms. Parental drug allergy history, index reaction pruritus, treatment in an emergency department, and CSU were significant predictors for non-immediate reactors. Our cohort contained limited high-risk patients; however, 16 patients received epinephrine during index reaction, and of those, only 2/16 (13%) had positive oral challenges with mild skin-limited symptoms.

**Conclusions:** Although we identified several predictors for positive challenges, it was not possible to construct an appropriate predictive model. Given the low rate of positive reactions and absence of severe reactions, oral challenges should continue to be offered to nearly all children with suspected penicillin allergy.

## 87 Medical resource utilization and quality of life of HAE patients based on data from the 2020 national survey

### Jacquie L.-Badiou^1^, Michelle Cooper^1^, Daphne Dumbrille^1^, Suzanne M. Kelly^2^, Chrystyna Kalicinsky^3^, Paul K. Keith^4^, Gina Lacuesta^5^, Amin Kanani^6^, William H. Yang^2,7^

#### ^1^HAE Canada, Ottawa, ON; ^2^Red Maple Trials Inc, Ottawa, ON; ^3^University of Manitoba, Winnipeg, MB; ^4^McMaster University, Hamilton, ON; ^5^Dalhousie University, Halifax, NS; ^6^University of British Columbia, Vancouver, BC; ^7^University of Ottawa, Ottawa, ON

##### **Correspondence:** Jacquie L.-Badiou

*Allergy, Asthma & Clinical Immunology* 2024, **20(Suppl 1):**87

**Background:** Hereditary angioedema (HAE) is a rare genetic disease characterized by unpredictable, recurrent episodes of angioedema leading to swelling of limbs, face, larynx and the gastrointestinal tract. Episodes are painful and can be fatal. Most patients have deficient or dysfunctional C1 inhibitor (HAE C1INH), but a significant percentage have other mutations causing similar episodes of angioedema (HAEnC1INH).

**Methods:** Because randomized controlled trials may not fully reflect HAE patients’ burden of illness, HAE Canada has conducted multiple surveys to obtain data which would otherwise be unavailable. Data obtained includes demographics, number and severity of attacks, treatment utilization and satisfaction, quality of life (QoL), burden of illness, health care utilization and economic costs to the patient.

**Results:** Results from our 2020 survey show that despite 88% of HAE patients using HAE medication, a significant proportion still have > 12 attacks/year (HAE C1INH: 27%, HAEnC1INH: 50%) and unscheduled visits to the ER (HAE C1INH: 45%, HAEnC1INH: 54%), missed work (HAE C1INH: 53%, HAEnC1INH: 61%) and have high levels of anxiety (HAE C1INH: 61%, HAEnC1INH: 67%). Medications are evolving but due to heterogeneity of treatment effects, more treatment options are needed for attack prevention, acute attack management as well as more convenient treatment modalities (e.g., oral or subcutaneous versus intravenous). Better treatments and better access to treatment may offset costs to the health care system and improve patient QoL.

**Conclusions:** Our data has been used to raise awareness of HAE and to advocate for access to treatment. However, there are still unmet needs and further research is needed to better manage all forms of angioedema. A registry to collect real-world data would be highly beneficial.

## 88 Treatment outcomes for up to 3 years in patients aged less than 12 years with inadequately controlled moderate-to-severe atopic dermatitis: real-world data from PEDISTAD

### Amy S. Paller^1,2^, Dedee F. Murrell^3^, Constance H. Katelaris^4,5^, Marlies de Graaf^6^, Danielle Marcoux^7,8^, Eulalia Baselga^9^, Vania Oliveira de Carvalho^10^, Ledit R. Ardusso^11^, Mirna Toledo-Bahena^12^, Rajan Gupta^13^, Marius Ardeleanu^14^, Annie Zhang^13^

#### ^1^Northwestern University Feinberg School of Medicine, Chicago, IL, USA; ^2^Ann and Robert H. Lurie Children’s Hospital, Chicago, IL, USA; ^3^St George Hospital, University of New South Wales, Sydney, NSW, Australia; ^4^Campbelltown Hospital, Campbelltown, NSW, Australia; ^5^Western Sydney University, Sydney, NSW, Australia; ^6^University Medical Centre, Utrecht, Netherlands; ^7^University of Montreal, Montreal, QC; ^8^CHU Sainte-Justine University Hospital Center, Montreal, QC; ^9^Hospital Sant Joan de Déu, Barcelona, Spain; ^10^Clinical Hospital of the Federal University of Paraná, Curitiba, PA, Brazil; ^11^National University of Rosario, Rosario, Argentina; ^12^Hospital Infantil de México Federico Gómez, Mexico City, MEX, Mexico; ^13^Sanofi, Cambridge, MA, USA; ^14^Regeneron Pharmaceuticals Inc., Tarrytown, NY, USA

##### **Correspondence:** Amy S. Paller

*Allergy, Asthma & Clinical Immunology* 2024, **20(Suppl 1):**88

**Background:** In phase 3 studies, dupilumab significantly improved disease severity in patients with moderate-to-severe atopic dermatitis (AD); however, less is known about the impact of systemic treatments on children in real-world treatment settings.

**Methods:** PEDISTAD (NCT03687359) is an ongoing, international, observational study in patients aged < 12 years with moderate-to-severe AD inadequately controlled by topical therapies or for whom those therapies are inadvisable. This analysis assesses the effect of systemic AD treatments dupilumab, cyclosporine, and methotrexate on Eczema Area and Severity Index (EASI) total score, %-affected body surface area (BSA) and adverse events (AEs).

**Results:** 211 patients received dupilumab (mean treatment observation period: 16.9 months), 130 received methotrexate (19.2 months), 142 received cyclosporine (14.6 months). The most common baseline type 2 inflammatory comorbidities were food allergies for patients treated with dupilumab (50.7%), methotrexate (39.2%), and cyclosporine (42.3%). EASI scores significantly improved with dupilumab (treatment start = 19.7, last observation = 6.1; *P* < 0.0001), methotrexate (17.1, 9.6; *P* = 0.0005) and cyclosporine (18.5, 13.7; *P* < 0.0001). BSA scores also significantly improved with dupilumab (38.3%, 17.1%; *P* < 0.0001), methotrexate (34.8%, 20.5%; *P* < 0.0001), and cyclosporine (39.5%, 29.4%; *P* = 0.0001). AE rates were 24.2% dupilumab, 30.3% methotrexate, and 33.3% cyclosporine whilst accumulated 3-year discontinuation rates were 13.3% for dupilumab, 36.9% for methotrexate, and 56.3% for cyclosporine.

**Conclusions:** Among analysis groups, dupilumab was associated with numerically greater improvement in AD signs and symptoms, with less treatment discontinuation and lower AE rates than cyclosporine and methotrexate.

## 89 Long-term laboratory safety of dupilumab in patients aged 6 months to 5 years with moderate-to-severe atopic dermatitis

### Elaine C. Siegfried^1,2^, Michael J. Cork^3^, Benjamin Lockshin^4^, Mark Boguniewicz^5,6^, Sumeet Uppal^7^, Guy Gherardi^8^, Ashish Bansal^7^, Debra Sierka^7^, Sonya Cyr^7^

#### ^1^Saint Louis University, St Louis, MO, USA; ^2^Cardinal Glennon Children’s Hospital, St Louis, MO, USA; ^3^University of Sheffield, Sheffield, United Kingdom; ^4^Georgetown University, Washington, DC, USA; ^5^National Jewish Health, Denver, CO, USA; ^6^University of Colorado School of Medicine, Denver, CO, USA; ^7^Regeneron Pharmaceuticals Inc., Tarrytown, NY, USA; ^8^Sanofi, Reading, United Kingdom

##### **Correspondence:** Elaine C. Siegfried

*Allergy, Asthma & Clinical Immunology* 2024, **20(Suppl 1):**89

**Background:** Systemic treatments often require ongoing laboratory monitoring. Here we report 52-week laboratory safety data for dupilumab-treated children aged 6 months to 5 years with moderate-to-severe atopic dermatitis (AD).

**Methods:** LIBERTY AD PED-OLE (NCT02612454) is an open-label extension study of children aged 6 months to < 18 years with moderate-to-severe AD. This analysis includes haematological and chemistry laboratory parameters in children aged 6 months to 5 years treated with dupilumab every 4 weeks (q4w; 200 mg: ≥ 5 kg to < 15 kg; 300 mg: ≥ 15 kg to < 30 kg).

**Results:** Of the 180 patients enrolled, 122 (67.8%) completed up to 16 weeks and 30 (16.7%) completed up to 52 weeks. Mean (SD) eosinophil counts increased slightly from baseline (1.15 × 10^9^/L [1.18]) to Week 16 (1.48 × 10^9^/L [1.91]), but then decreased below baseline by Week 52 (0.80 × 10^9^/L [0.64]). Mean (SD) platelet counts were relatively stable with a modest decrease from baseline (388.7 × 10^9^/L [102.51]) to Week 52 (356.1 × 10^9^/L [107.48]). Mean (SD) leukocyte counts increased slightly from baseline (9.76 × 10^9^/L [3.51] to Week 16 (10.00 × 10^9^/L [3.35]), before decreasing below baseline at Week 52 (8.48 × 10^9^/L [2.57]). Chemistry parameters remained within the normal reference ranges at Week 16. One patient (0.6%) reported a mild case of anaemia, and one patient (0.6%) reported a mild case of thrombocytopenia, which were resolving and resolved at the time of this interim analysis, respectively. Overall safety was consistent with the known dupilumab safety profile.

**Conclusions:** No clinically meaningful changes in haematological and chemistry parameters were observed during 52 weeks of dupilumab treatment. As with adults, adolescents and older children, these results indicate no need for routine laboratory monitoring in children aged 6 months to 5 years treated with dupilumab for moderate-to-severe AD.

## 90 Results of a pilot collaborative dermatology/allergy pediatric patch testing clinic at BC Children’s Hospital

### Ella K. Chan^1^, Sarah Lohrenz^2^, Wingfield Rehmus^3^, Stephanie C. Erdle^4^

#### ^1^Faculty of Medicine, University of British Columbia, Vancouver, BC; ^2^Division of Allergy and Clinical Immunology, Department of Medicine, University of British Columbia, Vancouver, BC; ^3^Division of Dermatology, Department of Pediatrics, University of British Columbia, Vancouver, BC; ^4^Division of Allergy and Immunology, Department of Pediatrics, University of British Columbia, Vancouver, BC

##### **Correspondence:** Ella K. Chan

*Allergy, Asthma & Clinical Immunology* 2024, **20(Suppl 1):**90

**Background:** In April 2023, a novel pediatric patch testing clinic was launched at BC Children’s Hospital (BCCH) as a collaboration between dermatology and allergy. Prior to this, no such pediatric clinic existed in British Columbia.

**Methods:** To pilot this clinic, we started with a cohort of 6 patients who were referred by either a dermatologist or allergist for assessment of possible allergic contact dermatitis (ACD). The North American 80 Comprehensive Series (NA80) was used for testing. Baseline characteristics and patch testing results were collected.

**Results:** 6 patients were enrolled in the first clinic. The median age was 11 years (range: 9–18 years).

3 patients were referred from BCCH dermatology, 2 from BCCH allergy, and 1 from community pediatric allergy. 2 (33%) patients had a suspected ACD trigger (Band-Aid and sunscreen), and 4 (67%) patients had suspected ACD (moderate-severe atopic dermatitis that was uncontrolled or difficult to control).

3 (50%) had additional atopy: 2 with IgE-mediated food allergies, and 3 with allergic rhinoconjunctivitis.

5 (83%) patients tolerated the patch testing, with the patches falling off one (17%) patient within 24 h. 4/5 (80%) patients had positive testing to one or more ingredients. The patient with a suspected Band-Aid allergy had a positive reaction to colophony, a band-aid ingredient. The patient with a suspected sunscreen allergy had negative testing, and will return for repeat testing to various sunscreens. 4/5 (80%) patients experienced indeterminate/irritation reactions. 1/5 (20%) of patients found their results to be inconsistent with their suspected trigger, suggesting possible false positive results.

**Conclusions:** This is the first collaborative pediatric dermatology/allergy patch testing clinic in British Columbia, and to our knowledge in Canada. Our pilot clinic identified potential ACD triggers in 80% of patients referred with suspected ACD. Future steps will involve creating a ACD registry at BCCH to further evaluate pediatric patients with ACD.

## Urticaria/angioedema

## 91 Baseline characteristics and lanadelumab treatment outcomes in Canadian patients with hereditary angioedema: Interim 24-month data from the real-world EMPOWER Study

### Stephen D. Betschel^1^, Hugo Chapdelaine^2,3^, Remi Gagnon^4^, M. Dawn Goodyear^5^, Paul K. Keith^6^, Ahmed El-Zoeiby^7^, Natalie Khutoryansky^8^, Daniel Nova Estepan^8^

#### ^1^Clinical Immunology and Allergy, University of Toronto, Toronto, ON; ^2^Centre Hospitalier de l’Université de Montréal, Université de Montréal, Montréal, QC; ^3^Montréal Clinical Research Institute, Montréal, QC; ^4^Laval University, Centre Hospitalier Universitaire de Québec (CHUQ), Québec, QC; ^5^Cumming School of Medicine, Department of Medicine, Division of Hematology and Hematologic Malignancies, University of Calgary, Calgary, AB; ^6^McMaster University, Department of Medicine, Division of Clinical Immunology and Allergy, Hamilton, ON; ^7^Takeda Canada Inc., Toronto, ON; ^8^Takeda Development Center Americas, Inc., Lexington, MA, USA

##### **Correspondence:** Paul K. Keith

*Allergy, Asthma & Clinical Immunology* 2024, **20(Suppl 1):**91

**Background:** The EMPOWER Study (NCT03845400) is a Phase IV observational, non-interventional, multicenter study evaluating real-world effectiveness and safety of lanadelumab in patients with HAE from Canada and US. We report outcomes in the Canadian patients from EMPOWER.

**Methods:** Patients with HAE Type I/II could be enrolled in EMPOWER. This interim analysis included patients with ≥ 1 lanadelumab dose and ≥ 1 post-baseline effectiveness assessment who were new or prevalent (< 4 or ≥ 4 lanadelumab doses before enrollment, respectively) lanadelumab users.

**Results:** As of March 1, 2022, 3/15 new and 2/87 prevalent lanadelumab users were from Canada. Mean ± SD age in new and prevalent Canadian users was 48.0 ± 10.2 and 62.5 ± 6.4 years, respectively, versus 40.6 ± 17.6 in EMPOWER overall. All Canadian patients were White, had HAE Type I, and most (66.7% new, 100% prevalent) were female, in agreement with EMPOWER overall (94.1% White, 76.5% HAE Type I, 65.7% female). Mean (95% CI) HAE attack rate in new Canadian users was 0.02 (0.01–0.03) attacks/month pre-lanadelumab and 0.03 (0.02–0.04) post-lanadelumab initiation; and 0.00 (0.00–0.00) in prevalent Canadian users during the study. In EMPOWER overall, mean (95% CI) attack rate in new users was 1.07 (0.93–1.23) attacks/month pre-lanadelumab and 0.22 (0.18–0.27) post-lanadelumab initiation; and 0.16 (0.15–0.18) in prevalent users. The majority of HAE attacks in Canadian EMPOWER patients were mild-to-moderate in severity, treated with plasma-derived C1 inhibitor, and did not require visits to healthcare professionals. There was 1 treatment-emergent adverse event (TEAE) in 1 new and 8 TEAEs in 1 prevalent Canadian user; all non-severe, non-serious, and not related to lanadelumab.

**Conclusions:** In this interim analysis, Canadian patients from the real-world EMPOWER Study had higher age but otherwise similar demographic characteristics to overall EMPOWER population. Effectiveness and safety were consistent with interim results from EMPOWER overall.

## 92 Remibrutinib (LOU064) versus placebo in CSU patients: study design of the two Phase 3 clinical trials REMIX-1 and REMIX-2

### Gordon Sussman

#### University of Toronto, Toronto, ON

##### **Correspondence:** Gordon Sussman

*Allergy, Asthma & Clinical Immunology* 2024, **20(Suppl 1):**92

**Background:** Remibrutinib is a novel, highly selective and potent, oral covalent BTKi with demonstrated efficacy, fast onset of action and favourable safety in CSU patients in a Phase 2b clinical study. We report the study design of REMIX-1(NCT05030311) and REMIX-2 (NCT05032157), two Phase 3 clinical trials, in CSU patients inadequately controlled by second-generation H1-antihistamines.

**Methods:** REMIX-1 and REMIX-2 are multicenter, randomised, double-blind, parallel-group, placebo-controlled, Phase 3 clinical trials conducted in parallel to evaluate the efficacy, safety, and tolerability of remibrutinib vs placebo in adult CSU patients. Studies consist of four periods, total duration of up to 60 weeks: Screening period (up to 4 weeks), double-blind treatment period (remibrutinib/placebo; 24 weeks), open-label treatment period (remibrutinib; 28 weeks) and treatment-free follow-up period (4 weeks). The studies include adult patients with CSU duration of ≥ 6 months, inadequately controlled (presence of itch and hives for ≥ 6 consecutive weeks and weekly Urticaria Activity Score [UAS7; range: 0–42] ≥ 16, weekly Itch Severity Score [ISS7; range: 0–21] ≥ 6 and weekly Hives Severity Score [HSS7; range 0–21] ≥ 6 during the 7 days prior to randomisation) by second-generation H1-antihistamines. Two primary objective scenarios will be tested independently: the first as primary endpoint of change from baseline in UAS7 at Week 12, and the second as co-primary endpoints of change from baseline in ISS7 and HSS7 at Week 12 (depending on regional precedent and Health Authority feedback). The study population will consist of approximately 450 patients (per trial) randomised in a 2:1 ratio to receive remibrutinib (n = 300) or placebo (n = 150).

**Results:** The enrolment was initiated in Q4 2021 and the studies are expected to be completed in 2024.

**Conclusions:** The results of the REMIX studies will provide further evidence to demonstrate the efficacy and safety of remibrutinib in CSU.

## 93 Hereditary alpha-tryptasemia prevalence in a pediatric chronic urticaria population

### Vanessa Ceccato^1^, Luca Delli Colli^1^, Najmah Almuhsen^2^, Sofianne Gabrielli^1^, Elena Netchiporouk^3^, Christine McCusker^1^, Moshe Ben-Shoshan^1^

#### ^1^Division of Allergy, Immunology and Dermatology, Montreal Children’s Hospital, Montreal, QC; ^2^Division of Medical Genetics, McGill University Health Centre, Montreal, QC; ^3^Division of Dermatology, McGill University, Montreal, QC

##### **Correspondence:** Vanessa Ceccato

*Allergy, Asthma & Clinical Immunology* 2024, **20(Suppl 1):**93

**Background:** Hereditary alpha-tryptasemia (HaT) is an autosomal dominant genetic trait that has been associated with chronic urticaria (CU). Data assessing the prevalence of HaT in pediatric patients with chronic urticaria is sparse. We aimed to estimate the prevalence of HaT in children with CU.

**Methods:** Pediatric patients with chronic urticaria presenting to the Montreal Children’s Hospital were recruited as part of the Chronic Urticaria, Thyroiditis, Autoimmunity, and Natural History Registry (CUTAN-R) from April 2013 to July 2023. Data on demographics, comorbidities, types of hives and family health history were collected. Additionally, serum tryptase levels were measured in all patients. Genetic assessment for HaT was conducted for children with levels above the normal (11.4 mcg/l) (Gene by Gene, Houston, Tx, USA).

**Results:** Of the 339 patients recruited, 48.1% were male. The median age of symptom onset was 10.0 years old (IQR 10.00). Among all, 17.4% had spontaneous CU, 82.6% had inducible forms and none had coexisting spontaneous and inducible forms. Approximately 15.0% of patients had asthma, while the same percentage had atopic dermatitis. Treatment involved antihistamines for 78.2% of patients and omalizumab for 4.1%. A single patient exhibited an elevated tryptase level of 13.7 mcg/l, surpassing the average cohort tryptase level of 3.9 mcg/l. HaT diagnosis was established through genetic testing, which revealed an additional copy of alpha tryptase encoded by the TPSAB1 gene. Genetic assessment for first degree family members revealed three more cases with HaT: the father (intermittent hives), the brother (intermittent itchiness and hives), and the sister (recurrent syncope and ketotic hypoglycemia). The patient’s hives were well controlled with omalizumab.

**Conclusions:** The prevalence of HaT among pediatric patients with CU is low. However, when an index case is diagnosed, genetic testing for other family members may lead to the diagnosis of additional cases in the family.

## 94 Comparison of the diagnosis and management of hereditary angioedema in urban and rural populations

### Ashley Holmes^1^, Cindy Srinivasan^2^, Jack Borle^1^, Bruce Ritchie^3^, Adil Adatia^4^

#### ^1^Faculty of Science, University of Alberta, Edmonton, AB; ^2^Faculty of Medicine, University of Alberta, Edmonton, AB; ^3^Division of Hematology, Department of Medicine, University of Alberta, Edmonton, AB; ^4^Division of Pulmonary Medicine, Department of Medicine, University of Alberta, Edmonton, AB

##### **Correspondence:** Ashley Holmes

*Allergy, Asthma & Clinical Immunology* 2024, **20(Suppl 1):**94

**Background:** Hereditary angioedema (HAE) is a genetic disorder characterized by recurrent swelling of the bowel mucosa, extremities, and upper airway. The complexity of HAE together with Canada’s expansive geography and considerable rural population make provision of high-quality HAE care challenging.

**Methods:** A cross-sectional study of adult HAE patients examining demographic, diagnostic and treatment parameters between urban and rural patients was conducted. Patient data were collected retrospectively via chart review from a tertiary care HAE center. The Canadian Bleeding Disorder Registry supplied additional information on patient use of plasma-derived C1 inhibitor (pd-C1). StatsCan census data were used to determine patient rurality based on a subdivision population threshold of < 10,000.

**Results:** Among 62 patients with HAE type I and II, 62.9% and 37.1% resided in urban and rural areas, respectively. Majority were female in both groups comprising 76.9% of urban and 60.9% of rural patients. Average age was similar at 43.5 and 43.4 years for urban and rural patients, respectively. The proportion diagnosed by 18 was similar (41.0% [95% CI: 27.1, 56.6%] in urban and 52.2% [33.0, 70.8%] in rural patients). Use of long-term prophylaxis (LTP) was comparable at 64.1% (48.4, 77.3%) for urban and 60.9% (40.7, 77.9%) for rural patients. The predominant LTP agent was pd-C1 in both groups at 64% (44.4, 79.8%) for urban and 64.3% (38.6, 83.8%) for rural patients. Likewise, the primary on-demand agent was pd-C1 used in 89.7% and 87.0% of urban and rural patients, respectively.

**Conclusions:** This is the first Canadian study comparing rural and urban HAE patient populations and care. No significant differences in the demographics, age of diagnosis, or use of LTP were identified. A centralized, comprehensive care model supported by digital blood product tracking and rural blood bank distribution networks can help overcome major geographic barriers in HAE care provision.

## 95 Effectiveness of berotralstat for the prevention of hereditary angioedema attacks–first real-world evidence from Canada

### Cindy Srinivasan^1^, Bruce Ritchie^2^, Adil Adatia^3^

#### ^1^Faculty of Medicine, University of Alberta, Edmonton, AB; ^2^Division of Hematology, Department of Medicine, University of Alberta, Edmonton, AB; ^3^Division of Pulmonary Medicine, Department of Medicine, University of Alberta, Edmonton, AB

##### **Correspondence:** Cindy Srinivasan

*Allergy, Asthma & Clinical Immunology* 2024, **20(Suppl 1):**95

**Background:** Hereditary angioedema (HAE) is a rare genetic disease that leads to painful swelling of the upper airway, gastrointestinal tract, and extremities. Long-term prophylaxis (LTP) to prevent angioedema episodes is the cornerstone of modern disease management. Berolostat, an oral plasma kallikrein inhibitor, was approved for LTP by Health Canada in 2022.

**Methods:** We conducted a retrospective study examining the effectiveness and adverse effects of berotralstat in a real-world setting. Data on attack frequency, disease control, and adverse events before and after starting berotralstat were collected. Disease control was measured using the Angioedema Control Test (AECT), a validated patient-reported instrument with scores between 0 to 16, with higher values indicating better control [1]. Patient satisfaction with treatment was scored on a 5-point Likert scale with 1 representing very unsatisfied and 5 representing very satisfied with therapy.

**Results:** Between June, 2022 and May, 2023, 8 patients with HAE type 1 or type 2 were prescribed berotralstat 150 mg once daily. Effectiveness data were available for 7 patients who continued the drug for at least 3 months, 4 of whom switched to berotralstat from plasma-derived C1 inhibitor LTP. In these 7 patients, the average number of attacks per month decreased from 3.3 to 1.6 (p < 0.05), representing a ~ 52% reduction in attack frequency, similar to the 57% reduction observed in a phase 3 registration trial [2]. Median AECT score numerically improved from 8 to 13 (p = 0.0781). Of the 8 patients who started berotralstat, 3 reported no adverse effects and 5 experienced gastrointestinal adverse effects, which were mild or transient in 3 and led to discontinuation in 1. Average treatment satisfaction was between satisfied and very satisfied at 4.3.

**Conclusions:** Berotralstat is an effective agent for long-term prophylaxis in HAE. Most patients experienced no adverse effects or mild, transient gastrointestinal symptoms.

**References**

1. Weller K, Donoso T, Magerl M, et al. Development of the Angioedema Control Test-A patient-reported outcome measure that assesses disease control in patients with recurrent angioedema. *Allergy*. 2020;75(5):1165–1177.
2. Zuraw B, Lumry WR, Johnston DT, et al. Oral once-daily berotralstat for the prevention of hereditary angioedema attacks: A randomized, double-blind, placebo-controlled phase 3 trial. *J Allergy Clin Immunol.* 2021;148(1):164-172e9.

## 96 Retrospective chart review of four patients with hereditary angioedema with specific plasminogen gene mutation

### Arun Dhir, Shamim Wadiwalla, Amin Kanani

#### Division of Allergy and Immunology, Department of Medicine, University of British Columbia, Vancouver, BC

##### **Correspondence:** Arun Dhir

*Allergy, Asthma & Clinical Immunology* 2024, **20(Suppl 1):**96

**Background:** Hereditary angioedema with a specific mutation in the plasminogen gene (HAE-PLG) is a recently identified subtype of hereditary angioedema (HAE) characterized by bradykinin-mediated angioedema [1]. It is inherited in an autosomal dominant manner, and women are more commonly affected than men.

**Methods:** We conducted a retrospective chart review of four patients with HAE-PLG, confirmed by genetic testing, who were undergoing treatment in British Columbia, Canada.

**Results:** All four patients were female, with three patients belonging to the same family. The average age of symptom onset was 27.5 years (range: 19–51 years), and the average age at diagnosis was 42.75 years (range: 26–56 years). All patients experienced oropharyngeal attacks. One patient required two ICU admissions and one intubation. Three patients (75%) reported symptoms related to exogenous estrogen. One patient experienced more frequent symptoms during pregnancy. Two patients (50%) reported more frequent attacks after menopause.

One patient has responded well to on-demand antihistamines alone, suggesting concurrent idiopathic histamine-mediated angioedema. Another patient received a combination of corticosteroids and antihistamines with potential benefit. Two patients received plasma-derived C1-esterase with good response. These two patients have also responded well to icatibant. One patient had a partial response to tranexamic acid. One patient has been initiated on berotralstat, with ongoing evaluation of its effectiveness.

**Conclusions:** While it was previously believed that estrogens played a less significant role in HAE-PLG compared to patients with specific mutations in the F12 gene (HAE-FXII) [1, 2], our patients reported a high rate of estrogen-associated symptoms. Two patients also reported extensive menopause-related angioedema, which has not been described previously. A variety of treatment options have been used in our cohort, with icatibant and plasma-derived C1-INH appearing to be particularly effective. Future research is needed to further comprehend and improve the management of HAE-PLG.

**References**

1. Bork K, Wulff K, Steinmüller-Magin L, Brænne I, Staubach-Renz P, Witzke G, et al. Hereditary angioedema with a mutation in the plasminogen gene. Allergy. 2018;73(2):442–50.
2. Bork K, Machnig T, Wulff K, Witzke G, Prusty S, Hardt J. Clinical features of genetically characterized types of hereditary angioedema with normal C1 inhibitor: a systematic review of qualitative evidence. Orphanet J Rare Dis. 2020 Oct 15;15(1):289.

## 97 Antihistamines for the management of chronic urticaria: a systematic review of randomized controlled trials

### Alexandro W. Chu^1^, Irene X. Zhao^1^, Andy Zhu^2^, Layla Bakaa^3^, Xiajing Chu^4^, Ming Liu^4^, David M. Lang^5^, Moshe Ben-Shoshan^6^, Susan Waserman^1^, Sameer K. Mathur^7^, Lisa A. Beck^8^, Eric T. Oliver^9^, Diane Baker^10^, David A. Khan^11^, Javed Sheikh^12^, Joseph Moellman^13^, Jonathan A. Bernstein^14^, Sarbjit S. Saini^9^, Derek K. Chu^1,4^, for the AAAAI/ACAAI Joint Task Force on Practice Parameters Chronic Urticaria Guideline Development Group

#### ^1^Department of Medicine, McMaster University, Hamilton, ON; ^2^Temerty Faculty of Medicine, University of Toronto, Toronto, ON; ^3^Department of Epidemiology, Harvard TH Chan School of Public Health, Boston, MA, USA; ^4^Department of Health Research Methods, Evidence, and Impact, McMaster University, Hamilton, ON; ^5^Department of Allergy and Clinical Immunology, Respiratory Institute, Cleveland Clinic Foundation, Cleveland, OH, USA; ^6^Division of Allergy and Clinical Immunology, Department of Pediatrics, McGill University Health Centre, Montréal, QC; ^7^Division of Allergy, Pulmonary & Critical Care Medicine, Department of Medicine, School of Medicine & Public Health, University of Wisconsin, Madison, WI, USA; ^8^Department of Dermatology, Medicine and Pathology, University of Rochester Medical Center, Rochester, NY, USA; ^9^Division of Allergy and Clinical Immunology, Department of Medicine, Johns Hopkins University School of Medicine, Baltimore, MD, USA; ^10^Baker Allergy, Asthma and Dermatology, Portland, OR, USA; ^11^Department of Internal Medicine, Allergy and Immunology, The University of Texas Southwestern Medical Center, Dallas, TX, USA; ^12^Department of Allergy and Immunology, Kaiser Permanente Los Angeles Medical Center, Los Angeles, CA, USA; ^13^Department of Emergency Medicine, University of Cincinnati College of Medicine, Cincinnati, OH, USA; ^14^Division of Immunology-Allergy Section, Department of Internal Medicine, University of Cincinnati College of Medicine, Cincinnati, OH, USA

##### **Correspondence:** Alexandro W. Chu

*Allergy, Asthma & Clinical Immunology* 2024, **20(Suppl 1):**97

**Background:** Chronic urticaria is a condition characterized by pruritus and the development of wheals, angioedema, or both, for 6 weeks or more. Antihistamines are well-established for treating chronic urticaria, however, the comparative benefits and harms of different antihistamines are uncertain.

**Methods:** As part of the upcoming AAAAI/ACAAI Joint Task Force on Practice Parameters Chronic Urticaria Guidelines, we systematically identified randomized controlled trials addressing antihistamines for chronic urticaria from inception to February 19, 2023 from MEDLINE, EMBASE, and CENTRAL. Additionally, we conducted forward and backward citation analysis using the Web of Science and reviewed drug monographs to identify relevant trials. Paired reviewers screened titles and abstracts, reviewed full texts, and extracted data independently and in duplicate. We extracted study and participant characteristics for each trial. We registered the review prospectively in PROSPERO, CRD42022367280.

**Results:** We included 190 trials enrolling 20,459 participants (pediatrics and adults) followed for a median of 4 weeks (range 0.57–12), investigating 50 unique antihistamines across 38 countries. 27 (14%) trials addressed dosing of antihistamines. The median of mean ages across studies for participants was 38 years (range 4–60), and females made up 61% of the participants (range 21–100). Of the included trials, 160 (84%) included participants with chronic spontaneous urticaria, and 27 (14%) included participants with chronic inducible urticaria. 176 (93%) trials reported a measure of efficacy, such as overall urticaria severity, itch scores, wheal scores, or angioedema activity; 27 (14%) reported a measure of urticaria-related quality of life; 150 (79%) reported adverse event data; and 120 (63%) reported data addressing drowsiness or somnolence.

**Conclusions:** The availability of such data permits conducting network meta-analyses to determine the comparative patient-important benefits and harms of all unique antihistamines in the management of chronic urticaria; the results of which will inform optimal care among patients and clinicians.

***Alexandro W. Chu—CSACI Summer Studentship Award winner***

